# Sertraline for anxiety in adults with a diagnosis of autism (STRATA): study protocol for a pragmatic, multicentre, double-blind, placebo-controlled randomised controlled trial

**DOI:** 10.1186/s13063-023-07847-3

**Published:** 2024-01-11

**Authors:** Dheeraj Rai, Doug Webb, Amanda Lewis, Leonora Cotton, Jade Eloise Norris, Regi Alexander, David S. Baldwin, Traolach Brugha, Madeleine Cochrane, Maria Chiara Del Piccolo, Emma J. Glasson, Katherine K. Hatch, David Kessler, Peter E. Langdon, Helen Leonard, Stephanie J. MacNeill, Nicola Mills, Maximiliano Vazquez Morales, Zoe Morgan, Raja Mukherjee, Alba X. Realpe, Ailsa Russell, Sergio Starkstein, Jodi Taylor, Nicholas Turner, Joanna Thorn, Jack Welch, on behalf of the STRATA autistic advisory group, Sarah Douglas, Peter Hale, Sarah O’Brien, Amy Walker, Nicola Wiles

**Affiliations:** 1 Population Health Sciences, University of Bristol, Bristol, UK; 2 NIHR Bristol Biomedical Research Centre, Bristol, UK; 3 Avon & Wiltshire Partnership Mental Health NHS Trust, Bath, UK; 4 Bristol Trials Centre, University of Bristol, Bristol, UK; 5 Hertfordshire Partnership NHS Foundation Trust, Hatfield, UK; 6 Clinical and Experimental Sciences, Faculty of Medicine, University of Southampton, Southampton, UK; 7 University of Leicester, Leicester, UK; 8 Surrey and Borders Partnership NHS Foundation Trust, Leatherhead, UK; 9 Telethon Kids Institute, The University of Western Australia, Perth, Australia; 10 Discipline of Psychiatry, Medical School, The University of Western Australia, Perth, Australia; 11 Centre for Research in Intellectual and Developmental Disabilities, University of Warwick, Coventry, UK; 12 Coventry and Warwickshire Partnership NHS Trust, Coventry, UK; 13 Centre for Applied Autism Research, Department of Psychology, University of Bath, Bath, UK; 14 Dorset County Hospital NHS Foundation Trust, Dorchester, UK

**Keywords:** Autism, Asperger, Anxiety, Adults, Antidepressant, Mental health, Sertraline, Selective Serotonin reuptake inhibitors, Placebo, Randomised controlled trial

## Abstract

**Background:**

Selective serotonin reuptake inhibitors (SSRIs) are commonly prescribed to manage anxiety in adults with an autism diagnosis. However, their effectiveness and adverse effect profile in the autistic population are not well known. This trial aims to determine the effectiveness and cost-effectiveness of the SSRI sertraline in reducing symptoms of anxiety and improving quality of life in adults with a diagnosis of autism compared with placebo and to quantify any adverse effects.

**Methods:**

STRATA is a two-parallel group, multi-centre, pragmatic, double-blind, randomised placebo-controlled trial with allocation at the level of the individual. It will be delivered through recruiting sites with autism services in 4 regional centres in the United Kingdom (UK) and 1 in Australia. Adults with an autism diagnosis and a Generalised Anxiety Disorder Assessment (GAD-7) score ≥ 10 at screening will be randomised 1:1 to either 25 mg sertraline or placebo, with subsequent flexible dose titration up to 200 mg. The primary outcome is GAD-7 scores at 16 weeks post-randomisation. Secondary outcomes include adverse effects, proportionate change in GAD-7 scores including 50% reduction, social anxiety, obsessive-compulsive symptoms, panic attacks, repetitive behaviours, meltdowns, depressive symptoms, composite depression and anxiety, functioning and disability and quality of life. Carer burden will be assessed in a linked carer sub-study. Outcome data will be collected using online/paper methods via video call, face-to-face or telephone according to participant preference at 16, 24 and 52 weeks post-randomisation, with brief safety checks and data collection at 1–2, 4, 8, 12 and 36 weeks. An economic evaluation to study the cost-effectiveness of sertraline vs placebo and a QuinteT Recruitment Intervention (QRI) to optimise recruitment and informed consent are embedded within the trial. Qualitative interviews at various times during the study will explore experiences of participating and taking the trial medication.

**Discussion:**

Results from this study should help autistic adults and their clinicians make evidence-based decisions on the use of sertraline for managing anxiety in this population.

**Trial registration:**

ISRCTN, ISRCTN15984604. Registered on 08 February 2021. EudraCT 2019-004312-66. ANZCTR ACTRN12621000801819. Registered on 07 April 2021.

**Supplementary Information:**

The online version contains supplementary material available at 10.1186/s13063-023-07847-3.

## Administrative information

Note: the numbers in curly brackets in this protocol refer to SPIRIT checklist item numbers. The order of the items has been modified to group similar items (see http://www.equator-network.org/reporting-guidelines/spirit-2013-statement-defining-standard-protocol-items-for-clinical-trials/).

**Table:** 

Title {1}	Sertraline for anxiety in adults with a diagnosis of autism (STRATA): Study protocol for a pragmatic, multicentre, double-blind, placebo-controlled randomised controlled trial.
Trial registration {2a and 2b}.	ISRCTN: 15984604 (08-Feb-2021); ANZCTR: ACTRN12621000801819 (07-Apr-2021).
Protocol version {3}	Version 7.0, 31 August 2023.
Funding {4}	National Institute for Health Research Health Technology Assessment (NIHR127337); National Health and Medical Research Council (NHMRC GNT1171206).
Author details {5a}	*Dheeraj Rai*^*1,2,3*^*, Doug Webb*^*1,4*^*, Amanda Lewis*^*1,4*^*, Leonora Cotton*^*1,4*^*, Jade Eloise Norris*^*1,4*^*, Regi Alexander*^*5*^*, David S Baldwin*^*6*^*, Traolach Brugha*^*7*^*, Madeleine Cochrane*^*1,4*^*, Maria Chiara Del Piccolo*^*8*^*, Emma J Glasson*^*9,10*^*, Katherine K Hatch*^*10*^*, David Kessler*^*1,2*^*, Peter E Langdon*^*11, 12*^*, Helen Leonard*^*9,10*^*, Stephanie J MacNeill*^*1,2,4*^*, Nicola Mills*^*1,2*^*, Maximiliano Vazquez Morales,*^*1,4*^*, Zoe Morgan*^*7*^*, Raja Mukherjee*^*8*^*, Alba X Realpe*^*1,2,4*^*, Ailsa Russell*^*13*^*, Sergio Starkstein*^*10*^*, Jodi Taylor*^*1,4*^*, Nicholas Turner*^*1,4*^*, Joanna Thorn*^*1,4*^*, Jack Welch*^*14*^ on behalf of the STRATA autistic advisory group*, Nicola Wiles^*1,2*^*.* 1. Population Health Sciences, University of Bristol 2. NIHR Bristol Biomedical Research Centre 3. Avon & Wiltshire Partnership Mental Health NHS Trust 4. Bristol Trials Centre, University of Bristol 5. Hertfordshire Partnership NHS Foundation Trust 6. Clinical and Experimental Sciences, Faculty of Medicine, University of Southampton 7. University of Leicester 8. Surrey and Borders Partnership NHS Foundation Trust 9. Telethon Kids Institute, The University of Western Australia 10. Discipline of Psychiatry, Medical School, The University of Western Australia 11. Centre for Research in Intellectual and Developmental Disabilities, University of Warwick 12. Coventry and Warwickshire Partnership NHS Trust 13. Centre for Applied Autism Research, Department of Psychology, University of Bath 14. Dorset County Hospital NHS Foundation Trust
Name and contact information for the trial sponsor {5b}	Adam Taylor, Head of Research Governance, Research and Enterprise Development, University of Bristol, Senate House Tyndall Ave, Bristol, BS8 1TH. Telephone: 0117 42 83065. Email: research-governance@bristol.ac.uk Australia Centre Sponsor: The University of Western Australia (M459), 35 Stirling Highway, Crawley, Perth, Western Australia 6009. Telephone: + 61 8 6488 4260.
Role of sponsor {5c}	The sponsor is responsible for research governance and providing oversight to the study as defined in the UK Policy Framework for Health and Social Care Research. The sponsor or study funders are not involved in study design or in collection, management, analysis, or interpretation of data, or the decision to submit for publication.

## Introduction

### Background and rationale {6a}

Autism spectrum disorder (henceforth autism) is a developmental condition characterised by differences in social interaction and communication [1]. Autistic adults, particularly those without intellectual disabilities (ID), have a greater burden of mental health problems than the general population [2–6], and higher rates of premature mortality, with suicide as an important contributor [7]. Despite the need, there is an absence of high-quality randomised controlled trial (RCT) evidence in relation to interventions for mental health problems in the adult autistic population [8].

Anxiety is common in autistic adults [2, 3, 5, 6], and the distress and avoidance behaviours related to it can be severely disabling. The reported rates of anxiety disorders and related conditions in adults with an autism diagnosis vary widely (28–77%) because most research has been conducted with selected clinical samples, and a recent meta-analysis reported a pooled lifetime prevalence of 42% [2]. Social phobia, generalised anxiety disorder and obsessive–compulsive disorder (OCD) are common diagnoses, but anxiety in autistic people often does not align with the rigid diagnostic criteria for individual anxiety disorders [3, 9]. The causes of the increased prevalence of anxiety in autistic people are multifactorial and likely a combination of biological, psychosocial and environmental factors [10]. Anxiety in autistic individuals may be managed by ensuring consistency in the environment, minimising sensory overload or sudden changes in routines or plans. There is some evidence in support of cognitive behavioural therapies [8]. However, it is also not uncommon for autistic individuals to seek, or be offered, medication options for managing anxiety.

Selective serotonin reuptake inhibitors (SSRIs) are commonly used antidepressants but are also first-line medications for all anxiety disorders [11]. The antidepressant action of SSRIs starts within 1 week, with statistical separation from placebo often evident in 2 to 4 weeks, but it is thought that effects on anxiety disorders may take longer [12]. However, findings from a recent RCT in the United Kingdom (UK) primary care population suggested a reduction in anxiety symptoms within 6 weeks of use of the SSRI sertraline [13]. It is also thought that people with anxiety may be more prone to adverse effects of SSRIs, particularly increased restlessness and initial worsening of symptoms [12]. Prescribing guidelines therefore suggest that for most anxiety disorders, SSRIs should be started at half the normal starting dose and titrated upwards up to the maximum tolerated dose [14]. It is understood that response is generally observed within 6 weeks and continues to increase over time [15]. The optimal duration of SSRI treatment for anxiety disorders is somewhat unclear, but guidelines suggest that treatment should be continued for at least 6 to 12 months beyond the initial successful response [11, 16]. It is recommended that patients on SSRIs should be assessed for the emergence of restlessness, increased anxiety and emergence of suicidal ideation [12].

Despite being frequently prescribed to autistic adults [17], the effectiveness and adverse effect profile of SSRIs in this population are not well understood. It has been suggested that these may not be identical to those in the non-autistic population [10]. SSRIs are thought to act initially via increased levels of available neurotransmitter serotonin (5-hydroxytryptamine, 5HT) in the synaptic cleft. The 5HT system is considered important in autism [18] and elevated 5HT in whole blood and platelets [19], and alterations in the developmental trajectory of brain 5HT synthesis activity [20] in autistic individuals have been reported. It has been suggested that increased 5HT uptake or storage in the presynaptic neuron could lead to decreased synaptic 5HT in autistic individuals [18]. This may underpin greater levels of anxiety, potential for benefits of SSRIs and also potential for greater sensitivity to adverse effects via an increase in peripheral serotonin levels in this population.

There is clinical equipoise in relation to SSRI use for anxiety symptoms in autistic adults [21]. To our knowledge, there have been three small RCTs of SSRIs in autistic adults to date [two for fluoxetine with *n* = 6 [20] and *n* = 37 [22] participants respectively, and one for fluvoxamine, *n* = 30 [23]] all with a focus on repetitive behaviours in autistic people. Recent systematic reviews highlighted the absence of mental health outcome data collection in RCTs involving SSRIs in autistic adults [8, 24]. The British Association for Psychopharmacology consensus guidelines for autism conclude that there is insufficient information regarding the effectiveness or side effect profile of SSRIs in the treatment of anxiety in the autistic population, calling for large-scale trials with adequate follow-up [21].

Research on interventions to help mental health problems and specifically anxiety was amongst the top priorities for research identified by the autism community, clinicians, researchers and other stakeholders in a national UK priority-setting exercise carried out by the James Lind Alliance and Autistica [25]. As SSRIs are widely prescribed to autistic adults without adequate evidence for effectiveness or understanding of adverse effects, it is essential that we get a better understanding of this topic. Such research has the potential to improve evidence-based care and lead to improvements in mental health and quality of life of the autistic population. The STRATA trial was designed in response to a joint commissioned call for a substantive RCT on this topic by the UK National Institute of Health and Care Research (NIHR) and the Australian National Health and Medical Research Council (NHMRC).

### Objectives {7}

STRATA aims to determine the effectiveness and cost-effectiveness of the SSRI sertraline in reducing anxiety and improving the quality of life in adults with an autism diagnosis compared with placebo and to quantify any adverse effects.

Primary objective: To determine the difference in Generalised Anxiety Disorder Assessment (GAD-7) [26] anxiety scores at 16 weeks between adults with an autism diagnosis treated with sertraline and those treated with a placebo.

Secondary objectives:

i) To describe the adverse effects reported by adults with a diagnosis of autism treated with sertraline versus those treated with a placebo over 52 weeks.
ii) To determine the effect of up to 52 weeks of treatment with sertraline versus placebo on:
  - GAD-7 score and proportionate reduction in GAD-7 scores including response (50% reduction in GAD-7 scores)
  - Patient-reported effect of medication on symptoms
  - Social anxiety
  - Obsessive–compulsive symptoms
  - Panic attacks
  - Repetitive behaviours
  - Meltdowns
  - Depressive symptoms
  - Composite anxiety and depressive symptoms
  - Functioning and disability
  - Quality of life
  - Carer burden *(*data collected in a Carer Sub-Study*)*
iii) To measure adherence to the study medication.
iv) To determine the cost-effectiveness of sertraline treatment for anxiety in adults with an autism diagnosis within an embedded economic evaluation.
v) To explore participants’ acceptability, experiences of, and adherence to, study processes and treatment within an embedded qualitative study.

### Trial design {8}

**Fig. 1:**
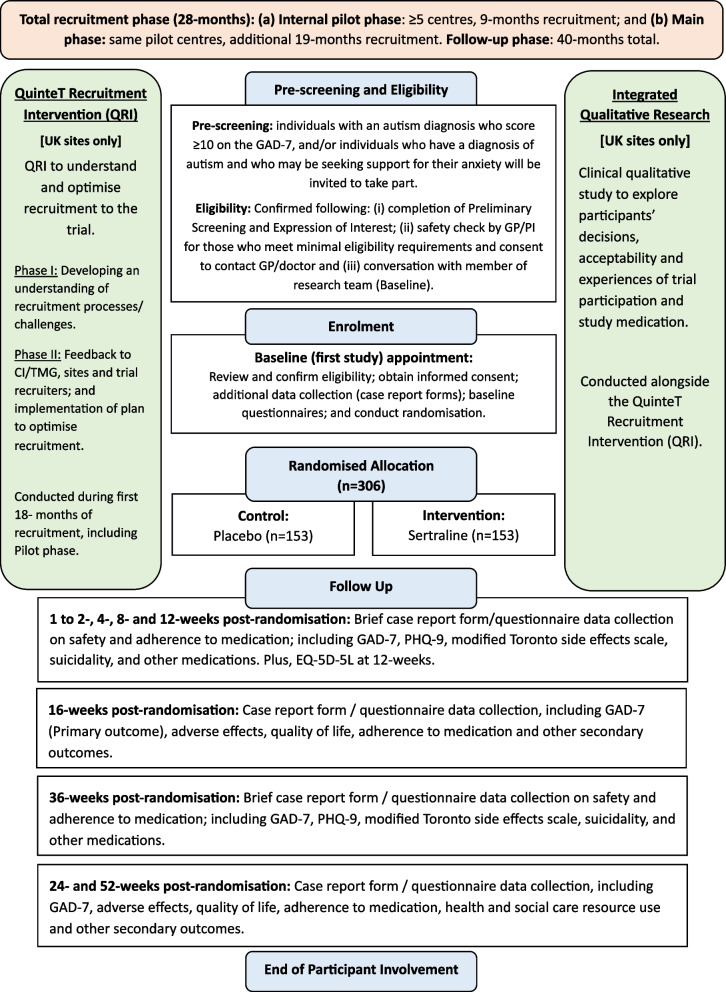
STRATA trial flowchart

STRATA is a two-parallel group, multi-centre, pragmatic, double-blinded RCT to examine whether sertraline is superior to placebo in reducing anxiety in adults with a diagnosis of autism (see Fig. 1, trial flowchart). Participants will be randomised in a 1:1 ratio to either sertraline or placebo with flexible titration from 25 mg to up to 200 mg and followed up for 52 weeks post-randomisation. A QuinteT Recruitment Intervention (QRI) [27] and qualitative research are embedded within STRATA to optimise recruitment and to understand the experiences of participation and the study medication. An economic evaluation will also be carried out (details provided in the “Methods for additional analyses (e.g. subgroup analyses) {20b}” section).

The trial was designed with an internal pilot aimed at demonstrating adequate recruitment with pre-specified progression criteria based on recruitment and site set up by the first 9 months of the recruitment period (see Table 1).

**Table 1:** STRATA Internal Pilot: Stop/Amend/Go criteria after first 9 months of recruitment

	**Participants recruited by end of pilot period**	**Anticipated action**
Go (Green)	55–78 participants (≥ 70% of expected)	Continue—Trial Management Group (TMG) will monitor recruitment rates closely
Amend (Amber)	39–54 participants (50–69% of expected) and recruitment not commenced in all 5 centres	Identify remediable factors, discuss with TMG and Trial Steering Committee. Submit recovery plan to funder (NIHR HTA)
Stop (Red)	0–38 participants (< 50% of expected) and recruitment not commenced in all 5 centres	Stop the trial, unless there is a strong case that unanticipated remediable factors have been identified and can be addressed after further discussion with the funder

## Methods: participants, interventions and outcomes

### Study setting {9}

This trial will be delivered through secondary care community autism services in four regional centres in the UK and one centre in Australia. The trial centres include (1) South West England; (2) Surrey, Hampshire and Portsmouth; (3) East of England; (4) East Midlands; and (5) Western Australia. Within each centre, there can be several recruiting sites which may recruit participants through patient lists, cohorts/registries, patient identification centres (PICs), general practitioner (GP) or self-referrals. A list of study sites can be found on the study website [28].

### Eligibility criteria {10}

Inclusion criteria:

- Adults aged ≥ 18 years.
- A diagnosis of autism made by a specialist including individuals with a co-occurring mild intellectual disability (ID). Autism diagnostic terms may include autism/autistic spectrum disorder or other variations, Asperger syndrome/disorder or pervasive developmental disorder.
- Anxiety as measured by GAD-7 score ≥ 10 at screening.

Exclusion criteria:

- Prescribed and regularly using a serotonergic antidepressant/anxiolytic at antidepressant doses in the preceding 8 weeks; these include SSRI and non-SSRI antidepressants including tricyclic antidepressants. Potential participants who are prescribed low (i.e. non-antidepressant) doses of these medications for other indications (e.g. neuropathic pain) or those who had no such medication for the majority of the preceding 8 weeks (e.g. tried for a few days before stopping) may be considered eligible where the site Principal Investigator (PI) confirms this is consistent with usual clinical practice. Individuals regularly using these medications wishing to participate could do so after a washout period of 8 weeks.
- Prescribed an irreversible monoamine oxidase inhibitor (phenelzine, isocarboxazid or tranylcympromine) or pimozide in the preceding 8 weeks.
- Diagnosis of moderate-severe intellectual disability (ID). People who have borderline to mild ID will be eligible. For the purpose of this study, a person with known ID will be considered as having a mild ID if they are able to provide written informed consent and have the ability to understand and answer the study questionnaires with the help of reasonable adjustments, if necessary.
- Inability to provide informed consent and complete study assessments/questionnaires.
- Currently valid diagnosis of bipolar disorder, manic or hypomanic episodes or psychosis. Individuals with historical diagnoses where there is clinical consensus or strong suspicion that these diagnoses are no longer valid (e.g. presentations historically labelled as mania/psychosis now considered to be explained by autism) may be considered eligible based on the discretion of the site PI.
- Currently uncontrolled epilepsy.
- Known current alcohol or drug use problem (i.e. if recorded in patient/medical notes and the GP/PI considers it unsafe to co-prescribe sertraline).
- Known allergies to sertraline or placebo/excipients.
- Currently enrolled in another RCT.
- Women who are pregnant, are planning pregnancy during the trial period or are breastfeeding.
- History of severe liver impairment.
- Bleeding disorders such as haemophilia, Christmas disease and von Willebrand’s disease, as well as those with past medical history of bleeding gastric or duodenal ulcers or other significant bleeding disorders.
- History of Long QT syndrome or Torsade de Pointes.
- Swallowing difficulties or inability to take medication in capsule form.
- Currently using St. John’s Wort.

### Who will take informed consent? {26a}

Delegated clinical research staff at sites who are trained on the STRATA protocol and procedures and have relevant experience including Good Clinical Practice training will be able to receive informed consent from potential participants. Potential participants will be sent a copy of the Participant Information Leaflet (PIL) with any initial invitation, and again following their expression of interest. During the baseline discussion, the researcher will go through the information in the PIL and check that individuals are fully informed about the study by asking them to summarise their understanding of what participating in the study will involve, enquire about the voluntary nature of their involvement and ask what will happen if they no longer wish to take part. This will enable the researcher to check that they have understood and retained key aspects of the information provided about the study and are aware of the voluntary nature of their involvement and their right to withdraw. To enable remote delivery of the trial, the default way of capturing consent will be via a Medicines and Healthcare products Regulatory Agency (MHRA) (UK) and NHMRC & Therapeutic Goods Administration (TGA) (Australia) compliant online eConsent form and process. An approved paper equivalent will be used where eConsent is not feasible.

Carers of STRATA participants will be recruited in parallel to explore carer burden in a nested sub-study. For the purposes of this study, a carer is a paid or unpaid individual or family member who knows the participant well, and helps them with tasks (e.g. with daily living tasks). If a participant confirms that they have a carer, the researcher will provide the participant with a STRATA Carer Study Information Pack, which will include an invitation letter, study information, consent form and questionnaire. The participant will be asked to forward this pack to their carer at the earliest opportunity, on behalf of the research team. The carer is enrolled in the sub-study if they complete and return the consent form and baseline questionnaire.

### Additional consent provisions for collection and use of participant data and biological specimens {26b}

Within the consent process for the trial, consent will also be sought for future re-contact and sharing of anonymised participant data for other ethically approved studies. Consent for linkage to central NHS records (e.g. NHS Digital linked data and equivalents for UK-patients, or equivalent electronic health records for patients in Western Australia) will also be sought, as a possible mechanism for future longer-term follow-up.

No biological specimens will be collected within this trial.

## Interventions

### Explanation for the choice of comparators {6b}

The comparator in this study is a matched placebo since the primary aim is to assess whether sertraline is effective for the treatment of anxiety in adults with an autism diagnosis.

### Intervention description {11a}

The active investigational medicinal products (IMP) are over-encapsulated 25 mg or 50 mg sertraline tablets with a back fill of microcrystalline cellulose powder. The placebo will be manufactured in the form of matched capsules filled with microcrystalline cellulose powder which look identical to the active medication capsules.

All participants will receive a daily dose of 25 mg sertraline or matched placebo for 2 weeks followed by 2 × 25 mg for 4 weeks. Following this initiation period, the medication will be dispensed in 50 mg capsules, and depending upon tolerability, the dose will be flexibly increased by 50 mg every 4 weeks to reach the optimal dose. The dose will only be increased if the participant is tolerating it and agrees to try an increased dose, and the prescribing clinician is satisfied that it is appropriate to do so based on the participant’s discussion with the study researcher and responses to the safety check questionnaire. The dose may go up to a maximum of 200 mg by week 14, although for many participants the optimal dose may be lower (e.g. 25 mg, 50 mg, 100 mg or 150 mg) and reached before this time. Participants will take this optimal dose for up to 52 weeks post-randomisation. The same dosing schedule will be applied to participants in both groups with dummy dose titration being applied to the placebo group (the participants, researchers and the prescribers will be blinded as described below).

### Criteria for discontinuing or modifying allocated interventions {11b}

Patients will be enrolled with the understanding that the study involves being prescribed the study medication for up to 52 weeks but that they will be supported to discontinue the medication at any time they wish. During the period of dose titration, participants will have four safety checks (at 1 to 2, 4, 8 and 12 weeks post-randomisation) for brief data collection on adverse effects, mood, anxiety and suicidal ideation and a discussion with the researcher on how they are getting on with the study medication. Decisions on whether to increase, decrease or maintain a given dose of the medication will be the responsibility of the PI/delegated prescriber but will be made in a collaborative way, taking into account the views of the patient and the information collected at the safety check appointment. A similar brief safety check will also take place at 36 weeks post-randomisation. Participants can also contact the study team at any point should they experience adverse effects or wish to discuss changing the dose or discontinuing the medication.

If the participant discontinues the medication because of unacceptable side effects or any other reason, they will be advised to follow the downward dosing regimen (reduction of medication by 50 mg per week) before being returned to standard care. They will be encouraged to remain enrolled in the trial, unless they explicitly withdraw, and complete further questionnaires as per protocol. Once trial medication is discontinued, participants may not resume trial treatment but as this is a pragmatic trial, they may be prescribed any other medication, including sertraline by their clinician. Information on any new medication (or psychological therapy) will be collected at all follow-up time points.

After 52 weeks post-randomisation, participants who are still on the study medication will be asked to complete a downward titration for a period of up to 4 weeks (which involves the reduction of medication by 50 mg per week) before being returned to standard care. Those who wish to continue medication post-trial will be supported to make an appointment with their usual clinician (GP or secondary care specialist), who will be informed of their trial allocation by the study pharmacy in order to discuss and make further treatment decisions.

### Strategies to improve adherence to interventions {11c}

Information about taking the study medication, including possible ways to aid adherence will be included in the STRATA Medication Instructions provided at enrolment. However, considering the pragmatic nature of this trial, no additional measures to improve poor treatment adherence amongst participants will be implemented as these may not reflect real-life practice and act as an additional intervention.

In the case of persistent non-adherence to treatment, a pragmatic clinical decision will be made. For example, if a participant is on a higher dose of study medication and reports persistent non-adherence, it may be advisable to withdraw them from the trial treatment (but not from completing future study questionnaires). Such decisions will be made by the prescribing PI in consultation with the Chief Investigator (CI) on a case-by-case basis.

**Fig. 2:**
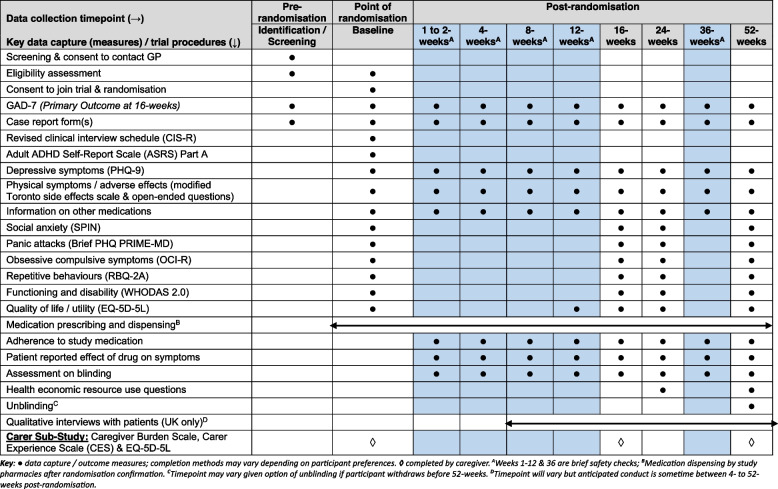
Schedule of data collection

Medication adherence will be assessed using questions adapted from the GENPOD [29] and PANDA [13] trials about adherence to medications (see Fig. 2 for timepoints).

### Relevant concomitant care permitted or prohibited during the trial {11d}

STRATA is a pragmatic trial and usual care can continue without restriction, including referrals to psychological therapies. GPs/clinicians can also prescribe other medication as necessary but will be asked to exercise caution in case they plan to prescribe drugs that may interact with sertraline. Information on other drug or psychological treatments will be collected at baseline and at all follow-up time points. A list of contra-indicated medications and/or cautions can be found in the supporting Summary of Product Characteristics (SmPC) available on the study website [30].

### Provisions for post-trial care {30}

After 52 weeks post-randomisation, participants will be returned to standard care following a down-titration period of up to 4 weeks. Continuation of the treatment following the end of the down-titration is the responsibility of the participant’s usual clinician (GP or secondary care specialist). This information will be discussed with the participant at enrolment and in the final study appointment. The participants’ usual clinician will be informed of their trial allocation by the pharmacy after 52 weeks post-randomisation and this information can be used by the clinician in discussing further care with the patient.

### Outcomes {12}

The primary and secondary outcomes are summarised in Table 2. Outcomes will be measured at 16, 24 and 52 weeks post-randomisation, with brief data collection on safety and medication adherence at 1 to 2, 4, 8, 12 and 36 weeks post-randomisation [see Fig. 2 for details of trial assessments and timepoints]. All outcome measures alongside procedures for the study were decided following extensive consultation with the autistic advisory group with feedback and discussion on clarity, acceptability, relevance and potential burden to potential participants.

**Table 2:** Summary of primary and secondary outcomes and measures

Outcome	Tool / method
**Primary Outcome**	
To determine the difference in Generalised Anxiety Disorder Assessment (GAD-7) anxiety scores at 16 weeks between adults with a diagnosis of autism treated with sertraline and those treated with a placebo	GAD-7 anxiety score
**Secondary Outcome**	
**i).** To describe the adverse effects reported by adults with a diagnosis of autism treated with sertraline versus those treated with a placebo over 52 weeks	Modified Toronto side effects scale and open-ended questions (including suicidality item)
**ii).** To determine the effect of up to 52 weeks of treatment with sertraline versus placebo on:	-
**a).** GAD -7 score and proportionate change in GAD-7 scores including response (50% reduction in GAD-7 scores)	GAD-7
**b).** Patient-reported effect of medication on symptoms	Study-specific questionnaire
**c).** Social anxiety	Social Phobia Inventory (SPIN)
**d).** Obsessive-compulsive symptoms	Obsessive Compulsory Inventory Revised (OCI-R)
**e).** Panic attacks	Brief Patient Health Questionnaire (PHQ) from Primary Care Evaluation of Mental Disorders (PRIME-MD)
**f).** Repetitive behaviours	Adult Repetitive Behaviours Questionnaire-2 (RBQ-2A)
**g).** Meltdowns	Single item ‘had a meltdown’ added to GAD-7 scale
**h).** Depressive symptoms	Patient Health Questionnaire-9 (PHQ-9)
**i).** Composite anxiety and depressive symptoms	Sum of PHQ-9 and GAD-7 Scores
**j).** Functioning and disability	World Health Organization Disability Assessment Schedule 2.0 (WHODAS 2.0)
**k).** Quality of life	EQ-5D-5L questionnaire
**l).** Carer burden and quality of life	Caregiver Burden Scale, Carer Experience Scale (CES) and EQ-5D-5L questionnaire
a). To measure adherence to the study medication	Questionnaire (adapted from GENPOD and PANDA trials)
b). To determine the cost-effectiveness of sertraline treatment for anxiety in adults with a diagnosis of autism	EQ-5D-5L (to calculate QALYs) and study-specific patient resource use questionnaire
c). To explore participants’ acceptability, experiences of, and adherence to study processes and treatment	Qualitative interviews with participants

#### Primary outcome data

The primary outcome is GAD-7 [26] score at 16 weeks post-randomisation as a continuous outcome. The GAD-7 is a 7-item patient-reported anxiety measure and is a key outcome measure used in the UK NHS Talking Therapy Services [previously called Improving Access to Psychological Therapies (IAPT) services] and can be easily used in primary care, where management of anxiety in adults with a diagnosis of autism is likely to happen. It was used in a feasibility RCT of guided self-help for depression in autistic adults [31], and our autistic advisory group members found the questions easy to follow and quick to complete. Using the threshold score of 10, GAD-7 has 89% sensitivity and 82% specificity for generalised anxiety disorder and is also good at screening panic disorder, social anxiety disorder and posttraumatic stress disorder (PTSD) [32]. GAD-7 will be measured at baseline, 1 to 2, 4, 8, 12, 16, 24, 36 and 52 weeks post-randomisation.

#### Secondary outcome data

As a secondary outcome based on GAD-7, we will also assess proportionate change in GAD-7 scores including defining a binary ‘response’ variable with 50% reduction in GAD-7 score compared to baseline. Understanding adverse effects related to sertraline treatment is an important secondary outcome in this trial. We will measure adverse effects using a list of items based on the Toronto side effects scale which has been designed to measure antidepressant adverse effects and has been used in several large trials of antidepressants [13, 33]. We will supplement this with open-ended questions, including suicidality items and the Patient Health Questionnaire (PHQ-9) [34] as used in a previous feasibility RCT involving autistic adults [31]. The GAD-7 scores at follow-up will indicate any initial worsening of anxiety, which may also be an adverse effect of sertraline. Adverse effects data will be collected at baseline, 1 to 2, 4, 8, 12, 16, 24, 36 and 52 weeks post-randomisation.

We will also include different facets of anxiety (e.g. social anxiety, obsessive-compulsive symptoms including those important in autism but not adequately captured in general anxiety screens). These include social anxiety (SPIN) [35]; obsessive-compulsive symptoms (OCI-R) [36]; panic attacks (Brief PHQ from PRIME-MD) [37]; and repetitive behaviours (RBQ-2A) [38]. These data will be collected at baseline and 16, 24 and 52 weeks post-randomisation.

Other secondary outcomes will include depressive symptoms (PHQ-9) [34], and questions about adherence to medications, functioning and disability (World Health Organization Disability Assessment, WHODAS) [39] and Quality of Life/Utility (EQ-5D-5L) [40] are summarised in Table 1 and Fig. 2.

Carer burden and quality of life (Caregiver burden scale [41], Carers Experience Scale (CES) [42] and EQ-5D-5L [40]) will be measured at baseline and 16 and 52 weeks post-randomisation and collected within the carer sub-study.

The experiences of participants in taking the study medication and taking part in the study (including reasons for discontinuing the medication or deciding to stop taking part in the study) will be assessed by 1:1 semi-structured interviews which will be conducted at various stages of follow-up.

### Participant timeline {13}

See Fig. 2 for the participant timeline for the trial.

### Sample size {14}

The sample size calculations are based on the literature regarding the primary outcome (GAD-7) and experience in the ADEPT study of autistic adults [31]. A reduction of 2 to 3 points in the total GAD-7 score has been reported as a clinically important change [43], and based on this, this trial is designed to detect a difference of 2.2 points on the GAD-7 between treatment arms at 16 weeks. The results from the ADEPT study suggest a standard deviation (SD) in GAD-7 scores of 5.7 [31] meaning the target difference equates to approximately 0.39 SD. Based on this, the study aims to recruit 306 participants, in which estimating 20% attrition at 16 weeks and a correlation of 0.37 between the baseline and 16-week GAD-7 scores [31] will yield 90% power (alpha = 0.05) to estimate a mean difference of 2.2 points in GAD-7 scores between groups as randomised. Table 3 summarises the differences in GAD-7 scores between treatments that could be detected with at least 80% power by randomising 306 patients to the study assuming alpha = 0.05.

**Table 3:** Difference in GAD-7 scores between treatment arms at 16 weeks based on assumptions for the power calculations made in this study

	**Difference in GAD-7 scores between treatment arms at 16 weeks**						
	**2.5**	**2.4**	**2.3**	**2.2**	**2.1**	**2.0**	**1.9**
Power	95.8%	94.3%	92.4%	90.1%	87.2%	83.9%	80%

No a priori sample size calculation was conducted for the carer sub-study which will include all eligible and consenting carers of randomised STRATA participants. Should all 306 STRATA participants have a recruited carer participating in the study, the study will have 90% power (alpha = 0.05) to detect a 0.4SD difference in the carer burden scale assuming 30% attrition. Should half of STRATA participants have a recruited carer participating in the study, the study will have 90% power (alpha = 0.5) to detect a 0.6SD difference, assuming 30% attrition.

### Recruitment {15}

Since very little is known about recruiting autistic adults to RCTs, the possibility of recruitment difficulties was anticipated at the funding application stage. The advisory group were consulted for their expertise and signposting to potential networks as avenues for participant recruitment. In preparation for this study, qualitative work with autistic adults was carried out to understand the acceptability of RCTs and their processes [44], and to understand how the COVID-19 pandemic might influence the participation of autistic people in future research [45]. Funding was also awarded to embed a QuinteT Recruitment Intervention (QRI) to understand and optimise the recruitment to trial process [27] and integrate qualitative research to explore participants’ decisions, acceptability and experiences of study participation and medication. The QRI will be implemented in STRATA’s UK centres/sites to optimise recruitment and informed consent with lessons learnt shared across all sites, including the Australia centre.

The QRI will be implemented in two phases:

QRI—phase 1 will investigate the recruitment process and how it operates within centres/sites, building up a comprehensive understanding of recruitment challenges that arise during the internal pilot phase and beyond. A multi-faceted, flexible approach will be adopted, using one or more of the following methods: mapping patient eligibility and recruitment pathways/numbers across sites, recording of recruitment appointments, attendance at trial management group (TMG) and investigator meetings, review of study documentation and in-depth interviews with (a) members of the TMG and those closely involved in the design, management, leadership and co-ordination of the trial; (b) health professionals and researchers who are involved in trial recruitment (trial recruiters); and (c) patients who have been approached to take part in the trial.

Interviews with TMG members and trial recruiters will investigate their perspectives on the RCT, including the design and the evidence on which it is based, and views and experiences of recruiting patients at site. Interviews with participants who have been approached about the study will explore views on the study information, understanding and acceptance of trial processes (including, for example, randomisation, placebo, blinding) and reasons underlying decisions to accept or decline trial participation. Participants will be purposefully selected, to build a maximum variation sample on the basis of age, gender, study site and final decision about trial participation (i.e. accept/decline). Numbers will be dependent on whether sufficient understanding has been gained on issues raised (data saturation).

QRI—phase 2: Development and implementation of recruitment strategies: findings from phase 1 of the QRI will be presented to the CI/TMG, identifying factors that appear to be hindering recruitment and action plans will be developed and implemented.

The QRI will be undertaken in an iterative and cyclical manner, continuing throughout the early stages of recruitment.

Alongside the QRI, semi-structured qualitative interviews will be conducted with trial participants to explore views, expectations, experiences and acceptability of the study processes and treatment, along with issues around adherence. The final sample size for the interviews will be driven by data saturation. Participants will be purposefully selected to ensure maximum variation in terms of age, gender, recruiting centre/site and engagement with the medication (e.g. withdrawal).

Interviews will be undertaken at the participant’s preferred location (e.g. home, study site) and through their preferred method (e.g. video-conferencing, face-to-face), assuming they are in a suitably private and quiet setting. All interviews will be audio-recorded with consent on an encrypted device (or recorded via an alternative secure device/mechanism, including approved video-conferencing tools) and a topic guide will be used to ensure the key areas stated above are covered but with flexibility to let the participants raise issues of importance to them.

Qualitative interviews and recruitment consultations, along with screening logs and study documentation, will be subject to simple counts, content and thematic analyses.

## Assignment of interventions: allocation

### Sequence generation {16a}

The randomisation sequence will be generated by Sealed Envelope™ [46]. Randomisation will be stratified by centre, with minimisation to ensure balance in baseline GAD-7 score (< 15 and ≥ 15), gender (male, female, other), age (18–34, 35–49 and ≥ 50), presence of intellectual disability (yes/no) and previous medication use for anxiety or depression (yes/no). Patients will be randomised to one of two treatment groups in a 1:1 ratio to either sertraline (intervention arm) or placebo (control arm).

### Concealment mechanism {16b}

The web-based Sealed Envelope randomisation system ensures allocation concealment as the randomised code is only released once the patient is enrolled as described below.

### Implementation {16c}

The local PI (or authorised delegate) will sign into the Sealed Envelope secure online randomisation system and enter the individual’s unique study identification number and necessary minimisation variables. They will then receive the code that allocates the participant to the study treatment, and this code will be recorded on the study-specific prescription sent to the study pharmacy. The PI, researchers and the participant will remain blinded as to which treatment group this code refers to. The study pharmacies in the UK and Australia respectively will hold the unblinded randomisation code to dispense the allocated treatment to the patient.

## Assignment of interventions: blinding

### Who will be blinded {17a}

The study clinicians/investigators, researchers/research team, site staff and participants will be blinded to the allocation of treatment group. The trial pharmacies in the UK and Australia will be unblinded and dispense the study medication based on the randomisation code which will be recorded on the study prescriptions made by the prescriber. Two statisticians based at the University of Bristol (UoB) will support this trial. The senior statistician will be blinded throughout. The second statistician will perform all disaggregated analyses according to a pre-specified statistical analysis plan (SAP) and will attend closed Data Monitoring and Ethics Committee (DMEC) meetings as required. In addition, the health economist(s) will be blinded when cleaning data and preparing the analysis plan, but unblinded when conducting the analysis. The database manager at the Bristol Trials Centre (BTC) will also have access to unblinded data.

### Procedure for unblinding if needed {17b}

Unblinding of the research team: Treatment codes will only be released to the investigative team once written confirmation has been received that the trial database has been locked. The pharmacies in the UK and Australia will then send the relevant central research team (UK or Australia) a list of all participants and their treatment allocation. Any incidents of unblinding before the trial database has been locked will be recorded.

Unblinding of participants: Participants will be given the option of unblinding after their involvement with the study has ended, so that they can contact their usual care provider and seek to continue/initiate sertraline or alternative treatment if they wish to. Alternatively, those who withdraw from taking the medication or the study will be given the option of unblinding at the time of medication withdrawal or the 16-week primary outcome—whichever comes later. In such cases, the pharmacy will be instructed to send the treatment allocation to the participant’s GPs (or equivalent healthcare professionals in Australia), who may then discuss the participant’s allocation with them and discuss further care as necessary. The participant and their GP will be expressly instructed to keep the research team blinded from this information in any future communications. This unblinding strategy was developed based on feedback from the autistic advisory group as being most acceptable to potential participants.

In the event of a medical emergency, the participant’s treating doctor can contact the relevant central pharmacy who will hold the treatment allocation and will be available 24/7. Any instances of emergency unblinding will be recorded, and where possible, all efforts will be made to continue to keep the research team and prescribers blinded.

## Data collection and management

### Plans for assessment and collection of outcomes {18a}

All trial timelines for data collection are summarised in Fig. 2.

Participants in the trial will undergo the following: identification and screening contact; consent and randomisation (enrolment); brief contact (safety checks) at 1 to 2, 4, 8, 12 and 36 weeks post-randomisation; and assessments at baseline (0 weeks) and follow-up at 16 (primary outcome), 24 and 52 weeks post-randomisation.

Potentially eligible participants can be identified from a number of possible pathways including clinical appointments/lists, research registers/cohorts and self-referral. All potential participants are directed to the study website which contains information about the study including the Participant Information Leaflet, and requested to complete the preliminary online screening questionnaire and expression of interest (EOI) form (or request/complete a paper copy). This asks a short series of questions based on broad inclusion/exclusion criteria and contact details and seeks permission to contact the individual’s GP/doctor to complete patient safety checks. Individuals for whom the study is unsuitable are informed at this stage. Those who are potentially eligible are informed that their GP has been contacted to check if it is safe for them to be prescribed the study medication and take part in this study. The patient’s GP is sent a safety check form to complete or they can send summary record information which can be used by the site PI to complete the form. Once the GP safety check has been completed, individuals for whom the study is considered suitable, and are interested in taking part, will be invited to attend a baseline appointment which can take place by video call, in-person or via telephone depending upon the participant preference.

At the baseline appointment, the researcher will discuss the study again with the potential participant, confirm eligibility, obtain informed consent and complete outstanding baseline data collection. The local prescriber (PI/delegated clinician, or non-medical clinician such as an advanced nurse practitioner or clinical nurse specialist) will review this information. If they are satisfied that eligibility criteria have been met and valid consent has been received, they will provide approval, randomise the participant to the study and commence prescription of the study medication and subsequent participant follow-ups are arranged. If the prescriber believes that eligibility criteria have not been met and that prescriptions cannot commence, the researcher will notify the individual that the study is not suitable for them.

Following baseline assessment, follow-up assessments will take place at 16 (primary outcome), 24 and 52 weeks post-randomisation. Brief data collection will also take place during the safety check assessments at 1–2, 4, 8, 12 and 36 weeks post-randomisation.

Follow-up assessments: Participants will be asked to complete a follow-up questionnaire at 16, 24 and 52 weeks post-randomisation; each questionnaire will contain Patient-Reported Outcome Measures (PROMs) and additional data collection as presented in Fig. 2. Participants will be asked to complete the questionnaires online and will receive a secure online link at the appropriate timepoints. Alternative methods preferred by the participant will be facilitated where feasible (e.g. by video call, postal hard copy, face-to-face or telephone). If the participant requires assistance to complete the questionnaires, the research team will try and make all reasonable adjustments requested by the participant to facilitate this. A carer/family member or friend can provide support, but they will be advised not to answer any questions on behalf of the participant.

Brief contact (safety checks): At 1 to 2, 4, 8, 12 and 36 weeks post-randomisation, the local researcher will contact the participant to conduct a brief safety check to assess safety (adverse effects), medication dose titration and anxiety and depressive symptoms, including suicidality. The assessment will be carried out via online questionnaire completion and subsequent discussion with the researcher to adequately inform dose titration. The discussion will include how participants are getting on with the medication including any adverse effects, whether they are happy to continue taking the study medication and their view on whether they would like to stay on the same dose or try an increased dose or would like a reduced dose of the study medication. This information will be considered by the prescribing clinician to make decisions on dose titration per protocol.

### Plans to promote participant retention and complete follow-up {18b}

The trial has been designed with extensive input from the STRATA Autistic Advisory Group and incorporates various suggestions to ensure the study is tailored to the needs of autistic adults, and its processes are acceptable to potential participants. Additionally, we will take active measures to minimise the loss of participants from the trial in line with ethical and regulatory approval. This may include, for example, the ability to complete questionnaires via their preferred method (e.g. online/ post/ telephone/ video call/ face-to-face); reminders to participants according to individual contact preference; obtaining back-up ‘best contact’ addresses (including carer/ other family member, where applicable); contacting their GP (practice) to check their contact details on record are still valid [47]; and using vouchers as retention incentives [48]. In addition, we may access centrally-held health data, for example via the NHS Strategic Tracing Service in England and Wales, and WebPAS in Western Australia, to find new addresses.

### Data management {19}

Data will be entered directly at the point of collection onto the bespoke study database built using REDCap [49]. REDCap is a secure, web-based electronic data capture (EDC) system designed for the collection of research data. The system has been developed and supported by Vanderbilt University. Bristol Trials Centre has set up its own infrastructure so that all systems are hosted at and supported by the University of Bristol (UoB). The electronic data capture includes completed study questionnaires via secure emailed links to participants, e-consent, e-prescribing and automated reminders. Data obtained by paper will be entered into the database by the research team.

Administrative and clinical study data will be stored in separate REDCap instances. The administrative data will be kept in a secure database that is only accessible from within the UoB firewall. All users will require UoB accounts to access it via secure Virtual Private Network (VPN) or secure remote desktop. The clinical data will be stored on a separate server to the administrative data. Anonymised clinical data is linked by a study participant identification number.

All research data will be retained securely during the conduct of the trial. Data will be retained for at least 5 years after the end of the trial (15-year requirement for Australia) and, at the end of the archiving period, will be destroyed by confidential means with the exception of a final anonymised dataset.

### Confidentiality {27}

The University of Bristol (UK) is the data controller. All personal identifiable and clinical data will be held at the University of Bristol and will conform to the University of Bristol Data Security Policy and in Compliance with General Data Protection Regulation (GDPR) as it applies in the UK, tailored by the Data Protection Act 2018, which in turn also comply with the Australian Privacy Principles (APP) set out in the Australian Privacy Act 1988.

### Plans for collection, laboratory evaluation and storage of biological specimens for genetic or molecular analysis in this trial/future use {33}

There are no plans to collect any biological specimens within this trial.

## Statistical methods

### Statistical methods for primary and secondary outcomes {20a}

All analyses and reporting will be in line with CONSORT (Consolidated Standards of Reporting Trials) guidelines [50]. Primary analyses will be based on the intention-to-treat (ITT) principle, analysing participants in the groups to which they were randomised. A full statistical analysis plan (SAP) will be developed and agreed upon by the Trial Steering Committee (TSC) prior to undertaking analyses.

Descriptive statistics will be used to determine whether there are imbalances at baseline between treatment groups. Should meaningful differences be observed, sensitivity analyses will be performed adjusting for this imbalance. Continuous measures will be presented as means and SDs or medians, inter-quartile ranges and ranges depending on their distribution. Categorical data will be presented as frequencies and proportions. Patient-reported outcome scores based on standardised questionnaires will be calculated based on the developers’ scoring manuals and missing erroneous items will be handled according to these manuals.

The primary analysis of the effectiveness of the primary outcome will use linear regression to estimate an adjusted difference in means comparing GAD-7 score at 16 weeks post-randomisation between groups, adjusted for baseline, centre, sex, presence of intellectual disability and previous use of SSRIs at baseline (stratification/minimisation variables).

Secondary analyses of the primary outcome will include a Complier Average Causal Effect (CACE) analysis [51] to investigate the efficacy of the intervention (based on treatment compliance status) for comparison with the ITT estimate of the offer of the intervention and a per-protocol analysis which will account for those patients taking treatments other than that which they were allocated to.

The effect of the intervention on the secondary outcomes collected at 16, 24, 36 and 52 weeks post-randomisation will also be examined using linear regression for continuous outcomes and logistic regression for binary outcomes adjusted for baseline values of the outcome being investigated and stratification/minimisation variables.

As it is possible that adherence to treatments will decrease over the 52-week follow-up, we will describe this at each timepoint by arm as well as the use of additional or alternative medications or other treatments. A repeated measures analysis using GAD-7 and other outcome data collected at multiple follow-up timepoints will be carried out to examine the effect of the intervention over 52 weeks.

All analyses of primary and secondary outcomes will adjust for baseline values of the outcome, stratification and minimisation variables. Sensitivity analyses of the primary outcome will adjust for any prognostic variables showing a marked imbalance at baseline (ascertained using descriptive statistics).

### Interim analyses {21b}

No interim analyses are planned.

### Methods for additional analyses (e.g. subgroup analyses) {20b}

A number of pre-defined subgroup analyses will be carried out to assess the difference in treatment effect on the primary outcome according to characteristics assessed at baseline. Characteristics of interest include whether the diagnosis of autism was made as an adult or child, the presence of mild ID, the presence of attention-deficit hyperactivity disorder (ADHD) features and severity of anxiety symptoms. Effect modification will be assessed by including an interaction term in the regression model and formal tests of interaction will be performed to test whether the treatment effect differs between these groups. As the study was not powered to detect such effects, results will be interpreted with caution.

Descriptive analyses of safety endpoints will be presented at each timepoint according to treatment received. No formal comparisons will be made between groups.

Analyses of the carer-burden sub-study will follow the principles of the effectiveness analysis. Descriptive statistics will be used to describe the baseline characteristics of carers participating in the sub-study as well as the randomised participants they are caring for. These results will be used to determine whether there are imbalances at baseline between treatment groups and suggest whether appropriate additional adjustments should be performed. Continuous measures will be presented as means and SDs or medians, inter-quartile ranges and ranges depending on their distribution. Categorical data will be presented as frequencies and proportions. The effect of the intervention on the carer burden scale, carers experience scale and EQ-5D-5L collected at 16 and 52 weeks post-randomisation will be examined using linear regression adjusting for baseline values, variables used in the randomisation and any variables found to be imbalanced at baseline.

#### Economic evaluation within STRATA

##### Aim

The aim of the economic evaluation in STRATA is to assess the cost-effectiveness of sertraline plus usual care compared with placebo plus usual care for the treatment of anxiety in autistic adults. Cost-effectiveness will be assessed from the perspective of the NHS and personal social services (PSS) in the UK and from a societal perspective, including productivity losses.

##### Outcomes

The primary outcome for the economic evaluation will be quality-adjusted life years derived from measurements recorded using the EQ-5D-5L health-related quality of life instrument [40] after 52 weeks of follow-up. Quality of life (via EQ-5D-5L) will be measured at baseline, 12, 16, 24 and 52 weeks of follow-up. Reported EQ-5D-5L health states will be valued using the method recommended by National Institute of Health and Care Excellence (NICE) at the time of analysis; the current position statement recommends the use of the Van Hout crosswalk [52]. A secondary outcome will be the GAD-7 anxiety score at 52 weeks post-randomisation.

##### Cost measurement

Resources used by participants (other than sertraline) will be tracked by means of a concise bespoke patient-reported questionnaire (electronic or paper as per participant preference) administered to each group at 24 and 52 weeks post-randomisation. The resource-use questionnaire will cover hospital admissions (including length of stay), outpatient appointments, emergency department visits, primary care appointments, home visits, social care contacts and medications. In addition, participants will be asked to report time off work, if applicable. As it may be difficult for participants to accurately identify whether a contact was associated with their anxiety, information on healthcare resources used for any reason will be requested. The resource-use questionnaire (RUQ) has been developed with input from our autistic advisory group. The RUQ will be piloted using data from the internal pilot and will be adapted for later participants if necessary. Effectiveness and resource-use data will be taken from the UK only; valuations will be assigned to recorded resources using the most recently available standard UK sources at the time of analysis, such as the Unit Costs of Health and Social Care for primary and community care [53], the NHS reference costs for secondary care contacts [54] and the British National Formulary for prescribed medication costs [55].

##### Analysis

The analysis will be guided by a pre-specified health economics analysis plan (HEAP) agreed with the TSC. The primary cost–utility analysis (CUA) will be conducted from the NHS and PSS perspective. The CUA will compare the difference in costs with the difference in quality-adjusted life years (QALYs) between the groups after 52 weeks of follow-up; both incremental cost-effectiveness ratio and incremental net monetary benefit statistics will be calculated. A cost–consequences analysis (CCA) will also be presented from a societal perspective. The CCA will relate the differences in costs (including health and social care costs, and productivity loss) and a range of outcomes (QALYs, GAD-7, secondary outcomes from the effectiveness analysis, and carer outcomes) for each arm over 52 weeks of follow-up. As the period of follow-up is 52 weeks (1 year) only, discounting of either costs or benefits is unnecessary. Sensitivity analyses will be conducted to assess the effect of assumptions made in the analysis and uncertainty in estimates of unit costs. Uncertainty in cost-effectiveness statistics arising from patient variability will be assessed by constructing cost-effectiveness acceptability curves and by deriving confidence intervals for the net monetary benefit statistic.

### Methods in analysis to handle protocol non-adherence and any statistical methods to handle missing data {20c}

The sensitivity of the primary analysis to the impact of missing data will be investigated. The data will first be explored before a decision is made on what approach to utilise. These include exploring the amount of missingness, differences between arms, variables associated with/predictive of missingness and if reported, reasons for missingness. The approach taken to handling missing data will then depend on the assumptions about the nature of the missingness deemed to be appropriate. For example, if an assumption of Missing At Random is deemed appropriate, then multiple imputation will be carried out and the primary analysis repeated using the imputed data.

### Plans to give access to the full protocol, participant-level data and statistical code {31c}

The full protocol is available in the Supplementary file 1. The final trial data set will be stored as restricted data on the data.bris research data repository. A data-sharing policy will be agreed with the Trial Management Group. Anonymised data can be requested by bona fide researchers following the submission of a proposal of intended use and after their host institution has signed a data access agreement. The analytic code for publications arising from the data can be requested from the corresponding author.

## Oversight and monitoring

### Composition of the coordinating centre and trial steering committee {5d}

The Sponsor will be responsible for overall oversight of the trial. The study is supervised by a Trial Management Group (TMG) consisting of applicants for the funding application and other relevant trial delivery staff. The TMG has responsibility for the day-to-day management of the trial and will report to the Trial Steering Committee (TSC). The TSC oversees the progress of the trial, Chaired by Professor Nick Freemantle (UCL), and comprises four other independent members including a patient and public involvement (PPI) representative and includes the Chief investigator as a non-voting member. Membership, responsibilities and reporting mechanisms of the TSC are formalised in a TSC charter. The TSC will make recommendations/key decisions during the trial to the TMG and minutes will be sent to the funder.

### Composition of the data monitoring committee, its role and reporting structure {21a}

An independent Data Monitoring and Ethics Committee (DMEC) monitors accumulating trial data for quality, completeness and patient safety and comprises an independent chair (Dr Louise Marston, UCL) and two other independent members. The DMEC will meet once prior to recruitment of the first participant and subsequently convene every 6–12 months, prior to the TSC meetings, to review any safety, data quality or ethical aspects that arise and report to the TSC. Responsibilities and reporting mechanisms are formalised in a DMEC charter. The Chief investigator, Trial Manager and the Senior Statistician will attend the open sessions of the DMEC. The second (unblinded) Statistician will attend both open and closed sessions.

### Adverse event reporting and harms {22}

Pharmacovigilance will be carried out in accordance with the requirements set out by Medicines for Human Use (Clinical Trials) Regulations 2004 and the Medicines for Human Use (Clinical Trials) (Amendment) (EU Exit) Regulations 2019 (UK), and Therapeutic Goods Administration (TGA) and the NHMRC Safety Monitoring and Reporting in Clinical Trials Involving Therapeutic Goods (Australia). This includes the terminology of adverse events (AE) and reactions and the assessment of seriousness, causality and expectedness of an event, in accordance with these regulations.

Most non-serious AEs that are related to sertraline (adverse reaction (AR)) will be expected reactions previously identified and described in the product characteristics. New mental health diagnoses/symptoms that are unrelated to the IMP may also occur. These events will be collected by participant self-report using the Modified Toronto side effects scale and open-ended questions (including suicidality item) in the study questionnaires from the time a signed and dated informed consent form is obtained until completion of the last trial-related procedure for each participant. Events not captured by the questionnaires will be recorded by the researcher. The central research team will prepare summary reports of all recorded non-serious AEs for discussion at relevant oversight meetings.

If an event is defined as ‘serious’ then within 24 h of becoming aware of a serious adverse event (SAE), the local research team will notify the Sponsor, the CI and the UK central research team. The local research team will provide information missing from the initial report within 5 working days of the initial report to the necessary bodies. Any change of condition or other follow-up information relating to a previously reported SAE will be reported on a separate trial SAE/SUSAR (Suspected Unexpected Serious Adverse Reaction) Follow-Up Report Form. All SAEs will be followed up until the event has resolved, or a final outcome has been reached. SUSARs will be further reported to the research ethics committee (REC), DMEC and MHRA within 7 days of the Sponsor being notified if fatal or life-threatening, or 15 days otherwise. All SAEs will be further reported to the DMEC on a quarterly basis.

The Western Australian research team will follow the same procedures as described above. In addition, the Western Australian research team will report SAEs for Australian-recruited participants to their Research Governance Office (RGO) within 72 h, in line with the NHMRC requirements.

The University of Bristol holds insurance to cover the University’s legal liability as Research Sponsor, in the event of harm to research participants arising from the management of the research by the University.

### Frequency and plans for auditing trial conduct {23}

The trial will be monitored and audited in accordance with the Sponsor’s policy, which is consistent with the UK Policy Framework for Health and Social Care Research and the Medicines for Human Use (Clinical Trials) Regulations (UK) and the NHMRC (Australia). Monitoring and audits will be conducted by University Hospitals Bristol and Weston NHS Foundation Trust (UHBW), on behalf of the Sponsor, and will ensure adherence to ICH GCP (International Conference on Harmonisation for Good Clinical Practice) and the aforementioned regulations. All trial-related documents will be made available on request for monitoring and audit by the Sponsor, the relevant REC and for inspection by MHRA and other licensing bodies.

A Trial Monitoring Plan has been developed by the Sponsors and agreed upon by the TMG and CI based on the trial risk assessment which may include on-site monitoring. Monitoring will be initiated using a risk-based approach.

### Plans for communicating important protocol amendments to relevant parties (e.g. trial participants, ethical committees) {25}

The trial has received Clinical Trial Authorisation (CTA) in the UK by the MHRA. In Australia, a Clinical Trial Notification (CTN) has been made to the TGA.

Any amendments which effect the safety (physical or mental integrity) of the participants, the scientific value of the study, the conduct or management of the study or the quality or safety of any IMP, will constitute a substantial amendment and a request to the MHRA for approval in the UK, and the TGA in Australia, will be submitted.

Any amendments to the trial protocol and other trial-related participant-facing documents will be submitted to the respective Ethics committees in the UK and Australia and receive the necessary approvals prior to implementation.

All trial sites and investigators will be notified of amendments and their date of implementation. All participants will provide consent using the procedures in the latest version of the protocol at the time of their enrolment.

### Dissemination plans {31a}

A dissemination plan will be produced with the TMG, collaborators and the autistic advisory group. The outputs will include openly accessible academic papers in leading peer-reviewed journals alongside the full report published in the NIHR Journal’s library. Findings will also be presented at relevant international and national conferences and disseminated widely to the autism community, health care providers, policymakers and the public through presentations, written briefs, blogs or social media.

## Discussion

There have been no large randomised controlled trials assessing the effectiveness or adverse effect profiles of commonly prescribed psychotropic medications in the adult autistic population [8, 24]. Previous RCTs involving SSRI medications were small and have not measured mental health outcomes [24]. As such, the results will contribute to the understanding of the treatment of anxiety in autistic adults and provide evidence on the adverse effect profile of sertraline when used with this population. This trial and all its processes have been designed with input from our autistic advisory group since the early stages of the funding application. Extensive preliminary work regarding the acceptability of RCT processes [44] and the impact of the COVID-19 pandemic on the conduct of research with the autistic population [45] has also been carried out. This methodological work and co-production within this trial will contribute to lessons on the design and conduct of future large medication trials involving autistic adults.

## Trial status

Recruitment to STRATA started in August 2021 and the 9-month internal pilot phase was successfully completed in April 2022, following which the funder green-light to proceed to full trial was received. Recruitment is expected to be completed by the end of November 2023, and follow-up of the last patient recruited is expected to be completed by 30 November 2024. The trial end date is 31 March 2025. The current protocol version is version 7.0 (31 August 2023).

### Supplementary Information

** Additional file 1. **A multicentre double-blind placebo-controlled randomised trial of SerTRaline for AnxieTy in adults with a diagnosis of Autism (STRATA).

**Additional file 2. **Participant consent form.

## Data Availability

The final trial data set will be stored as restricted data on the data.bris research data repository. Data will be made available to approved bona fide researchers only, after their host institution has signed a data access agreement. Requests for access should be made to the Chief Investigator.

## Abbreviations

5-HT: 5-Hydroxytryptamine, serotonin
ADHD: Attention-deficit hyperactivity disorder
AE: Adverse event
ANZCTR: Australian New Zealand Clinical Trials Registry
APP: Australian Privacy Principles
AR: Adverse reaction
BTC: Bristol Trials Centre
CACE: Complier average causal effect
CI: Chief Investigator
CONSORT: Consolidated Standards of Reporting Trials
CCA: Cost-consequences analysis
CTA: Clinical Trial Authorisation
CTN: Clinical Trial Notification
CUA: Cost–utility analysis
DMEC: Data Monitoring and Ethics Committee
EDC: Electronic data capture
EOI: Expression of Interest
GAD-7: Generalised Anxiety Disorder Assessment
GDPR: General Data Protection Regulation
GP: General Practitioner
HEAP: Health Economics Analysis Plan
IAPT: Improving Access to Psychological Therapies
ICH-GCP: International Conference on Harmonisation for Good Clinical Practice
ID: Intellectual Disability
IMP: Investigational Medicinal Product
ISRCTN: International Standard Randomised Controlled Trials Number
ITT: Intention-to-treat
MHRA: Medicines and Healthcare Products Regulatory Agency
NHMRC: National Health and Medical Research Council
NHS: National Health Service
NICE: National Institute for Health and Care Excellence
OCD: Obsessive-compulsive disorder
OCI-R: Obsessive Compulsory Inventory Revised
PHQ: Patient Health Questionnaire
PI: Principal Investigator
PICs: Patient Identification Centres
PIL: Participant Information Leaflet
PSS: Personal Social Services
PPI: Patient and Public Involvement
PRIME-MD: Primary Care Evaluation of Mental Disorders questionnaire
PROM: Patient Reported Outcome Measure
PTSD: Posttraumatic stress disorder
QALY: Quality-adjusted life years
QRI: QuinteT Recruitment Intervention
RBQ-2A: The Adult Repetitive Behaviours Questionnaire-2
RCT: Randomised Controlled Trial
REC: Research Ethics Committee
RGO: Research Governance Office
RUQ: Resource Use Questionnaire
SAE: Serious Adverse Event
SAP: Statistical Analysis Plan
SD: Standard Deviation
SmPC: Summary of Product Characteristics
SPIN: Social Phobia Inventory
SSRI(s): Selective Serotonin Reuptake Inhibitor(s)
SUSAR: Suspected Unexpected Serious Adverse Reaction
TGA: Therapeutic Goods Administration
TMG: Trial Management Group
TSC: Trial Steering Committee
UHBW: University Hospitals Bristol and Weston NHS Foundation Trust
UK: United Kingdom
UCL: University College London
UoB: University of Bristol
VPN: Virtual Private Network
WHODAS: World Health Organization Disability Assessment
