# Supplementary material for: Sertraline for anxiety in adults with a diagnosis of autism (STRATA): study protocol for a pragmatic, multicentre, double-blind, placebo-controlled randomised controlled trial
Source: Trials. 2024 Jan 11;25:37. doi: 10.1186/s13063-023-07847-3 (PMC10782796; doi:10.1186/s13063-023-07847-3)
Supplement: Supplementary file 1 — Additional file 1. A multicentre double-blind placebo-controlled randomised trial of SerTRaline for AnxieTy in adults with a diagnosis of Autism (STRATA). [file 13063_2023_7847_MOESM1_ESM.pdf]

A multicentre double-blind placebo-controlled randomised trial of SerTRaline for AnxieTy in adults with a diagnosis of Autism (STRATA).

Protocol

Version 7.0 31 AUGUST 2023

UNITED KINGDOM (UK)		AUSTRALIA	
**Approvals and IDs**	**Reference - UK**	**Approvals and IDs**	**Reference - Australia**
Protocol version	7.0	Protocol version	6.0
IRAS project ID	270727	-	-
Research Ethics Committee (REC)	Oxford B REC (22/SC/0296)	Research Ethics Committee (REC)	South Metropolitan Health Service, Government of Western Australia RGS0000003578
Sponsor	University of Bristol, 2019 – 4556	Sponsor	The University of Western Australia 2021/ET000456
EudraCT	2019-004312-66	CTN	CT-2021-CTN-02007-1 v3
ISRCTN	15984604	ANZCTR	ACTRN12621000801819
Funder	NIHR HTA 127337	Funder	NHMRC 1171206
NIHR CRN CPMS	46047	-	-

This protocol has regard for the HRA guidance

KEY TRIAL CONTACTS

Chief Investigator	Professor Dheeraj Rai<br>Professor of Neurodevelopmental Psychiatry<br>University of Bristol<br>Centre for Academic Mental Health<br>Population Health Sciences<br>Bristol Medical School<br>BS8 2BN<br><br>Tel: 01173313355<br>Email: Dheeraj.Rai@bristol.ac.uk
Co-investigators (applicants for funding)	Professor David Kessler, Professor of Primary Care, University of Bristol<br>Email: david.kessler@bristol.ac.uk<br><br>Professor Nicola Wiles, Professor of Epidemiology, University of Bristol<br>Email: nicola.wiles@bristol.ac.uk<br><br>Dr Stephanie J MacNeill, Lecturer in Medical Statistics, University of Bristol<br>Email: stephanie.macneill@bristol.ac.uk<br><br>Dr Nicola Mills, Senior Research Fellow in Health Services Research, University of Bristol<br>Email: nicola.mills@bristol.ac.uk<br><br>Dr Joanna Thorn, Research Fellow in Health Economics, University of Bristol<br>Email: joanna.thorn@bristol.ac.uk<br><br>Dr Ailsa Russell, Reader and Clinical Psychologist, University of Bath<br>Email: ajr39@bath.ac.uk<br><br>Mr Jack Welch, Autistic advocate and Discover insight group member/contributor<br><br>Professor Traolach Brugha, Professor of Psychiatry, University of Leicester<br>Email: tsb@leicester.ac.uk<br><br>Professor David Baldwin, Professor of Psychiatry, University of Southampton<br>Email: d.s.baldwin@soton.ac.uk<br><br>Professor Peter Langdon, Professor of Clinical and Forensic Psychology, University of Warwick<br>Email: peter.langdon@warwick.ac.uk<br><br>Professor Regi Alexander, Consultant Psychiatrist, Hertfordshire Partnership University NHS Foundation Trust<br>Email: regialexander@nhs.net<br><br>Professor Raja Mukherjee, Consultant Psychiatrist,

	Surrey and Borders Partnership NHS Foundation Trust<br>Email: raja.mukherjee@sabp.nhs.uk<br><br>Associate Professor Helen Leonard, NHMRC Senior Research Fellow and Associate Professor, The University of Western Australia<br>Email: helen.leonard@telethonkids.org.au<br><br>Dr Emma Glasson*, Senior Research Fellow/Study Coordinator, The University of Western Australia<br>Email: emma.glasson@telethonkids.org.au<br><br>Professor Sergio Starkstein*, Professor of Psychiatry, The University of Western Australia<br>Email: sergio.starkstein@uwa.edu.au<br>**Co-applicants for NHMRC funding along with Helen Leonard.*
Lead Statistician	Dr Stephanie J MacNeill, Senior Lecturer in Medical Statistics, University of Bristol<br>Email: stephanie.macneill@bristol.ac.uk
Study website	www.bristol.ac.uk/strata
**UK-specifics**	
Trial Manager (UK)	Miss Leonora Cotton<br>General trial email: strata-rct@bristol.ac.uk<br>Secure trial email: awp.strata-rct-secure@nhs.net
Central Pharmacy (UK)	University Hospitals Bristol Pharmacy<br>University Hospitals Bristol and Weston NHS Foundation Trust (UHBW)<br>University Hospitals Bristol<br>Bristol Royal Infirmary<br>Trials Pharmacy<br>Upper Maudlin Street<br>Bristol, BS2 8HW (UK)<br>Email: pharmacytrials@uhbw.nhs.uk<br>Email: Ubh-tr.pharmacytrials@nhs.net
**Australia-specifics**	
Trial Manager (Australia)	Dr Emma Glasson and Ms Katherine Hatch<br>General trial email: strata@uwa.edu.au<br>Email: emma.glasson@telethonkids.org.au and katherine.hatch@uwa.edu.au
Central Pharmacy (Australia)	Fiona Stanley Hospital Pharmacy<br>11 Robin Warren Drive<br>Murdoch Western Australia 6150 (Australia)<br>Email: FSH.PharmacyClinicalTrials@health.wa.gov.au

FUNDING AND SUPPORT IN KIND

Funder(s)	Financial and non-financial support given
NIHR Health Technology Assessment Programme<br>National Institute for Health Research<br>Evaluation, Trials and Studies Coordinating Centre<br>University of Southampton<br>Alpha House, Enterprise Road<br>Southampton, SO16 7NS (UK)<br>Telephone: 023 8059 5586<br>Fax: 023 8059 5639<br>Email: netsmonitoring@nihr.ac.uk	Grant funding (United Kingdom [UK])
National Health and Medical Research Council<br>GPO Box 1421<br>Canberra ACT 2601 (Australia)<br>Telephone: +61 2 6217 9000<br>Email: info@nhmrc.gov.au	Grant funding (Australia)
Bristol Trials Centre (BTC)<br>Bristol Medical School, Department of Population Health Sciences, University of Bristol<br>1-5 Whiteladies Road<br>Bristol, BS8 1NU (UK)<br>Telephone: 0117 928 7393<br>Email: btc-mailbox@bristol.ac.uk	Methodological expertise (UK)
Adam Taylor<br>Head of Research Governance<br>Research and Enterprise Development<br>University of Bristol, Senate House<br>Tyndall Ave<br>Bristol, BS8 1TH (UK)<br>Telephone: 0117 42 83065<br>Email: research-governance@bristol.ac.uk	Sponsorship and research governance (UK)
The University of Western Australia (M459)<br>35 Stirling Highway<br>Crawley, Perth<br>Western Australia 6009<br>Telephone: +61 8 6488 4260	Sponsorship (Australia)
South Metropolitan Health Service Human Research Ethics Committee<br>Level 2, Education Building<br>Fiona Stanley Hospital Campus<br>Postal address: Locked Bag 100, Palmyra DC, Western Australia, 6961<br>Telephone: +61 8 6152 2064<br>Email: SMHS.HREC@health.wa.gov.au	Ethics (Australia)
Research Governance Office<br>Level 2, Education Building<br>Fiona Stanley Hospital Campus<br>Postal address: Locked Bag 100, Palmyra DC, Western Australia, 6961<br>Telephone: +61 8 6152 2646<br>Email: SMHS.RGO@health.wa.gov.au	Research governance (Australia)

Eramol LtdUnit 11, Gatwick Metro Centre<br>Horley, RH6 9GA (UK)<br><br>Unit 9 North Downs Business Park<br>Limepit Lane, Sevenoaks<br>TN13 2TL (UK)<br><br>Telephone: 01293 368080<br>Email: enquiry@eramol.com	Commercial supplier and packaging
University Hospitals Bristol Pharmacy<br>University Hospitals Bristol and Weston NHS Foundation Trust (UHBW)<br>University Hospitals Bristol<br>Bristol Royal Infirmary<br>Trials Pharmacy<br>Upper Maudlin Street<br>Bristol, BS2 8HW (UK)<br><br>Email: pharmacytrials@uhbw.nhs.uk	Distribution of Investigational Medicinal Product (IMP)
Avon and Wiltshire Mental Health NHS Partnership Trust<br>Bath NHS House<br>Newbridge Hill<br>Bath, BA1 3QE (UK)<br><br>Email: awp.research@nhs.net	Host (UK)

LIST OF CONTENTS

KEY TRIAL CONTACTS 2
FUNDING AND SUPPORT IN KIND 4
LIST OF CONTENTS 6
LIST OF ABBREVIATIONS 11
TRIAL SUMMARY 13
TRIAL FLOW CHART 15
TRIAL PROTOCOL TITLE 16
1 BACKGROUND AND RATIONALE 17
2 AIMS AND OBJECTIVES 19
2.1 Aim 19
2.2 Primary objective 19
2.3 Secondary objectives 19
2.4 Primary endpoint/outcome 19
2.5 Secondary endpoints/outcomes 20
2.6 Primary outcome data 21
2.7 Secondary outcome data 21
3 TRIAL DESIGN 22
3.1 Project timetable 22
3.2 Internal pilot 22
3.3 Planned recruitment rate 22
4 TRIAL SETTING 24
5 ELIGIBILITY CRITERIA 25
5.1 Subject population 25
5.2 Inclusion criteria 25
5.3 Exclusion criteria 25
5.4 Co-enrolment in other research studies 26
6 TRIAL PROCEDURES 27
6.1 Schedule of trial assessments and outcomes (overview) 27
6.2 Identification and screening of potential participants (overview) 29
6.3 STEP 1: Participant identification and invitation to participate 32
6.3.1 Clinical appointments and/or lists 32
6.3.2 Research registers / mailing lists 33
6.3.3 Other methods and organisations 34
6.4 STEP 2: Preliminary (eligibility) screening and expression of interest 34

6.5 STEP 3: Patient safety checks (for those who are potentially eligible in Step 2 and consent to contact GP) 35
6.6 STEP 4: Inform individual of eligibility assessment outcome and arrange the Baseline (first study) appointment, where applicable....35
6.7 STEP 5: Baseline (first study) appointment (for those potentially eligible in Step 4) 36
6.7.1 Consent (main trial) 39
6.7.2 Further baseline assessments....40
6.7.3 Next steps 40
6.7.4 Randomisation 41
6.7.5 End of Baseline appointment / post-randomisation requirements 41
6.8 Follow-up assessments....42
6.8.1 Brief contact (safety checks): 1 to 2-, 4-, 8-, 12-, and 36-weeks post-randomisation....42
6.8.2 Follow up assessments: 16-, 24- and 52-weeks post-randomisation 43
6.9 Qualitative assessments 44
6.10 Thanking participants for their involvement (payments)....44
6.11 Methods/ procedures to protect against other sources of bias....44
6.11.1 Loss to follow up (attrition bias) 44
6.11.2 Measurement bias....45
6.11.3 Other sources of bias (detection bias) 45
6.12 Usual care....45
6.13 Emergency contact procedure for participants 45
6.14 Blinding....46
6.15 Unblinding....46
6.16 Emergency unblinding....46
6.17 Withdrawal from the trial (or change of permissions)....47
6.18 End of trial....47
6.18.1 Trial stopping rules 47
6.19 Carer involvement....48
7 QUINTET RECRUITMENT INTERVENTION (QRI) AND INTEGRATED QUALITATIVE RESEARCH 49
7.1 QuinteT Recruitment Intervention (QRI) *[UK only]*....49
7.1.1 QRI - Phase 1: Understanding recruitment....49
7.1.2 QRI - Phase 2: Development and implementation of recruitment strategies 50
7.2 Integrated qualitative research – experiences and acceptability of study and treatment *[UK only]* 51
7.2.1 Study staff/healthcare professional consent....51

7.2.2 Patient consent....52
7.3 Analysis of QRI and integrated qualitative research data....53
7.4 Data management, protection and patient confidentiality in relation to the QRI and qualitative research data [applicable to UK only]....53
7.5 Safeguarding patients during QRI and qualitative research....54
8 NESTED STUDY - EXPLORATION OF CARER BURDEN *[UK AND AUSTRALIA]*....55
8.1 Objective and outcome measures....55
8.2 Recruitment and consent....55
8.3 Information about the study....57
8.4 Withdrawal criteria....57
8.5 Adverse Events....57
8.6 Analysis of carer burden data....57
8.7 Data Management....57
9 INTERVENTION / INVESTIGATIONAL MEDICINAL PRODUCT (IMP)....58
9.1 General information....58
9.2 Assessment and management of risk....58
9.3 Manufacture of IMP....58
9.4 Packaging, labelling and shipping of IMP....58
9.5 Storage of IMP at central pharmacy....58
9.6 Prescribing procedure....58
9.7 Dispensing of IMP....59
9.8 Return and destruction of IMP....59
9.9 Dosing schedule / Administration and titration....59
9.10 Assessment of adherence to treatment....60
9.11 Common side-effects....60
9.12 Titration for side-effects....61
9.13 Discontinuation of trial medication....61
9.14 Post-trial....61
9.15 Drug accountability....61
9.16 Intervention and IMP COVID-19 considerations....62
10 PHARMACOVIGILANCE....63
10.1 Operational definitions....63
10.2 Classification of severity....64
10.3 Classification of relatedness....64
10.4 Classification of expectedness....64

10.5 Adverse events (AEs) classification flowchart ... 65
10.6 Identification of AEs ... 65
10.7 Recording and reporting procedures ... 66
10.7.1 Non-serious AEs ... 66
10.7.2 Serious AEs (SAE/SAR/SUSARs) ... 67
10.8 Notification of deaths ... 69
10.9 Pregnancy ... 69
10.10 Overdose ... 69
10.11 Urgent safety measures (USMs) ... 69
10.12 Safety reporting period ... 70
10.13 Safety Update Reports ... 70
11 STATISTICS AND HEALTH ECONOMICS ANALYSIS ... 71
11.1 Sample size calculation ... 71
11.1.1 Main trial ... 71
11.1.2 Carer burden sub-study ... 71
11.2 Statistical analysis ... 71
11.2.1 Summary of baseline data ... 71
11.2.2 Primary outcome analysis ... 72
11.2.3 Secondary outcome analysis ... 72
11.2.4 Subgroup analysis ... 72
11.2.5 Adjusted analysis ... 72
11.2.6 Carer burden sub-study ... 72
11.3 Analysis of safety endpoints ... 73
11.4 Missing data ... 73
11.5 Economic evaluation ... 73
11.5.1 Aim ... 73
11.5.2 Outcomes ... 73
11.5.3 Cost measurement ... 73
11.5.4 Analysis ... 74
12 DATA MANAGEMENT ... 75
12.1 Source data and documents ... 75
12.2 Data handling ... 75
12.3 Database platform(s) ... 76
12.3.1 Administrative data ... 76
12.3.2 Clinical data ... 76

12.4 Storage and access to data........76
12.5 Archiving and destruction of trial materials........77
12.6 Access to the final trial data set........77
13 TRIAL MANAGEMENT........78
13.1 Trial management group (TMG)........78
13.2 Trial Steering committee (TSC)........78
13.3 Data monitoring committee (DMC)........78
13.4 Patient and public involvement (PPI)........78
13.5 Sponsor and funding........78
14 MONITORING, AUDIT AND INSPECTION........81
14.1 Monitoring........81
14.2 Protocol compliance........81
14.3 Notification of Serious Breaches to GCP and/or the protocol........82
15 ETHICAL AND REGULATORY CONSIDERATIONS........83
15.1 Governance and legislation........83
15.2 Research Ethics Committee (REC) review and reports........83
15.3 MHRA and TGA review and reports........84
15.4 Peer review........84
15.5 Poor quality data........84
15.6 Financial and other competing interests for the chief investigator, PIs at each site and committee members for the overall trial management........85
15.7 Indemnity........85
16 DISSEMINATION POLICY........86
17 SIGNATURE PAGE........87
18 REFERENCES........88
APPENDIX 1 STRATA Dose titration and stopping instructions........92
APPENDIX 2 COVID-19 Considerations........94
APPENDIX 3 Amendments to Protocol........96

LIST OF ABBREVIATIONS

A&E	Accident & Emergency
ADHD	Attention Deficit Hyperactivity Disorder
AE	Adverse Event
ANZCTR	Australian New Zealand Clinical Trials Registry
APP	Australian Privacy Principles
AR	Adverse Reaction
BNF	British National Formulary
BTC	Bristol Trials Centre
C&C	Confirmation of Capacity and Capability
CCA	Cost–Consequences Analysis
CI	Chief Investigator
CONSORT	Consolidated Standards of Reporting Trials
COVID-19	Coronavirus
CRF	Case Report Form
CRN	Clinical Research Network
CSO	Clinical Study Officer
CTA	Clinical Trial Authorisation
CTN	Clinical Trial Notification
CUA	Cost–Utility Analysis
DIBD	Developmental International Birth Date
DMC	Data Monitoring Committee
DSUR	Development Safety Update Report
EC	European Commission
EEA	European Economic Area
EOI	Expression of Interest
EU	European Union
GAD-7	Generalised Anxiety Disorder Assessment
GDPR	General Data Protection Regulation
GP	General Practitioner
HES	Hospital Episode Statistics
HRA	Health Research Authority
IAPT	Improving Access to Psychological Therapies
ICH-GCP	International Conference on Harmonisation for Good Clinical Practice
ID	Intellectual Disability
I.D	Identification
IMP	Investigational Medicinal Product
ISF	Investigator Site File
ISRCTN	International Standard Randomised Controlled Trials Number
ITT	Intention-to-treat
MHRA	Medicines and Healthcare Products Regulatory Agency
MIA	Manufacturer and Importation Authorisation
NHMRC	National Health and Medical Research Council
NHS	National Health Service
NHS R&D/R&I	National Health Service Research & Development/Research & Innovation
NICE	National Institute for Health and Care Excellence
NIHR CRN	National Institute for Health Research Clinical Research Network
NIHR HTA	National Institute for Health Research Health Technology Assessment

OCD	Obsessive-Compulsive Disorder
OCI-R	Obsessive Compulsory Inventory Revised
PHQ	Patient Health Questionnaire
PI	Principal Investigator
PICs	Patient Identification Centres
PIL	Participant Information Leaflet
PPE	Personal Protective Equipment
PPI	Patient and Public Involvement
PQ	Participant Questionnaires
PROM	Patient reported outcome measure
PSS	Personal Social Services
PTSD	Post Traumatic Stress Disorder
QALY	Quality Adjusted Life Years
QP	Qualified Person
QRI	QuinteT Recruitment Intervention
RA	Research Assistant
RCT	Randomised Controlled Trial
R&D	Research and Development
RGO	Research Governance Office
REC	Research Ethics Committee
RSI	Reference Safety Information
RUQ	Resource-Use Questionnaire
SAE	Serious Adverse Event
SAR	Serious Adverse Reaction
SAP	Statistical Analysis Plan
SD	Standard Deviation
SEAR	Screened, Eligible, Approached, Randomised (SEAR) framework
SLA	Service Level Agreement
SmPC	Summary of Product Characteristics
SOP	Standard Operating Procedure
SPIN	Social Phobia Inventory
SQL	Structured Query Language
SSAR	Suspected Serious Adverse Reaction
SSL	Secure Sockets Layer
SSRI(s)	Selective Serotonin Reuptake Inhibitor(s)
SUSAR	Suspected Unexpected Serious Adverse Reaction
TGA	Therapeutic Goods Administration
TMF	Trial Master File
TMG	Trial Management Group
TSC	Trial Steering Committee
UHBW	University Hospitals Bristol and Weston NHS Foundation Trust
UK	United Kingdom
UKCRC	UK Clinical Research Collaboration
UoB	University of Bristol
USM	Urgent Safety Measure
UWA	The University of Western Australia
WHODAS	World Health Organization Disability Assessment

TRIAL SUMMARY

**Trial title**	A multicentre double-blind placebo-controlled randomised trial of SerTRaline for AnxieTy in adults with a diagnosis of Autism (STRATA)	
**Short title**	SerTRaline for AnxieTy in adults with a diagnosis of Autism. A randomised controlled trial	
**Acronym**	STRATA	
**Chief Investigator**	Professor Dheeraj Rai. Professor of Neurodevelopmental Psychiatry<br>University of Bristol, UK	
**Sponsor**	• **UK:** University of Bristol (UoB)<br>• **Australia:** The University of Western Australia (UWA)	
**Funder**	• **UK:** National Institute for Health Research Health Technology Assessment Programme (NIHR HTA; reference 127337)<br>• **Australia:** National Health and Medical Research Council (NHMRC; reference 1171206)	
**Trial design**	A two parallel group multi-centre pragmatic randomised controlled trial (RCT) of sertraline versus placebo for reducing anxiety in adults with a diagnosis of autism	
**Planned sample size**	306	
**Inclusion criteria**	Adults (≥18-years); A diagnosis of autism made by a specialist including those with a co-occurring mild intellectual disability [autism diagnostic terms may include autism/autistic spectrum disorder or other variations, Asperger syndrome/disorder or pervasive developmental disorder]; and Anxiety as measured by GAD-7 score ≥10 at screening	
**Exclusion criteria**	Prescribed and regular use of serotonergic antidepressant/anxiolytic in preceding 8-weeks at antidepressant dosage; Prescribed an irreversible monoamine oxidase inhibitor or Pimozide in the preceding 8-weeks; Diagnosis of moderate-severe intellectual disability (ID); Inability to provide informed consent and complete study assessments / questionnaires; Currently valid diagnosis of bipolar disorder, manic or hypomanic episodes, or psychosis; Currently uncontrolled epilepsy; Known current alcohol or drug use problem making sertraline prescription unsafe; Known allergies to sertraline or placebo/excipients; Currently enrolled in another competing RCT; Women who are pregnant, are planning pregnancy (during the trial period), or breastfeeding; History of severe liver impairment; Bleeding disorders such as such as haemophilia, Christmas disease and von Willebrands disease, as well as those with past medical history of bleeding gastric or duodenal ulcers or other significant bleeding disorders; History of Long QT syndrome or Torsade de Pointes; Swallowing difficulties or inability to take medication in capsule form; Currently using St. John's Wort	
**Number of study centres**	STRATA will be delivered through Autism services in (at least) four regional centres in the UK and (at least) one in Western Australia. Within each centre there can be several recruiting sites. Additional centres/sites will be identified if required	
**Treatment duration**	52-weeks in total (primary outcome at 16-weeks post-randomisation)	
	**Objectives**	**Outcome Measures**
**Primary**	To determine the difference in Generalised Anxiety Disorder Assessment (GAD-7) anxiety scores at 16-weeks between adults with a diagnosis of autism treated with sertraline and those treated with placebo	Patient reported outcome measure (PROM) GAD-7 anxiety score at 16-weeks post-randomisation
**Secondary**	**i)** To describe the adverse effects reported by adults with a diagnosis of autism treated with sertraline versus those treated with placebo over 52-weeks	Modified Toronto side effects scale, and open-ended questions (including suicidality item)
	**ii)** To determine the effect of up to 52-weeks of treatment with sertraline versus placebo on:	-
	**a)** GAD-7 score and proportionate change in GAD-7 scores including response (50% reduction in GAD-7 scores)	GAD-7

	**b)**	patient reported effect of medication on symptoms	Study-specific questionnaire
	**c)**	social anxiety	Social Phobia Inventory (SPIN)
	**d)**	obsessive compulsive symptoms	Obsessive Compulsory Inventory Revised (OCI-R)
	**e)**	panic attacks	Brief Patient Health Questionnaire (PHQ) from Primary Care Evaluation of Mental Disorders (PRIME-MD)
	**f)**	repetitive behaviours	Adult Repetitive Behaviours Questionnaire-2 (RBQ-2A)
	**g)**	meltdowns	Single item ‘had a meltdown’ added to GAD-7 scale
	**h)**	depressive symptoms	Patient Health Questionnaire-9 (PHQ-9)
	**i)**	composite anxiety and depressive symptoms	Sum of PHQ-9 and GAD-7 Scores
	**j)**	functioning and disability	World Health Organization Disability Assessment Schedule 2.0 (WHODAS 2.0)
	**k)**	quality of life	EQ-5D-5L questionnaire
	**l)**	carer burden and carer quality of life	Caregiver Burden Scale, Carers Experience Scale (CES) and EQ-5D-5L questionnaire
	**iii)**	To measure adherence to the study medication	Questionnaire (adapted from GENPOD and PANDA trials)
	**iv)**	To determine the cost effectiveness of sertraline treatment for anxiety in adults with a diagnosis of autism	EQ-5D-5L questionnaire (to calculate QALYs) and patient resource use questionnaire
	**v)**	To explore participants’ acceptability, experiences of, and adherence to, study processes and treatment	Qualitative interviews with participants
**Study duration**	• Funding start date: 01 October 2019<br>• Anticipated duration: 66 months<br>• Anticipated end date: 31 March 2025		

TRIAL FLOW CHART

Total recruitment phase (28-months): (a) Internal pilot phase: ≥5 centres, 9-months recruitment; and **(b) Main phase:** same pilot centres, additional 19-months recruitment. **Follow-up phase**: 40-months total.

QuinteT Recruitment Intervention (QRI)

[UK sites only]

QRI to understand and optimise recruitment to the trial.

Phase I: Developing an understanding of recruitment processes/ challenges.

Phase II: Feedback to CI/TMG, sites and trial recruiters; and implementation of plan to optimise recruitment.

Conducted during first 18- months of recruitment, including Pilot phase.

Pre-screening and Eligibility

Pre-screening: individuals with a diagnosis of autism who score ≥10 on the GAD-7, and/or individuals who have a diagnosis of autism and who may be seeking support for their anxiety will be invited to take part.

Eligibility: Confirmed following: (i) completion of Preliminary Screening and Expression of Interest Form; (ii) safety check by GP/PI (doctor) for those who meet minimal eligibility requirements and consent to contact doctor/GP and (iii) conversation with member of local research team (Baseline).

Enrolment

Baseline (first study) appointment:
Review and confirm eligibility; obtain informed consent; additional data collection (case report forms); baseline questionnaires; and conduct randomisation.

Randomised Allocation (n=306)

Control: Placebo (n=153)

Intervention: Sertraline (n=153)

Integrated Qualitative Research

[UK sites only]

Clinical qualitative study to explore participants' decisions, acceptability and experiences of trial participation and study medication.

Conducted alongside the QuinteT Recruitment Intervention (QRI).

Follow Up

1 to 2-, 4-, 8- and 12-weeks post-randomisation[A]: Brief case report form / questionnaire data collection on safety and adherence to medication; including (at least) GAD-7, PHQ-9, modified Toronto side effects scale, suicidality item, and other medications. Plus, EQ-5D-5L at 12-weeks.

16-weeks post-randomisation[A]: Case report form / questionnaire data collection, including (at least) GAD-7*, adverse effects, quality of life, adherence to medication and other anxiety and quantitative outcomes. *(*Primary Outcome)*

36-weeks post-randomisation[A]: Brief case report form / questionnaire data collection on safety and adherence to medication; including (at least) GAD-7, PHQ-9, modified Toronto side effects scale, suicidality item, and other medications.

24- and 52-weeks post-randomisation[A]: Case report form / questionnaire data collection, including (at least) GAD-7, adverse effects, quality of life, adherence to medication, health and social care resource use and other anxiety and quantitative outcomes.

End of Participant Involvement

[A]See Tables 1 and 3 for details of outcomes, outcome measures and schedule of trial assessments and key participant-related procedures.

Figure 1 Trial flowchart

TRIAL PROTOCOL TITLE

A multicentre double-blind placebo-controlled randomised trial of SerTRaline for AnxieTy in adults with a diagnosis of Autism (STRATA).

1 BACKGROUND AND RATIONALE

Autism spectrum disorders (henceforth autism) are developmental conditions characterised by difficulties in social interaction and communication (1), associated with significant long-term personal, familial and societal cost (2). Autistic adults[1], particularly those without intellectual disabilities (ID) have a greater burden of mental health problems than the general population (3-6), higher rates of premature mortality (7, 8), with suicide as an important contributor (7).

Anxiety is common in autistic adults (1, 9, 10), and the distress and avoidance behaviours related to it are often more disabling than difficulties related to autism. The reported rates of anxiety disorders and related conditions in adults with autism vary widely (28–77%) because most research has been done on selected clinical samples, and a recent meta-analysis reported a pooled lifetime prevalence of 42% (11). Social phobia, generalised anxiety disorder (GAD), and obsessive-compulsive disorder (OCD) are common diagnoses, but anxiety in autism often does not align with the rigid diagnostic criteria for individual anxiety disorders (12). This observation was supported in a large total population study we have recently completed in Stockholm County, where non-specific anxiety disorders were the most common form of anxiety disorder recorded (12.9% of 8,248 adults with a diagnosis of autism vs 3.5% of 534,430 general population controls), although all specific types of anxiety disorders were over-represented in autistic adults compared with the general population (13).

Most anxiety in the population is managed in primary care, although General Practitioners (GPs) often make prescribing decisions based on anxiety symptoms rather than make diagnoses of specific anxiety disorders (14, 15). Selective serotonin reuptake inhibitors (SSRIs) are commonly used antidepressants but are also first line medications for all anxiety disorders (16). The antidepressant action of SSRIs occurs within 1 week, with statistical separation from placebo often evident in 2 to 4-weeks, but it is thought that effects on anxiety disorders may take longer. However, findings from a recent randomised controlled trial (RCT) in the United Kingdom (UK) primary care population suggested a reduction in anxiety symptoms within 6-weeks of use of the SSRI sertraline. It is also thought that people with anxiety may be more prone to adverse effects of SSRIs, particularly psychomotor restlessness (akathisia) and initial worsening of symptoms (17). Prescribing guidelines therefore suggest that for most anxiety disorders, SSRIs should be started at half the normal starting dose and titrated upwards up to the maximum tolerated dose (18). It is understood that response is generally observed within 6-weeks and continues to increase over time (19). The optimal duration of SSRI treatment for anxiety is still unclear, but guidelines suggest that treatment be continued for at least 6- to 12-months (16, 20). It is recommended that patients on SSRIs should be assessed for the emergence of restlessness, increased anxiety and emergence of suicidal ideation (17).

SSRIs are thought to act via increased levels of available neurotransmitter serotonin (5-hydroxytryptamine, 5HT) in the synaptic cleft. The 5HT system is important in autism (21) and elevated 5HT in whole blood and platelets (22), and alterations in the developmental trajectory of brain 5HT synthesis activity (23) in individuals with a diagnosis of autism have been reported. It has been suggested that increased 5HT uptake or storage in the presynaptic neuron could lead to decreased synaptic 5HT in individuals with autism (21), and this may underpin greater levels of anxiety, potential for benefits of SSRIs and also potential for greater sensitivity to adverse effects via increase in peripheral serotonin in this population.

[1] please note we use disability first language as opposed to person first language i.e. person with autism on the recommendation of our autistic advisory group.

There is clinical equipoise in relation to SSRI use for anxiety symptoms in autistic adults. The three RCTs of SSRIs in autistic adults to date had small samples (two for fluoxetine with n=6 (23) and n=37 (24) participants respectively, and one for fluvoxamine, n=30 (25)) with a focus on repetitive behaviours rather than anxiety, although the latter study reported improvement in maladaptive behaviours, aggression and social relatedness. Consequently, the British Association for Psychopharmacology consensus guidelines for autism conclude that there is insufficient information regarding the effectiveness or side effect profile of SSRIs in the treatment of anxiety in autism and call for large scale trials with adequate follow-up (26).

Research on interventions to help mental health problems were two of the top 10 (including #1) priorities identified by the autism community, clinicians, researchers and other stakeholders in the recent James Lind Alliance and Autistica priority setting exercise (27). SSRIs are widely prescribed in this population but without adequate evidence for effectiveness or understanding of adverse effects. It is therefore essential that we get a better understanding of this topic. Such research has the potential to improve evidence-based care for autistic adults and lead to improvements in mental health and quality of life in this underserved population.

2 AIMS AND OBJECTIVES

2.1 Aim

To determine the effectiveness and cost effectiveness of the SSRI sertraline in reducing symptoms of anxiety and improving quality of life in adults with a diagnosis of autism compared with placebo and to quantify any adverse effects.

2.2 Primary objective

To determine the difference in Generalised Anxiety Disorder Assessment (GAD-7) (28) anxiety scores at 16-weeks between adults with a diagnosis of autism treated with sertraline and those treated with placebo.

2.3 Secondary objectives

i) To describe the adverse effects reported by adults with a diagnosis of autism treated with sertraline versus those treated with placebo over 52-weeks.

ii) To determine the effect of up to 52-weeks of treatment with sertraline versus placebo on:

  a) GAD-7 score and proportionate change in GAD-7 score including response (50% reduction in GAD-7 scores)
  b) patient reported effect of medication on symptoms
  c) social anxiety
  d) obsessive compulsive symptoms
  e) panic attacks
  f) repetitive behaviours
  g) meltdowns
  h) depressive symptoms
  i) composite anxiety and depressive symptoms
  j) functioning and disability
  k) quality of life
  l) carer burden *(data will be collected in the Carer Burden Sub-Study as detailed in Section 8)*

iii) To measure adherence to the study medication.

iv) To determine the cost effectiveness of sertraline treatment for anxiety in adults with a diagnosis of autism.

v) To explore participants' acceptability, experiences of, and adherence to, study processes and treatment.

2.4 Primary endpoint/outcome

The primary outcome is the patient reported outcome measure (PROM) GAD-7 anxiety score at 16-weeks post-randomisation. Table 1 summarises the primary outcome and measure (tool) for this study.

2.5 Secondary endpoints/outcomes

Secondary outcomes are summarised in Table 1, below. Outcomes will be measured at 16-, 24- and 52-weeks post-randomisation, with brief data collection on safety and medication adherence at 1 to 2-, 4-, 8-, 12- and 36-weeks post-randomisation. See Table 3 for full details of trial assessments and time-points.

Table 1 Summary of primary and secondary outcomes and measures (tools)

Outcome	Tool / method
**Primary Outcome**	
To determine the difference in Generalised Anxiety Disorder Assessment (GAD-7) anxiety scores at 16-weeks between adults with a diagnosis of autism treated with sertraline and those treated with placebo	GAD-7 anxiety score
**Secondary Outcome**	
**i)** To describe the adverse effects reported by adults with a diagnosis of autism treated with sertraline versus those treated with placebo over 52-weeks	Modified Toronto side effects scale and open-ended questions (including suicidality item)
**ii)** To determine the effect of up to 52-weeks of treatment with sertraline versus placebo on:	-
**a)** GAD-7 score and proportionate change in GAD-7 score including response (50% reduction in GAD-7 scores)	GAD-7
**b)** patient reported effect of medication on symptoms	Study-specific questionnaire
**c)** social anxiety	Social Phobia Inventory (SPIN)
**d)** obsessive compulsive symptoms	Obsessive Compulsory Inventory Revised (OCI-R)
**e)** panic attacks	Brief Patient Health Questionnaire (PHQ) from Primary Care Evaluation of Mental Disorders (PRIME-MD)
**f)** repetitive behaviours	Adult Repetitive Behaviours Questionnaire-2 (RBQ-2A)
**g)** meltdowns	Single item ‘had a meltdown’ added to GAD-7 scale
**h)** depressive symptoms	Patient Health Questionnaire-9 (PHQ-9)
**i)** composite anxiety and depressive symptoms	Sum of PHQ-9 and GAD-7 Scores
**j)** functioning and disability	World Health Organization Disability Assessment Schedule 2.0 (WHODAS 2.0)
**k)** quality of life	EQ-5D-5L questionnaire
**l)** carer burden and quality of life	Caregiver Burden Scale, Carer Experience Scale (CES) and EQ-5D-5L questionnaire
**iii)** To measure adherence to the study medication	Questionnaire (adapted from GENPOD and PANDA trials)
**iv)** To determine the cost effectiveness of sertraline treatment for anxiety in adults with a diagnosis of autism	EQ-5D-5L (to calculate QALYs) and study-specific patient resource use questionnaire
**v)** To explore participants’ acceptability, experiences of, and adherence to, study processes and treatment.	Qualitative interviews with participants

2.6 Primary outcome data

The primary outcome for this trial is GAD-7 (28) score at 16-weeks post-randomisation as a continuous outcome. The GAD-7 is a 7-item patient reported anxiety measure and is a key outcome measure used in the UK Improving Access to Psychological Therapies (IAPT) services (29) and can be easily used in primary care, where management of anxiety in adults with a diagnosis of autism is likely to happen. It was used in a recent feasibility RCT of adults with a diagnosis of autism (3) and our autistic advisory group members found the questions easy to follow and completed it in <5 minutes. Using the threshold score of 10, GAD-7 has 89% sensitivity and 82% specificity for generalised anxiety disorder and is also good at screening panic disorder, social anxiety disorder, and posttraumatic stress disorder (PTSD) (30). As a secondary outcome based on GAD-7, we will also estimate proportionate change in GAD-7 scores including defining a binary 'response' variable with 50% reduction in GAD-7 score compared to baseline. GAD-7 will be measured at baseline, 1 to 2-, 4-, 8-, 12-, 16-, 24-, 36- and 52-weeks post-randomisation.

See Table 3 for full details of trial assessments and timepoints.

2.7 Secondary outcome data

Understanding adverse effects related to sertraline treatment is an important secondary outcome in this trial. We will measure adverse effects using a list of items based on the Toronto side effects scale which has been designed to measure antidepressant adverse effects and has been used in several large trials of antidepressants (31, 32). We will supplement this with open ended questions, including suicidality items and the Patient Health Questionnaire (PHQ-9) (33) as used for the ADEPT study (3). The GAD-7 scores at follow up will indicate any initial worsening of anxiety. Adverse effects data will be collected at baseline, 1 to 2-, 4-, 8-, 12-, 16-, 24-, 36- and 52-weeks post-randomisation.

We will also include different facets of anxiety (e.g. social anxiety, obsessive compulsive symptoms) including those important in autism but not adequately captured in general anxiety screens and other features requested in the commissioning brief. These include social anxiety (SPIN) (34); obsessive compulsive symptoms (OCI-R) (35); panic attacks (Brief PHQ from PRIME-MD) (36) and repetitive behaviours (RBQ-2A) (37). These data will be collected at baseline, 16-, 24- and 52-weeks post-randomisation.

Other secondary outcomes will include depressive symptoms (PHQ-9) (baseline, 1 to 2-, 4-, 8-, 12-, 16-, 24-, 36- and 52-weeks post-randomisation), and questions about adherence to medications (1-2, 4-, 8-, 12-, 16-, 24-, 36- and 52-weeks post-randomisation) adapted from the GENPOD (38) and PANDA (39) trials. Functioning and disability (WHODAS) (40) and Quality of Life/Utility (EQ-5D-5L) (41) measured at baseline, 16-, 24- and 52-weeks post-randomisation, plus Quality of Life/Utility (EQ-5D-5L) at 12-weeks post-randomisation. Carer burden and quality of life (Caregiver burden scale (42), Carers Experience Scale (CES; (43-45); and EQ-5D-5L (41)) will be measured at baseline, 16- and 52-weeks post-randomisation; see Section 8 for details of the Carer Burden sub-study.

3 TRIAL DESIGN

A two parallel group multi-centre pragmatic RCT of sertraline versus placebo for reducing anxiety in adults with a diagnosis of autism.

3.1 Project timetable

The funding start date for this trial is 01 October 2019, and the study duration was formally increased from 48 months to 66 months in October 2022. This will see recruitment continue until November 2023, follow up until November 2024 and the trial ending in March 2025.

3.2 Internal pilot

Following set-up, we will carry out an internal pilot study for nine months, first opening the South West England centre, followed by staggered openings of other centres (Western Australia, the Surrey, East of England, and East Midlands centres). We aim to recruit 78 patients across the five centres by the end of the 9-month pilot study (see Table 2). We will employ a traffic-light system to judge the success of our internal pilot (46). If we achieve at least 70% of our target for recruitment, then we will continue the study (Go - Green). If we are not reaching our 100% recruitment target, we will explore reasons for under recruitment and whether any modifications to recruitment processes are required dependent on the emerging recruitment rate trajectory. If we achieve between 50% and 69% (Amend - Amber) of our recruitment and/or have not commenced recruitment at any site, then we will discuss with our Trial Steering Committee (TSC) whether we should make any major changes to the recruitment strategy (e.g. additional centres) and discuss whether the study should continue. If we recruit less than 50% (Stop - Red) of our recruitment targets, and have not commenced recruitment in other sites, then we will stop the trial, unless there is a strong case that unanticipated remediable factors have been identified and can be addressed after further discussion with the funder.

Table 2 Internal Pilot: Stop/Amend/Go criteria

	**Participants**	**Anticipated action**
Go (Green)	55-78 participants ($\geq$70% of expected)	Continue – Trial Management Group (TMG) will monitor recruitment rates closely
Amend (Amber)	39-54 participants (50%-69% of expected) and recruitment not commenced in all 5 centres	Identify remediable factors, discuss with TMG and TSC. Submit recovery plan to funder (NIHR HTA)
Stop (Red)	0-38 participants (<50% of expected) and recruitment not commenced in all 5 centres	Stop the trial, unless there is a strong case that unanticipated remediable factors have been identified and can be addressed after further discussion with the funder

3.3 Planned recruitment rate

The planned recruitment for STRATA is 306 participants from (at least) five centres; details about the trial setting are provided in the next section (Section 4). A 24-month recruitment period was originally considered sufficient to identify, contact and consent 306 eligible participants. This was formally extended by 4 months in October 2022, to 28-months. In our updated recruitment progression estimates (Figure 2), we correctly assumed that the five centres noted in Section 3.2 would be recruiting by the end of the 9-month internal pilot phase (staggered openings proposed).

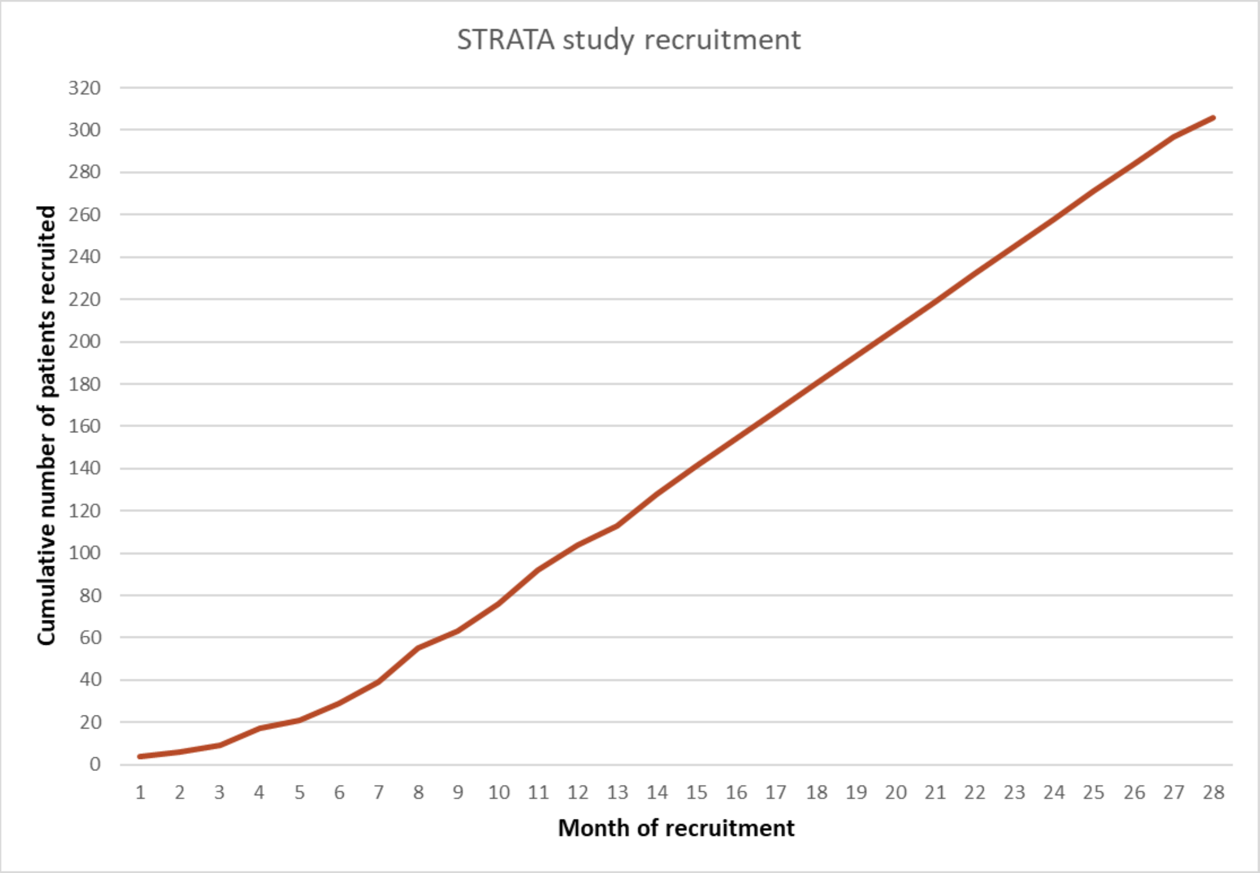

[a]*Total expected recruitment is 306 over 28-months across the five centres.*

Figure 2 Participant recruitment projections over 28-month period (subject to change)

4 TRIAL SETTING

This trial will be delivered through autism services in regional centres in the UK and Western Australia.

The areas which initial centres cover are:

1) South West England
2) Surrey, Hampshire and Portsmouth
3) East of England
4) East Midlands
5) Western Australia

Further recruitment from cohorts/registries and Patient Identification Centres (PICs) can take place, if required. Within each centre there can be several recruiting sites which may include sites within mental health and/or learning disability service providers, social enterprises, primary care, University primary care/disability services, community organisations and charities.

Additional centres/sites will be identified if required.

5 ELIGIBILITY CRITERIA

5.1 Subject population

Adults with a diagnosis of autism and symptoms of anxiety who would consider medication to help with anxiety.

5.2 Inclusion criteria

- Adults aged ≥18-years.
- A diagnosis of autism made by a specialist including those with a co-occurring mild intellectual disability (autism diagnostic terms may include autism/autistic spectrum disorder or other variations, Asperger syndrome/disorder or pervasive developmental disorder).
- Anxiety as measured by GAD-7 (28) score ≥10 at screening.

5.3 Exclusion criteria

- Prescribed and regularly using a serotonergic antidepressant/anxiolytic at antidepressant doses in preceding 8-weeks; these include SSRI and non-SSRI antidepressants including tricyclic antidepressants. Potential participants who are prescribed low (i.e. non-antidepressant) doses of these medications for other indications (e.g. neuropathic pain); or those who had no such medication for the majority of the preceding 8 weeks (e.g. tried for a few days before stopping) may be considered eligible where the PI confirms this is consistent with usual clinical practice. Individuals regularly using these medications wishing to participate could do so after a washout period of 8-weeks.
- Prescribed an irreversible monoamine oxidase inhibitor (Phenelzine, Isocarboxazid or Tranylcympromine) or Pimozide in the preceding 8-weeks.
- Diagnosis of moderate-severe intellectual disability (ID). People who have up to mild ID will be eligible. For the purpose of this study, a person with known ID will be considered as having a mild ID if they are able to provide written informed consent, and the ability to understand and answer the study questionnaires with the help of reasonable adjustments, if necessary.
- Inability to provide informed consent and complete study assessments/questionnaires.
- Currently valid diagnosis of bipolar disorder, manic or hypomanic episodes, or psychosis. Individuals with historical diagnoses where there is clinical consensus or strong suspicion that these diagnoses are no longer valid (e.g. presentations historically labelled as mania/psychosis now considered to be explained by autism) may be considered eligible based on PI discretion.
- Currently uncontrolled epilepsy.
- Known current alcohol or drug use problem (i.e. if recorded in patient/medical notes and the GP/PI considers it unsafe to co-prescribe sertraline).
- Known allergies to sertraline or placebo/excipients.
- Currently enrolled in another RCT (see Section 5.4, below).
- Women who are pregnant, are planning pregnancy during the trial period, or breastfeeding.
- History of severe liver impairment.

- Bleeding disorders such as such as haemophilia, Christmas disease and von Willebrand's disease, as well as those with past medical history of bleeding gastric or duodenal ulcers or other significant bleeding disorders.
- History of Long QT syndrome or Torsade de Pointes.
- Swallowing difficulties or inability to take medication in capsule form.
- Currently using St. John's Wort.

5.4 Co-enrolment in other research studies

Competing study: Co-enrolment in the STRATA study and another competing study (e.g. RCT, interventional study) should be avoided due to potential impact on the study objectives and patient burden and safety. If a centre/site team become aware that a participant has enrolled in a 'potentially competing' study whilst taking part in STRATA, they should inform the central research team (UoB). The research team will evaluate whether it is appropriate for the patient to continue participating in STRATA.

Non-competing study: If participants enrolled in the STRATA study express interest in enrolling in other non-competing studies (e.g. one-off interview(s) or questionnaire(s)) and informs their centre/site, the participant's centre/site team should advise that they consider factors, such as potential burden to them, social support and distances necessary to travel, before taking part. The decision to participate (co-enrol) in another non-competing study will ultimately lie with the individual (participant). If the participant's centre/site team become aware, they should inform the central research team (UoB).

6 TRIAL PROCEDURES

Three hundred and six (306) patients will be recruited and randomised (enrolled) over a 28-month period. This section outlines the key trial procedures from identification of potential participants through to end of trial.

6.1 Schedule of trial assessments and outcomes (overview)

Table 3 (next page) depicts the key assessments/outcome measures and participant-related procedures scheduled at various trial timepoints. To summarise, participants in the trial will undergo:

- Identification and screening contact; detailed in Sections 6.2 - 6.6.
- Consent and randomisation (enrolment); detailed in Section 6.7.
- Brief contact (safety checks) at 1 to 2-, 4-, 8-, 12-, and 36-weeks post randomisation; detailed in Section 6.8.1.
- Assessments at baseline (0-weeks) and follow up at 16- (primary outcome), 24- and 52-weeks post-randomisation; detailed in Section 6.8.2.

For all outcome assessments, delegated research staff at the research centres (including the research assistant (RA) and Clinical Research Network (CRN) Clinical Study Officers (CSOs) where applicable), will be blinded to the participant’s treatment allocation. See Section 6.14 for further details about blinding.

Table 3 Trial assessments and key participant-related procedures

**Data collection timepoint (→)**	**Pre-randomisation**	**Point of randomisation**	**Post-randomisation**							
**Key data capture (measures) / trial procedures (↓)**	**Identification / Screening**	**Baseline**	**1 to 2-weeks[A]**	**4-weeks[A]**	**8-weeks[A]**	**12-weeks[A]**	**16-weeks**	**24-weeks**	**36-weeks[A]**	**52-weeks**
Screening & consent to contact GP	●									
Eligibility assessment	●	●								
Consent to join trial & randomisation		●								
GAD-7 *(Primary Outcome at 16-weeks)*	●	●	●	●	●	●	●	●	●	●
Case report form(s)	●	●	●	●	●	●	●	●	●	●
Revised clinical interview schedule (CIS-R)		●								
Adult ADHD Self-Report Scale (ASRS) Part A		●								
Depressive symptoms (PHQ-9)		●	●	●	●	●	●	●	●	●
Physical symptoms / adverse effects (modified Toronto side effects scale & open-ended questions)		●	●	●	●	●	●	●	●	●
Information on other medications		●	●	●	●	●	●	●	●	●
Social anxiety (SPIN)		●					●	●		●
Panic attacks (Brief PHQ PRIME-MD)		●					●	●		●
Obsessive compulsive symptoms (OCI-R)		●					●	●		●
Repetitive behaviours (RBQ-2A)		●					●	●		●
Functioning and disability (WHODAS 2.0)		●					●	●		●
Quality of life / utility (EQ-5D-5L)		●				●	●	●		●
Medication prescribing and dispensing[B]		←								→
Adherence to study medication			●	●	●	●	●	●	●	●
Patient reported effect of drug on symptoms			●	●	●	●	●	●	●	●
Assessment on blinding			●	●	●	●	●	●	●	●
Health economic resource use questions								●		●
Unblinding[C]										●
Qualitative interviews with patients (UK only)[D]					←					→
**<u>Carer Sub-Study:</u>** Caregiver Burden Scale, Carer Experience Scale (CES) & EQ-5D-5L		◊					◊			◊

***Key**: ● data capture / outcome measures; completion methods may vary depending on participant preferences. ◊ completed by caregiver. [A]Weeks 1-12 & 36 are brief safety checks (see Section 6.8.1); [B]Medication dispensing by study pharmacies after randomisation confirmation; see Appendix 1 for dispensing schedule. [C]Timepoint may vary given option of unblinding if participant withdraws before 52-weeks (see Sections 6.15 & 6.17). [D]Timepoint will vary but anticipated conduct is sometime between 4- to 52-weeks post-randomisation (see Section 7).*

6.2 Identification and screening of potential participants (overview)

Figure 3 (next page) illustrates the likely pathway for the following stages, which are then detailed throughout the remainder of this section of the protocol:

- **STEP 1**: Identification of potential participants and inviting them to participate in STRATA;
- **STEP 2:** Preliminary screening and expression of interest;
- **STEP 3:** Patient safety checks (for those who are potentially eligible in Step 2 and consent to contact GP);
- **STEP 4:** Inform individual of preliminary eligibility assessment outcome and where applicable, arrange the Baseline (first study) appointment*;
- **STEP 5 (for those who are potentially eligible in Step 4):** Conduct Baseline (first study) appointment to review eligibility, obtain informed consent and complete baseline data collection. Seek approval from the local (delegated) prescriber to proceed, and where relevant, conduct randomisation, prescribe study medication, and arrange subsequent participant follow ups.

To summarise, when potentially eligible participants are identified from any of the possible pathways (Step 1; e.g. clinical appointments and/or lists, research registers, self-referral etc.), they will be directed to a preliminary online screening questionnaire and expression of interest form (or paper copy where preferred; Step 2). This can be completed by the individual and/or the health care professional referring them to the study. The form will ask a short series of questions based on broad inclusion/exclusion criteria. If minimal requirements regarding eligibility are met, permission to contact the individual's GP (doctor) will be sought to complete patient safety checks (Step 3). Individuals for whom the study is considered suitable for them to take part, and they are interested in doing so, will be invited to attend* a Baseline (first study) appointment (Step 4). At the Baseline appointment (Step 5) the researcher will confirm eligibility, obtain informed consent, and complete outstanding baseline data collection. The researcher will then seek approval from the local prescriber (Principal Investigator (PI)/delegated clinician, or non-medical clinician such as an advanced nurse practitioner or clinical nurse specialist). Randomisation will be conducted after the prescriber is satisfied that eligibility criteria has been met and prescriptions can commence. The prescriber will prescribe study medication, and the researcher will arrange subsequent participant follow ups. Alternatively, if the prescriber believes that eligibility criteria has *not* been met and that prescriptions *cannot* commence, the researcher will notify the individual that it is not suitable for them to take part in the trial and update records, as required.

As detailed in Section 6.7 (and Appendix 2), we anticipate that the majority of Baseline appointments (and other study points of contact) will be conducted via Sponsor/NHS-approved video-conferencing (i.e. to reduce risk of potential exposure to COVID-19 and/or due to a requirement for remote contact by the potential participant). Alternative methods of communication (e.g. face-to-face visits, and/or other remote methods) will be considered and facilitated, where feasible.

NB: *Due to the potential variation in patient pathways at each centre (and subsequent sites) in the UK and Australia, arrangements will be individualised according to local arrangements in discussion with the central research team (University of Bristol [UoB]). We acknowledge that the healthcare system in Western Australia is different to that of the UK; specifically, that a Psychiatrist (rather than GP) typically oversees the care of this population group. In such cases, where GP is referred to, it is acceptable for*

suitable equivalent healthcare professionals to perform relevant assessments and/or processes. Other significant differences between the UK and Western Australia are highlighted throughout the Protocol.

STEP 1. Identification of potential participants and invitation to participate

During clinical appointment:

- Determine if the patient may be a potential participant;
- Clinician introduces STRATA* and either:
  a) proceeds to **Step 2** if individual is interested in participating; *or*
  b) provides STRATA Invitation Pack and completes a STRATA Referral Form for local Research Assistant (RA) to follow-up;
- RA completes local STRATA Screening Log.

Centre/site clinic list review:

- Identify potential participants and either:
  a) sends STRATA Invitation Pack directly to individuals; *or*
  b) Completes STRATA Referral Form and sends to local research team (e.g. RA);
- Local RA then either:
  a) sends STRATA Invitation Pack to individuals and monitors response; *or*
  b) approaches them at clinic* if nearby appointment scheduled;
- RA completes local STRATA Screening Log.

Self-referral:

- Individuals that are informed about STRATA via mailing lists or other advertising are either:
  a) sent STRATA Invitation Pack; and/or
  b) directed to study website for further information;
- If the individual is interested in participating, they proceed to **Step 2.**

STEP 2. Preliminary screening and expression of interest

Individuals who are interested in taking part complete the STRATA Preliminary Screening and Expressions of Interest Form (online questionnaire or paper version if required); this can be done by the clinician during the clinical appointment.

If responses to questions *do not* meet necessary criteria: the individual is deemed ineligible.
Instant notification given online (or soon after if paper copy completed); no further involvement.

- Central Research Team update local teams.
- RA completes local STRATA Screening Log where feasible.

XX

If responses to questions meet necessary criteria: the individual is potentially eligible.
Additional information then obtained to proceed to **Step 3**, e.g.:

- Consent to contact patient's GP (doctor) to complete patient safety checks;
- Patient contact details; and
- GP contact details.

STEP 3. Patient safety checks *[for those potentially eligible in Step 2 & consent to contact GP]*

- Central Research Team contacts GP to confirm that it is safe for their patient to take the study medication if they decide to participate; GP replies directly to patient's local research centre team;
- Central Research Team update GP Information Request Log and forward to local research centre team for monitoring.

STEP 4. Inform individual of preliminary eligibility assessment outcome

If not eligible:

- Local RA contacts individual to advise they are ineligible and thanks them for their time and interest.
- RA completes local STRATA Screening Log.

XX

If potentially eligible:

- Local RA contacts individual* to advise they are eligible and arranges the STRATA Baseline (first study) appointment.

STEP 5. Baseline (first study) appointment *[for those potentially eligible in Step 4]*

- Local RA (with patient)* will assess eligibility. Where applicable, continue to obtain informed consent and complete baseline data collection. Randomisation will be conducted after the local (delegated) PI/prescriber is satisfied that eligibility criteria has been met and prescriptions can commence.
- RA confirms outcome with participant, and where relevant, arranges subsequent follow up contact and provides the STRATA Welcome Pack. In all cases, the RA then completes local STRATA Screening Log.

Denotes timepoints to (audio) record discussion with patient after seeking separate consent, as per study protocol (see Sections 6-7 for further details).

Figure 3 Identification and screening of potential participants

6.3 STEP 1: Participant identification and invitation to participate

Likely pathways to identify potential participants include: (a) clinical appointments and/or clinical lists; (b) research registers/cohorts; and (c) other methods, including self-referrals. For each of these, arrangements should be suitably established according to local practice, in discussion with the central research team (UoB). Centres/sites may also identify other opportunities and methods for identifying individuals and inviting them to take part, which should be utilised.

6.3.1 Clinical appointments and/or lists

Potential participants will be identified within the trial centres/sites by clinical services staff, CRN or Research and Development (R&D) department staff. These can be done within the context of a clinical appointment or by review of clinical lists. Where possible, GAD-7 screening will be embedded routinely in recruiting clinical sites and the service staff listed above will identify individuals with a diagnosis of autism who score ≥10 on the GAD-7. These patients will be suitable to be referred to the study/local centre research team, who can then liaise with the individual. Services may also refer patients who have a diagnosis of autism who may be seeking support for their anxiety.

Within clinical appointment: Service staff may discuss the study with individuals during a consultation and either proceed to preliminary screening (Step 2) or provide them with a STRATA Study Invitation Pack (paper/online link, as described below) and refer them to the local research team for further liaison. To do so, staff identifying potential participants should complete a STRATA Referral Form so that the local research team (e.g. centre research assistants [RAs]) can update and monitor their local centre STRATA Screening Log.

By review of clinical lists: Individuals identified as potential participants will be sent, by email or post, a STRATA Study Invitation Pack; this pack will contain a study approved invitation letter (or email equivalent), a Participant Information Leaflet (PIL) including contact details of the local centre research team, and instructions of what to do if they would like to find out more information or are interested in taking part (i.e. complete an online STRATA Preliminary Screening and Expression of Interest Form, or, if applicable, paper equivalent using a pre-paid envelope [see Step 2]). Alternatively, they may be directed to the study website containing an equivalent Study Invitation Pack for online review if preferred.

Central research staff (UK/Western Australia, respectively*) will monitor completion of the Preliminary Screening and Expression of Interest Form. Where feasible, local (recruitment) research staff will contact individuals who were sent materials but not yet responded, to see if they would like to take part. If so, they will be directed to the online (or paper equivalent) STRATA Preliminary Screening and Expression of Interest Form; see Step 2, below. Alternatively, if a known clinical appointment is pending (within 8-weeks of the invitation being sent), local research staff could arrange to meet at the next clinical appointment and discuss the study face-to-face; combining an existing clinical appointment avoids individuals additional time/contact, as well as provides face-to-face discussion, if preferred by the individual.

**Central UoB research staff will monitor and are responsible for UK responses and related activity, and the equivalent team in Western Australia will monitor and are responsible for Australian responses and activity. This applies here, and throughout this section.*

NB: *In light of COVID-19, we are mindful that face-to-face appointments bring about higher risk and may not be feasible. To mitigate such risks, STRATA offers flexibility around assessment and communication methods as noted throughout (and see Appendix 2). If face-to-face appointments are requested, research*

staff should assess feasibility and follow the organisation guidance at the time regarding face-to-face contact, including, for example, use of personal protective equipment (PPE) and cleaning procedures.

QuinteT Recruitment Intervention (QRI) [UK only]: At the earliest opportunity in the study pathway, individuals will be asked if they agree to the (audio) recording of conversations about the study between themselves and a clinician/member of the research team to understand and optimise the trial recruitment process (see Section 7, QRI, for further details); Steps 1, 4 and 5 of the pathway presented in Figure 3, present the most likely opportunities to incorporate the QRI (recordings). The aim is to record all conversations in which the study is discussed up until a decision on study participation is made.

Based on the opportunities and methods presented in Step 1 above, for identifying individuals and inviting them to take part, conversations that would be particularly valuable to record at this stage, where feasible, include: (i) within the clinical appointment when services staff discuss the study with individuals during a consultation; (ii) by review of clinical lists when research staff contact individuals who were sent materials but not yet responded to see if they would like to take part; and (iii) by review of clinical lists and a known clinical appointment is pending and research staff arrange to meet at the pending appointment.

Screening logs: Local research staff (e.g. RAs) should complete a STRATA Screening Log, which will be developed in line with the SEAR (Screened, Eligible, Approached, Randomised) framework (47); this framework will enable us to record the flow of potential participants through the recruitment process, in line with recommended Consolidated Standards of Reporting Trials (CONSORT) reporting guidelines (48). Where possible, screening logs will include reason(s) for non-participation. This will ensure that they are not approached more than once, as well as highlight those who are willing to be contacted again in the future (e.g. they were not able to participate when first approached but may be in the future, such as individuals prescribed serotonergic antidepressant/ anxiolytic in preceding 8-weeks, but could do so after a washout period of 8-weeks). Local research teams will enter screening log data directly into the relevant STRATA database (or via alternative data capture forms, if necessary); these will be monitored regularly by the central research team (UoB).

It is acknowledged that completion of screening logs will not be possible for some potentially eligible participants who receive information about the study, e.g. where a large number of individuals receive information via a newsletter.

6.3.2 Research registers / mailing lists

Where relevant, we will seek approval from the steering committee (or equivalent) of relevant research registers/mailing lists, in both the UK and Western Australia, to inform individuals about STRATA. For example, these include, the Adult Autism Spectrum Cohort - UK (AASC-UK) (49); Discover Network (Autistica Charity, UK) (50); and the Western Australian Register for Autism Spectrum Disorders (51). Other relevant registers/mailing lists that are not listed here may also be approached.

Potential participants who are registered on such a register/mailing list will be sent information about the trial. This may be via an email/postal newsletter which directs interested individuals to the study website for further information, or by providing a STRATA Study Invitation Pack (as described above, Section 6.3.1).

Western Australia Autism Register: For some individuals registered on the Western Australian Autism Register, the contact information will only be available for the potential participant's parent or guardian. The parent/guardian may have given consent for their child to be registered at the time of

diagnosis when the child was less than 18-years but are now of adult age. In these cases, the parent/guardian will be sent the information to forward accordingly, so that the individual can decide if they wish to express an interest in participating.

6.3.3 Other methods and organisations

Approved STRATA recruitment materials (e.g. posters, leaflets, electronic animation/video and/or equivalent materials) may also be displayed in relevant locations, providing information about how an individual can obtain further information/take part, including relevant contact details. Equivalent information may also be promoted via relevant social media resources (e.g. twitter, websites).

UK: Using similar approaches as noted above, we will engage with the NIHR CRNs which cover the UK recruiting centres to disseminate the opportunity to participate in STRATA to members in their regions. We will also engage with various community organisations including charities and third sector providers of support services and university disability services. Information (e.g. approved posters and leaflets or emails with study weblink) about the study may be sent to local organisations, University disability services and GP surgeries to supplement recruitment from these sources.

Western Australia: If additional recruitment is required in Western Australia, members of the Western Australia Autism Diagnosticians Forum who are involved in adult diagnoses, and/or community support organisations, will be approached and encouraged to disseminate information about the study and direct potential participants to the Perth research team(s) for further information and assessment, where relevant.

6.4 STEP 2: Preliminary (eligibility) screening and expression of interest

When potentially eligible participants are identified and interested in taking part, they will be directed to complete a STRATA Preliminary Screening and Expression of Interest Form. The form can be completed via a secure online weblink, or via paper equivalent if required/preferred; it can either be completed directly by individuals and/or by clinicians wishing to refer individuals to the study (as noted in Section 6.2).

The aim of this form is to ask individuals/clinicians a short series of questions based on broad inclusion/exclusion criteria, as a means of preliminary screening (eligibility assessment). For example, questions will include (at least): GAD-7; confirmation that the individual has received an autism diagnosis; willingness to consider medication for anxiety, presence of intellectual disability; other broad inclusion/exclusion criteria; and basic demographic information (e.g. year of birth; gender; ethnicity; employment status; highest level of qualification; marital status). Broad information about their geographical location will also be collected (e.g. what their local city is), so to align the individual with a relevant research centre.

Name and contact details will be collected in both online and paper versions of the forms; these will be necessary to enable the study team to contact the potential participant to inform them about their eligibility status and to ensure that inadvertent typos can be clarified (e.g. the webform calculating their age as 0 years if they note their year of birth as the current year in error). Personal details will only be held for the minimum required time. The expression of interest form will therefore be presented in full in both paper and online versions.

If responses to preliminary screening questions do not meet the necessary trial criteria: the individual is deemed ineligible for this trial and will be notified of this outcome. Notification will follow completion of questions via the online form, or by subsequent contact for those completing a paper equivalent. No further data about the individual will be collected. The central research team

(UK/Western Australia, respectively) who will be responsible for processing these forms (online and paper), will update local research teams so they can update their local screening logs where feasible.

If responses to preliminary screening questions meet the necessary trial criteria: the individual is potentially eligible to take part, and additional information, if not already available, will be requested to conduct further assessment. This additional information will include (at least): the individual's name, date of birth, address, and other contact details (e.g. telephone and email address') and preferences; consent of the individual to contact their GP (doctor) or to obtain medical summary for patient safety checks*; and GP contact details (e.g. GP name, if known, and practice contact details).

The central research team (UK/Western Australia, respectively) will monitor completed forms and proceed to Step 3, as outlined below. At this point, individuals will be allocated a unique study identification (I.D) number.

** Due to the nature of this trial, it will not be possible for an individual to participate if the research team are unable to obtain essential patient safety check information from the individual's GP (or equivalent healthcare professional in Western Australia, for example).*

6.5 STEP 3: Patient safety checks (for those who are potentially eligible in Step 2 and consent to contact GP)

The local research centre teams (or central research team in UK/Western Australia if required) will contact the GP practice, or equivalent health care provider in Australia, of those who are considered potentially eligible to take part in the study and have provided consent for their GP to be contacted for patient safety checks. Using secure methods of contact (e.g. via NHS.net email, telephone and/or post), the GP practice will be provided with relevant information about the trial and asked to confirm that it is safe for their patient to take the study medication if they decided to take part (i.e. complete a study specific GP Patient Safety Check Form or return a GP Patient Medical Summary, which should be returned directly to the individual's local research centre team). If the local research centre team already has access to medical records held for the individual (e.g. on SystmOne) then a medical summary can be obtained without contacting the GP practice directly. The individual's local research centre team will then confirm provisional eligibility status.

Local research centre teams should use a GP Patient Safety Check Log informing them which GPs have been contacted, for which individual, and when; local research centre teams are responsible for monitoring GP responses for their potential participants, reminding as required, updating screening log records, and proceeding to Step 4, below.

In the event that GP safety check responses are delayed (i.e. between Step 2 and Step 4 of the recruitment pathway), the local research centre teams will aim to send potential participants 'holding' emails and/or text alerts, based on identified contact preferences; this contact is to help retain the engagement of, and provide assurance to, potential participants.

6.6 STEP 4: Inform individual of eligibility assessment outcome and arrange the Baseline (first study) appointment, where applicable.

Local research centres will contact individuals to inform them of their preliminary eligibility assessment outcome.

For those confirmed as ineligible (not able to participate): they will be sent an approved STRATA Eligibility Assessment Outcome Letter (or email equivalent) informing them they are not eligible (at this time) to participate. Within the letter they will also be reminded that their medical treatment will

continue as usual with their GP, as will any other services they are receiving. They will be thanked for their time and interest in the trial.

For those confirmed as potentially eligible (able to participate): they will be informed that they are potentially eligible and asked to confirm that they still wish to participate; this notification may be via an approved letter or alternative methods (e.g. email/video-conferencing/phone) depending on the individual's contact preferences and/or availability. As noted previously, where feasible throughout Step 4, the researcher should ask individuals if they agree to the (audio) recording of conversations about the study, i.e. incorporate the QRI (UK only).

If they still wish to participate, the research staff will invite them to attend a Baseline (first study) appointment at a convenient time (and location), making note of any accessibility needs or reasonable adjustments to be made to facilitate this appointment. The researcher will inform individuals that prior to the appointment (no more than 3-days in advance), they will receive the STRATA Baseline Questionnaires via secure online link(s) (or hard copy* can be posted upon request, which will include clear instructions about when to complete and return the questionnaire booklet). If either of the questionnaires have not been completed (and/or hard copies returned) by the time of appointment, they can be completed during the Baseline appointment. The purpose and details of the Baseline appointment are outlined in Section 6.7, below.

**The Revised Clinical Interview Schedule (CIS-R) is only available as a computer-based (online) assessment. All other questionnaire outcome measures will be incorporated into one STRATA Baseline Questionnaire, which will be available online or in hard (paper) copy if required.*

If for any reason the individual has changed their mind and no longer wishes to participate, for research monitoring purposes, the researcher will: (1) ask 'why', however the patient does not have to provide a reason if they prefer not to; (2) update the local screening log accordingly; and (3) confirm the individual is happy for the research team to retain any anonymised data collected. Where applicable, all data obtained with reference to the QRI/integrated qualitative research, however, will be retained according to the relevant patient information and separate consent.

6.7 STEP 5: Baseline (first study) appointment (for those potentially eligible in Step 4)

Figure 4 (next page) illustrates the key elements of Step 5, as detailed throughout this sub-section.

Potential participants will attend a Baseline (first study) appointment*. On commencement/arrival they will be asked if they agree to the (audio) recording of this discussion about the study (see Section 7, QRI), and the aim of the appointment will be explained. At this appointment, eligibility criteria will be reviewed for any changes since Step 3 (or last contact), provisionally confirmed, and the trial explained; individuals will be able to ask any questions.

During this baseline discussion, the researcher will check that individuals are fully informed about the study by asking them to: (a) summarise their understanding of what participating in the study will involve; (b) enquire about the voluntary nature of their involvement; and (c) ask what will happen if they no longer wish to take part. This will enable the researcher to check that they have understood and retained key aspects of the information provided about the study and are aware of the voluntary nature of their involvement and their right to withdraw. Furthermore, patients will enter this study with the understanding that any benefits of sertraline may take several weeks to appear, and that the treatment regimen will last one year (52-weeks). Potential participants will be informed that they should take the treatment regimen for at least 16-weeks (i.e. until the primary outcome timepoint)

before deciding if the treatment works or not, although they may withdraw from the medication and/or the study at any time, should they wish to. Further details about dosing schedule are in Section 9.

**As previously noted (and outlined in Appendix 2), we anticipate that the majority of Baseline appointments (and other study points of contact) will be conducted via Sponsor/NHS-approved video-conferencing (i.e. to reduce risk of potential exposure to COVID-19 and/or due to a requirement for remote contact by the potential participant). Alternative methods of communication (e.g. face-to-face visits, telephone, email and/or other remote methods) will be considered and facilitated, where feasible.*

If face-to-face appointments are requested, research staff should assess feasibility and follow the organisation guidance at the time regarding face-to-face contact, including, for example, use of personal protective equipment (PPE) and cleaning procedures.

Discussion & Eligibility Review

- Potential participant attends Baseline (first study) appointment; on commencement/arrival ask if they agree to the audio-recording of discussions about the study (see Section 7, QRI). The aim of the appointment will be explained.
- Eligibility criteria reviewed for any changes since Step 3 (or last contact), provisionally confirmed by the researcher, and trial explained in more detail; opportunity for individuals to ask any questions. Researcher checks that individual is fully informed about the study by asking them various pre-specified questions.

Informed Consent

<u>**If researcher is not satisfied that full eligibility criteria has been met, or where individual declines to take part:**</u> do not proceed to take informed consent for the main trial (see Section 6.7). Complete local **STRATA Screening Log**.

XX

<u>**If researcher is satisfied that full eligibility criteria has been met:**</u> proceed to take informed consent for the main trial (see Section 6.7.1).

Data Collection

Complete **Baseline Data Collection** as identified in Table 3.
For example (see Section 6.7.2):

- Baseline Questionnaires (including online/computer-based CIS-R)
- Baseline Case Report Form

PI (prescriber) Approval

Seek review and approval from local "prescriber" (e.g. local Principal Investigator (PI) or authorised delegate) (see Section 6.7.3).

<u>**If "prescriber" is not satisfied that eligibility criteria has been met and prescriptions can commence:**</u> notify individual and update study records, including local **Screening Log** and **STRATA Change of Permissions / Withdrawal Form**.

XX

Randomisation & Prescription

<u>**If "prescriber" is satisfied that eligibility criteria has been met and prescriptions can commence:**</u> (see Sections 6.7.3 & 6.7.4)
(1) proceed with randomisation and prescribing study-medication;
(2) notify individual and arrange 'Brief Safety Check' follow ups; and
(3) provide **STRATA Welcome Pack**.

End of Baseline / Post-Randomisation

Once final recruitment outcome of the individual is known, (i.e. randomised or not), the local research staff should (see Section 6.7.5):
(1) update local **Screening Log** with final screening and recruitment outcome;
(2) data enter any paperwork to study database (where applicable) and file all paperwork; and
(3) <u>[only If individual is randomised (enrolled)]</u> notify the participant's GP (or equivalent healthcare provider in Australia) that their patient is taking part in this trial, using a study-approved **STRATA GP Notification Letter**.

Figure 4 Overview of key elements within STEP 5: Baseline (first study) appointment

<u>Once the researcher is satisfied that full eligibility criteria has been met:</u> they can proceed as outlined, below. At this timepoint in the recruitment process, potential participants will have been given a chance to ask questions and have had more than 24-hours (after receiving the trial PIL) to think about taking part, before providing informed consent and being randomised.

<u>Those who do not meet the full eligibility criteria, or those who are eligible but decline to take part:</u> in such cases the researcher will not proceed with taking informed consent, nor enrolling in the study. If the individual completed a baseline questionnaire prior to this visit, the researcher will securely destroy these data (in accordance with Sponsor requirements). The researcher would offer the individual a £10.00 gift voucher ($20 in Australia) to thank them for their time (see Section 6.10) but no further appointments will be made regarding this study, unless the individual consented on the (audio) recording consent form to be contacted by a study researcher for an interview to explore their views on the study.

6.7.1 Consent (main trial)

Where applicable, delegated clinical staff at sites who are trained on the STRATA protocol and procedures and have relevant experience, will be able to take informed consent. This will include local research associates/assistants, clinicians and CRN staff within each centre/site.

Within the consent process, consent will also be sought for future re-contact and sharing of their anonymised data for other ethically approved studies. We will also seek consent for linkage to central NHS records (e.g. NHS Digital linked data and equivalents for UK-patients, or equivalent electronic health records for patients in Western Australia), as a possible mechanism for longer-term follow up, as requested in the original NIHR HTA funding application call; longer-term follow up is *not* included within this application and protocol and would be applied for separately if relevant.

To enable remote and/or face-to-face methods of contact, where feasible, informed consent will be captured via a Medicines and Healthcare products Regulatory Agency (MHRA) (UK) and NHMRC & TGA (Australia) compliant eConsent (online) form and process. An approved paper (wet ink) equivalent will be available where eConsent is not feasible. Further guidance will be provided in study-specific training materials. Four copies of the completed consent form are required:

1) a copy must be filed in the Investigator Site File (ISF) together with a copy of the PIL in recruitment order*;
2) a copy should be provided to the patient*;
3) a copy should be placed in the patient's medical notes with a supporting record of the discussion and a copy of the PIL**; and
4) a copy should be provided to the STRATA central research team.

**If a paper (wet ink) form is completed, the 'original' form should be filed in the ISF. If an eConsent form is completed, a completed copy is automatically emailed to the patient once processed, and additional copies can be obtained via the eConsent (database) system.*

***Besides completing the consent form, centres/sites should record key details of the informed consent process in the patient's medical notes (where feasible); study title and date of consent are on the consent form. Patients are not required to provide reasons for not taking part in the study, but if they are given, then they should also be documented in their notes. For non-NHS recruitment sites without direct access to patient medical notes, a copy of the consent form and supporting information should be sent to the patient's GP alongside the GP Notification letter.*

Consent for the QRI and integrated qualitative research are discussed separately; see Section 7.2.

6.7.2 Further baseline assessments

Questionnaires (assessments): Following consent, if the necessary baseline assessments identified in Table 3 have not yet been completed (and/or hard copies of the questionnaire not returned), they should be completed during this appointment*. Individuals will be offered breaks during this appointment to minimise fatigue and burden. If they require assistance to complete the questionnaires, the researcher can facilitate this. Similarly, a carer/family member or friend can also join the appointment to provide support. The research team will aim to try and make all reasonable adjustments requested by the participant to facilitate this appointment.

**The Revised Clinical Interview Schedule (CIS-R) is only available as a computer-based (online) assessment. All other questionnaire outcome measures will be incorporated into one STRATA Baseline Questionnaire, which will be available online or in hard (paper) copy if required.*

Case Report Form (CRF): The researcher will also complete a STRATA Baseline CRF to capture (or confirm if previously collected) relevant information. CRF content will include (as a minimum): eligibility assessment (as per Section 5, plus discussion points noted in Section 6.7); presence of ID and relevant supporting information; details of autism diagnosis, including when, where and what; contact details, including back up details (e.g. carer/ other family members); contact preferences; any requirements for future correspondence/appointments; other essential information to support randomisation stratification and minimisation variables; and medication dispensing preferences.

6.7.3 Next steps

At this point, the researcher has completed the eligibility assessment/review, informed consent and baseline data collection, thus deeming that it is suitable for the individual to take part in this trial, and they are willing to do so. It is essential that the researcher now seeks approval to proceed to randomisation (enrolment) and prescription of study medication from the local "prescriber" (e.g. local PI or authorised delegate)*.

If the prescriber is satisfied that eligibility criteria has been met and prescriptions can commence: they (the prescriber and/or researcher) will proceed with randomisation (as detailed in Section 6.7.4, below) and the prescriber will complete and submit the study-specific prescription to initiate the study medication dispensing process. The researcher will update the (now) participant and arrange the forthcoming 'Brief Safety Checks' as detailed in Section 6.8.1. As advised by our patient advisory group, arrangements should include: confirming the date and times of the follow ups; confirming participant contact details, including methods of contact and any other preferences; and providing the researcher's contact details, including any relevant telephone numbers/caller I.Ds/email address', where applicable. This process allows the participant to plan, knowing who to expect contact from, when and by which method.

The participant will then be provided with additional information and materials in the form of a STRATA Welcome Information Pack, which will either be posted, or provided in person (depending on how the appointment is conducted). Some of the information may also be available via email, or downloadable via the study website. The STRATA Welcome Information Pack will include (as a minimum):

a) Information about taking the medication (STRATA Medication Instructions);

b) a STRATA Alert Card indicating that they are participating in the STRATA RCT, which participants can show to any health professionals involved in their care;

c) a STRATA Carer Study Information Pack**; as detailed in Section 8, we plan to conduct a nested study exploring carer burden; and

d) a STRATA Week 1 to 2 Safety Check Questionnaire booklet and pre-paid envelope.

If the prescriber is not satisfied that eligibility criteria has been met and that prescriptions can commence: the researcher will notify the individual that it is not suitable for them to take part in the study and update study records, as required. This includes: completing a STRATA Change of Permission / Withdrawal Form (e.g. Patient declined/was not randomised after giving informed consent); and securely destroying relevant data, e.g. baseline questionnaires and CRF(s) (in accordance with sponsor requirements). A copy of their consent form, however, should be retained alongside the Change of Permission / Withdrawal Form.

**In some circumstances it may be feasible to seek prescriber approval ‘there and then’ (i.e. in a clinic setting). It may, however, need to take place separately (i.e. if appointments are conducted remotely, or the local prescriber is not available); in such cases, the researcher should explain this to the individual (patient), making clear that they will notify them of the outcome as soon as available. This notification may be via telephone/video-call or an approved letter (or email equivalent), depending on participant contact preferences and/or researcher availability.*

***If the patient (individual) has confirmed that they have a carer, the researcher should provide them with a STRATA Carer Study Information Pack. The individual will be asked to forward this to their carer at the earliest opportunity, on behalf of the STRATA research team.*

6.7.4 Randomisation

Individuals will only be randomised after: (a) safety checks are completed by their GP; (b) written informed consent (wet ink or eConsent) has been obtained; (c) baseline assessments have been completed; and (d) eligibility is approved by the local recruiting site PI, or authorised delegate (e.g. “prescriber”).

The randomisation sequence will be generated by Sealed Envelope^{TM} (52). Randomisation will be stratified by centre, with minimisation to ensure balance in baseline GAD-7 score (<15 and ≥15), gender (male, female, non-binary), age (18-34, 35-49 and ≥50), presence of intellectual disability (yes/no), and previous medication use for anxiety or depression (yes/no). Patients will be randomised to one of two treatment groups on a 1:1 ratio, that is either sertraline (intervention arm) or placebo (control arm).

The local PI (or authorised delegate) will sign into the secure online randomisation system, enter the individual’s (patient’s) unique study I.D number (previously allocated in Step 2; see Section 6.4) and necessary minimisation variables; they will then receive the code that allocates the participant to the study treatment, and this code should be recorded on the study-specific prescription (see Section 9.6 for Prescribing Procedure). The recruiter(s) and the participant will remain blinded as to which treatment group this code refers to. The trial pharmacies in the UK and Australia will be directly informed of the randomisation code for UK and Australia-based patients, respectively. The unblinded randomisation code will be held by the study pharmacies and selected members of the Bristol Trials Centre (BTC; see Sections 6.14 and 12.1). Details of the study medication, including dispensing and dosing are provided in Section 9.

6.7.5 End of Baseline appointment / post-randomisation requirements

Following completion of the Baseline appointment (once final recruitment outcome of the individual is known, i.e. randomised or not), the local recruiting centre/site staff should:

1) update their local screening log with the relevant screening and recruitment outcome;

2) data enter any paperwork to the secure online study database (where applicable) and file all paperwork securely (see Section 12, Data Management); and

3) ***<u>Only if individual is randomised (enrolled)</u>:*** notify the participant's GP (or equivalent healthcare provider in Australia) that their patient is taking part in the STRATA trial, using a study-approved STRATA GP Notification letter.

6.8 Follow-up assessments

6.8.1 Brief contact (safety checks): 1 to 2-, 4-, 8-, 12-, and 36-weeks post-randomisation

At 1 to 2-, 4-, 8-, 12- and 36-weeks post-randomisation*, the local research (recruitment) team will contact the participant to conduct** a brief safety check to assess safety (adverse effects), medication dose titration, and anxiety and depressive symptoms (including suicidality). This assessment will be carried out via questionnaire and discussion to adequately inform dose titration.

For these assessments, participants will be asked to complete the questionnaire element online (unless alternative method is requested), followed by a video call (using Sponsor/NHS-approved video-conferencing tools) or telephone call by the researcher. If the participant has not completed the questionnaire by the time of the researcher call, the researcher can help complete it during the call.

The researcher will, in a timely manner, convey this assessment to the local PI ("prescriber", or authorised delegate) for them to review and make the decision regarding further prescription (continue on same dose, increase or decrease). If required, the PI/clinician can arrange to speak with/or arrange to see the participant directly or request further information to enable their decision. The decision to prescribe and amend dosage shall stay with the PI/clinician and not the RA who is responsible for conveying the relevant information to the PI/clinician.

** The initial safety check at 1 to 2 weeks should be at least 1-week, but no later than 2-weeks, post-randomisation. We anticipate the need for flexibility of +/- 1-week given the pragmatic nature of this trial for the safety checks at 4-, 8-, 12- and 36-weeks post-randomisation.*

***Given the timings of these safety check assessments, face-to-face appointments are unlikely to be feasible or preferable to participants and will only be considered in exceptional circumstances, if possible. Participants will be given a choice of remote method(s) of contact and assessment, e.g. video call (using Sponsor/NHS-approved video-conferencing tools), email, text links to complete questionnaires online, and/or telephone.*

It is important to note, however, that medication dosage <u>must</u> be discussed with research staff rather than via online questionnaire, only. Where safety is a concern, participants will be encouraged to seek input from their clinician/GP to help with clinical management.

<u>Questionnaire (participants):</u> Participants will complete a STRATA Safety Questionnaire at each timepoint; the questionnaire will contain patient-reported outcome measures (PROMs) and additional data collection as presented in Table 3. Local research staff/teams are responsible for the conduct and monitoring of these follow ups.

<u>CRF (centre staff):</u> Delegated local (recruitment) centre staff will complete a STRATA CRF at each timepoint. CRF content will likely include (as a minimum):

- Adverse event reporting;
- Whether the participant is happy to carry on taking the study medication;

- The participant's view on whether they would like to stay on the same dose, try an increase or would like a reduced dose of the study medication.

IMPORTANT NOTE: At the 1 to 2-week safety check the research team will confirm that the participant has received their study medication, and that they have started taking it (or intend to take). It is possible, however, that between agreeing to take part and receiving the medication, the participant changes their mind and is no longer willing to take the study medication. In such cases, the researcher will ask the participant to return the study medication (as instructed) to confirm that the study medication is untampered (i.e. there are no capsules missing or opened, thus study treatment has not commenced).

If it is confirmed that no medication has been taken, then the individual should be classified as a "screening failure", rather than "participant withdrawal". The research team will need to:

1) update their local screening log with the relevant screening and recruitment outcome;

2) notify the individual's GP (or equivalent healthcare provider in Australia) that their patient is no longer taking part in the STRATA trial, using a study-approved STRATA GP Update of Involvement Notification letter; and

3) update study records, including completing a STRATA Change of Permission / Withdrawal Form (e.g. Patient did not commence study medication after giving informed consent). No further data collection (follow ups) should take place for this individual.

If the participant fails to return medication and it is not possible to establish that the medication is untampered, then they will continue to be counted as a "trial participant" (enrolled). Usual change of permissions/withdrawal procedures will apply.

6.8.2 Follow up assessments: 16-, 24- and 52-weeks post-randomisation

Follow up questionnaires (participants): Participants will be asked to complete a STRATA Follow Up Questionnaire at each timepoint; each questionnaire will contain PROMs and additional data collection as presented in Table 3. Participants will be asked to complete the questionnaires online and will receive a secure online link at the appropriate timepoints. Alternative methods preferred by the participant will be considered and facilitated where feasible (e.g. by video call (using Sponsor/ NHS-approved video-conferencing tools), postal hard copy, face-to-face, or telephone). If the participant requires assistance to complete the questionnaires, the research team will aim to try and make all reasonable adjustments requested by the participant to facilitate this. Similarly, a carer/family member or friend can provide support, but they will be advised not to answer any questions on behalf of the participant.

Local research staff/teams are responsible for the conduct and monitoring of these follow ups. If they do not receive a response from a participant within a reasonable time of sending each STRATA Follow Up Questionnaire (e.g. ~3 weeks), then they will contact them, and/or resend another link/copy with a reminder letter/equivalent contact. For each follow up, the research team will make three contact attempts in total (initial sending, plus two reminders). If no response is received after the third attempt, the relevant questionnaire will be marked as missing. We will, however, continue to send the next follow up questionnaire as planned, unless the participant requests/confirms that they no longer want to complete them; a similar model has been successfully used in multiple studies conducted by the BTC.

Included with each questionnaire (initial contact and reminders, where applicable) will be information that contact with the participant is still essential for further supplies of medication even if they do not wish to complete the questionnaire; clear instructions will inform the participant that if they wish to discontinue taking the medication, they should contact their local research team so that support regarding down titration is given. Therefore, participant contact need not be the completed questionnaire, but confirmation (verbal/written) that they are still taking the medication and wish to continue, is essential. If in doubt (e.g. if no contact is received from the participant), the local research team should call (email/text or equivalent) to check; if considered necessary at this point, the researcher could utilise a back-up 'best contact' (e.g. carer/ other family member, where provided). By the time of the next supply, if no contact has been made, the local research team will complete an Investigational Medicinal Product (IMP) Discontinuation Form to ensure that new medication is not supplied to the participant for safety purposes. This should also be recorded in the relevant timepoint CRF.

The 52-week post-randomisation follow up (questionnaire) is the final timepoint and marks the end of treatment. The same reminder system noted above will also apply here, where applicable. In addition to this questionnaire, participants will be provided with information about tapering down and stopping the medication. At this final timepoint, researchers will make 'a brief exit call' to explain to participants what will happen next; where feasible, this should be combined with other discussions (e.g. when completing the questionnaire via video/telephone, and/or reminders contact). Alternatively, it could be made after receipt of a completed questionnaire.

Further details about prescribing and medication dispensing are provided in Section 9.

Follow up CRFs (centre staff): Delegated local (recruitment) centre staff will complete a STRATA Follow Up CRF at each timepoint. CRF content will include (as a minimum):

- Adverse event reporting;
- Whether the participant is happy to carry on taking the study medication.

6.9 Qualitative assessments

The QRI and integrated qualitative research are detailed in Section 7, below. Table 3 also depicts timings of data collection.

6.10 Thanking participants for their involvement (payments)

Upon receipt of completed questionnaire booklets at Baseline, 16-, 24- and 52-weeks post-randomisation (or at least provision of key data, e.g. GAD-7, PHQ-9 and EQ-5D-5L), the central research team (UK and Western Australia, respectively) will offer participants a £10.00 gift voucher ($20 in Australia) (i.e. £10.00/$20.00 per questionnaire, up to £40.00/$80.00 in total per patient/participant over the 52-weeks duration of the study). Participants will also be sent newsletters telling them about the study, including progress and results once available. Section 16 provides further details about dissemination.

6.11 Methods/ procedures to protect against other sources of bias

6.11.1 Loss to follow up (attrition bias)

We have had extensive consultation with our advisory group and incorporated various suggestions to ensure the study is acceptable to potential participants. We will take active measures to minimise loss of participants from the trial in line with ethical and regulatory approval. This may include, for example:

- reminders to participants developed according to individual preference (e.g. post/ email/ video call (using Sponsor/NHS-approved video-conferencing tools)/ text/ telephone);
- ability to complete questionnaires via their preferred method (e.g. online/ post/ telephone/ video call (using Sponsor/NHS-approved video-conferencing tools)/ face-to-face);
- obtaining back-up 'best contact' addresses (including carer/ other family member, where applicable);
- contacting their GP (practice) to check their contact details on record are still valid; (53) and
- using vouchers as retention incentives (54).

In addition, we may access centrally-held health data, for example via the NHS Strategic Tracing Service in England and Wales, and WebPAS in Western Australia, to find new addresses.

We have extensive experience of using the above strategies and measures and have received Ethics approval to do so in previous studies.

6.11.2 Measurement bias

Validated questionnaires for PROMs will be used to minimise the possibility of measurement bias.

6.11.3 Other sources of bias (detection bias)

Treatment allocation will be concealed from participants and their clinical care team during the trial. Research staff will also be blinded to allocation while conducting data collection for outcomes and performing data entry, and by using study I.D numbers only to identify participants, questionnaires and other documentation.

All analyses will be clearly pre-defined in the Statistical Analysis Plan (SAP), which will be reviewed and approved by the study TSC and made publicly available prior to the end of follow-up to avoid bias.

6.12 Usual care

This is a pragmatic trial and usual care will continue without restriction, including referrals to psychological therapies, such as IAPT services (29). GPs/clinicians can also prescribe other medication as necessary but will be asked to exercise caution in case they plan to prescribe drugs that may interact with sertraline.

<u>Contra-indicated medications and/or cautions:</u> A list of contra-indicated medications and/or cautions can be found in the supporting Summary of Product Characteristics (SmPC); SmPC for "Crescent Sertraline 50mg film-coated tablets, date of revision of the text 23rd September 2019".

6.13 Emergency contact procedure for participants

Details of what a participant should do if they experience any problems or side effects whilst taking part in the trial are detailed in the STRATA PIL and study medication instructions. The trial data collection schedule (Table 3) includes reporting of adverse events at all contacts. Participants will be provided with contact details of their local research centre/site team and for the central research team (University of Bristol for UK-participants or The University of Western Australia for Australian participants).

If a symptom is troublesome (as explained in the PIL) participants are advised to seek medical help in the normal way (e.g. UK via dialling 111 or contacting their GP, or Australia via dialling Health Direct on 1800 022 222, or calling the Mental Health Emergency Response Line on 1300 555 788, or

contacting their GP). In an emergency they should phone the emergency services or attend an Emergency Department. The central/local research team will only *advise* a participant action to take with respect to the investigational medicinal product (IMP) and will not provide any other medical advice.

Each participant will be given a card to carry (STRATA Alert Card) indicating that they are participating in the STRATA RCT, which they can show to any health professionals involved in their care.

6.14 Blinding

The central research team, clinicians, other researchers, site staff and participants will be blinded to the allocation of treatment group, except for one of the two trial statisticians and data manager (UoB-UK) and trial pharmacists (UK and Australian for their respective participants). Two statisticians based at the University of Bristol (UoB) will support this trial. The senior (lead) statistician will be blinded throughout the trial. The second trial statistician will perform all disaggregated analyses according to a pre-specified SAP and will attend closed Data Monitoring Committee (DMC) meetings as required. In addition, the health economist(s) (UoB) will be blinded when cleaning data and preparing the analysis plan, but unblinded when conducting the analysis.

6.15 Unblinding

<u>Central research team(s):</u> Treatment codes will only be released to the investigative team once written confirmation has been received that the trial database has been locked. The central pharmacy in the UK and Australia will then send the relevant central research team (UK or Australia) a list of all participants and their treatment allocation. Any incidents of unblinding before the trial database has been locked will be recorded by the central research team.

<u>Participants:</u> Participants will be given the option of unblinding after their involvement with the study has ended, so that they can contact their usual care provider and seek to continue/initiate sertraline treatment. Alternatively, those who withdraw from taking the medication or the study will be given the option of unblinding at the time of withdrawal or the 16-week primary outcome - whichever comes later.

<u>GPs:</u> Participant’s GPs (or equivalent healthcare professionals in Australia) will also be notified when relevant.

6.16 Emergency unblinding

In the event of a medical emergency the participant’s treating doctor can contact the relevant central pharmacy who will hold the treatment allocation and will be available 24/7; see details directly below: The UK-central research team (UoB-UK) should be notified if emergency unblinding occurs.

<u>UK:</u> University Hospitals Bristol and Weston NHS Foundation Trust (UHBW)

The direct number for UHBW Pharmacy trials, which is manned 08:30-17:00 Monday to Friday, is +44 (0)117 342 4175. Outside these hours, call the UHBW hospital switchboard on +44 (0)117 923 0000 and ask for the “on-call pharmacist”.

<u>Australia:</u> Fremantle Hospital (FH) (Fiona Stanley Hospital Fremantle Group (FSHFG)) Pharmacy

The direct number for FH Pharmacy trials, which is manned 08:30-17:00 Monday to Saturday (except public holidays), is +61 (0)8 9431 2587. Outside these hours, call the Fremantle Hospital (FSHFG group) on call pharmacist on +61 (0)4 1033 2943.

6.17 Withdrawal from the trial (or change of permissions)

Participants can choose to withdraw for any reason at any time during their involvement in the trial. Participants can withdraw from: (a) taking study medication, although participants who have taken at least one study medication capsule will be informed that they should take the treatment regimen for at least 16-weeks (i.e. until the primary outcome timepoint) before deciding if the treatment works or not; and (b) providing data to the trial, at any time for any reason without affecting their usual care. The PI can also decide to withdraw participants based on clinical opinion at any time during the trial; a likely scenario might be withdrawing them from taking the study medication. This may be due to safety concerns, severe adverse effects, development of mania or psychosis, or loss of contact such that sending further medication is not deemed safe. Trial specific instructions will be provided to PIs for withdrawal criteria and procedure.

In all cases, efforts will made to report the reason(s) for withdrawal/change of permissions in a STRATA Change of permissions/Withdrawal form. If a participant who has taken at least one study medication capsule wishes to withdraw from taking study medication (receiving the allocated trial treatment), efforts will be made to continue to obtain follow up data, with the permission of the patient or family as appropriate (including access to medical notes / databases). The study would also retain, confidentially, any data collected up to the point of withdrawal for analysis, as advised in the PIL. We would also like to have the option to collect data from their electronic records, in the future, unless they request otherwise.

In the event of any form of withdrawal (or relevant change of permission), participants who have taken at least one study medication capsule will be provided information about the down titration of the study medication and how to safely dispose of study medication. See Sections 9.12 and 9.13 for further details about titration for side-effects, and discontinuation of trial medication, respectively.

6.18 End of trial

Participant: The participant ends their involvement with the trial when their last assessment is completed (or they have withdrawn from the study) and have completed their down titration period of the study medication.

Trial: The end of trial for STRATA will be when the last patient has completed their 52-week (12-month) follow-up, which includes completion of the 52-week questionnaire, all data queries have been resolved and the database has been locked, with subsequent data analysis completed.

6.18.1 Trial stopping rules

The trial may be prematurely discontinued by the Sponsor, Chief Investigator (CI), Regulatory Authority or Funder based on new safety information or for other reasons given by the DMC / TSC regulatory authority or ethics committee concerned.

The trial may also be prematurely discontinued due to lack of recruitment or upon advice from the TSC, who will advise on whether to continue or discontinue the trial and make a recommendation to the Funder. If the trial is prematurely discontinued, no new participants will be recruited, and a decision on data collection on active participants will be made in discussion with the TSC/DMC and Sponsor.

See Section 10.11 for details about urgent safety measures (USMs).

6.19 Carer involvement

Carers will be recruited in parallel to explore carer burden in a nested sub-study; full details are provided in Section 8 and Table 3 depicts timings of data collection. It is up to the STRATA participant to define who their carer is; where feasible, it would be one carer who knows them well and is likely to continue caring for them over the 52-week trial period. The carer will be approached only with the agreement of the participant and consented separately. The participant is still able to participate in the trial if carers do not wish to participate, or if there is no carer. Similarly, the carer can continue involvement if the participant withdraws from the main trial.

7 QUINTET RECRUITMENT INTERVENTION (QRI) AND INTEGRATED QUALITATIVE RESEARCH

7.1 QuinteT Recruitment Intervention (QRI) *[UK only]*

Large placebo-controlled medication trials in adults with autism have not been conducted to date. For example, the largest placebo-controlled trial to assess the effectiveness of an SSRI for adults with autism had just 37 participants. Therefore, very little is known in relation to recruiting to and carrying out substantive trials in the adult autism population. We think recruiting participants could be challenging for several reasons: (1) conducting an RCT to assess effectiveness of treatments relies on uncertainty, i.e. randomisation and concealed treatment allocation, which could be difficult for people with autism (55); and (2) published evidence from RCTs in adults with developmental disabilities suggest that clinicians can be reluctant to recruit to such studies (56).

We have carefully planned for this possibility and will implement a QuinteT Recruitment Intervention (QRI) to understand and optimise the recruitment to trial process (57), and integrate qualitative research to explore participants' decisions, acceptability and experiences of trial participation and study medication.

The QRI will be implemented in STRATA's UK centres/sites to optimise recruitment and informed consent during the internal pilot (months 1-9 of recruitment) and the first nine months of main trial phase (months 10-18 of recruitment). Lessons learnt will be shared with the Australian centre. Originally developed in the NIHR HTA programme-funded ProtecT (Prostate testing for cancer and Treatment) study, the QRI has been subsequently refined and applied to over 40 RCTs leading to insights about recruitment issues and the development of targeted strategies that can improve recruitment rates (58-60). It will be implemented in two phases:

7.1.1 QRI - Phase 1: Understanding recruitment

QRI - Phase 1 will investigate the recruitment process and how it operates within centres/sites, building up a comprehensive understanding of recruitment challenges that arise during the internal pilot and beyond. A multi-faceted, flexible approach will be adopted, using one or more of the following methods:

Mapping patient eligibility and recruitment pathways: Detailed eligibility and recruitment pathways will be compiled for clinical sites, noting the point at which potential trial participants receive information about the trial, which members of the clinical/research team they meet, and the timing and frequency of appointments. Recruitment pathways will be compared with details specified in the trial protocol and pathways from other centres/sites to identify practices that are potentially more or less efficient. The QRI researcher will work closely with the trials unit managing the study (Bristol Trials Centre (BTC)) to compose detailed logs of potential RCT participants as they proceed through screening and eligibility phases, documenting the numbers of screened, eligible, approached and randomised patients (SEAR framework (47)) to help identify points at which they do not continue with recruitment to the RCT. Reasons for ineligibility and eligible patients not being approached or declining randomisation will be noted. Logs will be assembled using simple flow charts and counts to display numbers and percentages of patients at each stage of the eligibility and recruitment processes. These figures will be compared across centres and considered in relation to any estimates that may be specified in the grant application or study protocol.

Recording and observation of recruitment appointments: Any consultation in which the STRATA study is discussed with potential participants up until their study participation decision (this includes the

baseline (first study) appointment and consultations prior to this) will, with permission, be audio-recorded on an encrypted audio-recorder (or recorded via an alternative secure device/mechanism, including Sponsor/NHS-approved video-conferencing tools). The recordings will be used to explore study and treatment information provision, recruitment techniques, patient concerns, and randomisation decisions to identify recruitment difficulties and improve information provision.

In-depth interviews: To be undertaken with: (a) Members of the TMG including the CI and those closely involved in the design, management, leadership and co-ordination of the trial; (b) Health professionals and researchers who are involved in trial recruitment (trial recruiters); and (c) Patients who have been approached to take part in the trial.

Interviews with TMG members and trial recruiters will investigate their perspectives on the RCT and experiences of recruitment (where relevant). Key topics to explore will include views about the trial design and protocol; understandings of the evidence on which the trial is based; perceptions of uncertainty/equipoise in relation to the use of SSRIs in adults with autism; views about how the protocol is delivered in clinical sites; methods for identifying eligible patients; views on eligibility; and examples of recruitment success and difficulties. It is anticipated that at least one key person from each participating UK centre will be interviewed. Interviews will be undertaken over the telephone/ Sponsor-approved video-conferencing tools, or at a suitably private and quiet setting face-to-face.

Interviews with participants who have been approached about the trial will explore views on the presentation of study information, understanding and acceptance of trial processes (including, for example, randomisation, placebo, blinding), and reasons underlying decisions to accept or decline trial participation. Where possible, interview and audio-recorded data for the same patient will be compared to identify differences in actual and recalled information. Participants will be purposefully selected, to build a maximum variation sample on the basis of age, gender, study site, and final decision about trial participation (i.e. accept/decline). Numbers will be dependent on whether sufficient understanding has been gained on issues raised (data saturation). Interviews will be undertaken at the participant’s preferred location (e.g. home, university) and through their preferred method (e.g. face-to-face, telephone, Sponsor/NHS-approved video-conferencing tools), assuming they are in a suitably private and quiet setting. Any incurred travel costs will be reimbursed.

All interviews will be audio-recorded on an encrypted (secure) device (or recorded via an alternative secure device/mechanism, including Sponsor/NHS-approved video-conferencing tools) and a topic guide will be used to ensure the key areas stated above are covered but with flexibility to let the participants raise issues of importance to them.

Attendance at TMG and investigator meetings: The QRI researcher will attend TMG and investigator meetings to gain an overview of trial conduct and overarching challenges (e.g. logistical issues etc.). Attendance at these meetings can elucidate new lines of inquiry and add new dimensions to challenges that have emerged through other data collection methods.

Review of study documentation: The QRI team will work closely with the BTC and Patient and Public Involvement (PPI) group to ensure that patient-facing study documents are unbiased and clear. As the study progresses, the PIL and consent form(s) will be compared with interviews and recorded appointments to identify any disparities or improvements that could be made.

7.1.2 QRI - Phase 2: Development and implementation of recruitment strategies

Findings from Phase 1 of the QRI will be presented to the CI/TMG, identifying factors that appear to be hindering recruitment. The CI/TMG in discussion with the QRI team will formulate a ‘plan of action’

grounded in these findings to improve recruitment and information provision, with its format dependent on the nature of the recruitment barriers identified. Supportive and sensitive feedback and training is likely to be a core component of the plan of action. Centre-specific feedback may cover institutional barriers such as patient pathways, while multi-site group feedback sessions may address widespread challenges that would benefit from discussion, such as issues around equipoise or patient concerns. All group feedback sessions will be aided by anonymised data extracts from interviews and audio-recorded appointments. Individual confidential feedback may also be offered – particularly where recruiters experience specific difficulties or where there is a need to discuss potentially sensitive issues. Other interventions are likely to include ‘tips’ documents for recruiters, providing suggestions on how to explain the trial design and processes to aid participant understanding, and investigator meetings and/or site visits to discuss technical or clinical challenges (e.g. discomfort surrounding eligibility criteria).

QRI - Phases 1 and 2 will be undertaken in an iterative and cyclical manner, continuing throughout the first 18-months of recruitment with close monitoring of changes in screening log data and recruiter practice. The QRI research team will work closely with the CI/TMG, specialists in autism and the advisory group members to deliver recruitment strategies that are sensitive and appropriate to the target study population to optimise recruitment and informed consent.

7.2 Integrated qualitative research – experiences and acceptability of study and treatment *[UK only]*

Alongside the QRI, semi-structured qualitative interviews will be conducted with trial participants to explore views, expectations, experiences and acceptability of the study processes and treatment, along with issues around adherence. Up to 30 patient interviews will be undertaken for both this and the QRI component of the trial, with timings depending on the key issues to explore. The final sample size will be driven by data saturation. Some participants may be interviewed at more than one timepoint, for example to explore trial information provision and participation decisions in the early stages as part of the QRI, and later to explore acceptability of treatment or decision to withdraw from the trial or treatment. Participants will be purposefully selected to ensure maximum variation in terms of age, gender, recruiting centre/site and engagement with the medication (e.g. withdrawal).

Topic guides will be used to ensure similar topics are covered in each interview but applied in a flexible manner enabling issues of importance to the participants to emerge. The guide for this aspect of the trial will focus on experiences including autism and anxiety pre- and post-study treatment, previous experiences of anxiety treatment, treatment and recovery expectations, acceptability and experiences of the treatment received, adherence to it, and reasons for withdrawing from the medication or study if applicable. Location and method of interviewing will be led by the participant’s preference where possible. For all patient interviews, the qualitative researcher will adhere to the latest University of Bristol lone working guidance.

7.2.1 Study staff/healthcare professional consent

Recruiting staff and TMG member consent will be obtained through a ‘master’ consent form that covers all aspects of the QRI; this will be a one-off process to cover consent for interviews and recordings of appointments. Members of the QRI/Integrated Qualitative Research team will be responsible for obtaining this consent and maintaining suitable records.

To enable remote and/or face-to-face methods of contact, where feasible, informed consent will be captured via a MHRA compliant eConsent (online) form and process. An approved paper (wet ink)

equivalent will be available where eConsent is not feasible. Further guidance will be provided in study-specific training materials.

Three copies of the completed consent form are required;

1) a copy must be filed in the ISF together with a copy of the relevant information leaflet in recruitment order*;

2) a copy should be given to the participating member of staff*; and

3) a copy should be given to the STRATA central research team/ QRI/Integrated Qualitative Research team.

**If a paper (wet ink) form is completed, the 'original' form should be filed in the ISF. If an eConsent form is completed, a completed copy is emailed to the participant once processed, and additional copies can be obtained via the eConsent (database) system.*

7.2.2 Patient consent

Recordings of recruitment discussions: All consultations in which the STRATA study is discussed will be audio-recorded (or recorded via an alternative secure device/mechanism, including Sponsor/NHS-approved video-conferencing tools) with consent, up until the point at which the patient decides to accept or decline study participation. Information about the QRI is provided to patients in the study PIL. If patients have not yet been given a PIL but are having an appointment in which the trial will be introduced/discussed, then verbal consent for recording will be taken at the start and written consent sought once they have had time to read the PIL (i.e. by eConsent or post). It will be explained to patients that recordings are undertaken to explore how treatment and study information is conveyed to potential trial participants, and that if they wish the recording can be erased once they have had sufficient time to read and discuss the PIL.

At the start of the baseline (first study) appointment, recruiters will check if the patient has read the study PIL and ask if they have any questions about the recording process. Patients who agree will sign a STRATA Recording Consent Form that seeks permission to record all discussions about the trial in the lead up to the patient making their decision about participation. Some patients may have already given verbal consent for this in earlier discussions. If the patient declines a baseline appointment but a recording earlier in the study pathway has been made with their verbal consent, then written consent should still be sought. If there is no face-to-face appointment, then the recording consent can be completed online (i.e. via eConsent) or a form can be posted to patients with a pre-paid envelope for return. Recordings will not be transferred off site until written consent (paper or eConsent version) has been obtained. Recordings will be destroyed if no written consent has been obtained.

Interviews: Information about patient interviews is in the PIL. On the Recording Consent Form, patients will be asked if their contact details can be passed on to a study researcher to contact them about an interview to explore their views on the study. They may agree to this even if they decline the recording of consultation and/or they subsequently decline trial participation. Patients who consent to this may then be approached by the qualitative researcher who will explain more about the interview, answer any questions, and, if they agree, arrange a convenient time and preferred method (e.g. face-to-face/phone/video/email) to conduct the interview. Patients will be able to complete consent online (eConsent) or be posted an interview consent form and pre-paid response envelope, where necessary. If the form has not been returned at the time of interview, then verbal consent will be recorded and written consent chased.

Centre/site researchers, or members of the QRI/Integrated Qualitative Research team where relevant, will be responsible for obtaining appropriate consent and maintaining suitable records, including the secure destruction of recordings where written consent has not been obtained. Three copies of the completed written consent form are required, akin to those noted in Section 7.2.1, above.

7.3 Analysis of QRI and integrated qualitative research data

Interviews and recruitment consultation data will be transcribed verbatim in full or part. Data will be managed using qualitative data analysis software (such as NVivo [QSR International Pty Ltd.]). Interviews and recruitment consultations, along with screening logs and study documentation, will be subject to simple counts, content, thematic and targeted conversation analyses. Preliminary analysis will be used to inform strategies for Phase 2 of the QRI and further data collection. The QRI/qualitative team will independently analyse a proportion of transcripts to assess the dependability of coding and meet regularly to review coding and descriptive findings, agree further sampling and training strategies, and discuss theoretical development – all in close collaboration with the CI and the wider study team.

Results from the QRI and integrated qualitative research will help to optimise recruitment and informed consent in ‘real time’ as recruitment is progressing, as well as understanding how adults with autism accept and experience an RCT and treatment (active or inactive) for anxiety.

7.4 Data management, protection and patient confidentiality in relation to the QRI and qualitative research data [applicable to UK only]

Recruiting staff at centres/sites will be requested to set up the audio-recorder (or alternative Sponsor/NHS-approved video-conferencing tools) during recruitment discussions with potential participants. Recruiting staff will be requested to store the audio-recorder securely in a locked drawer/cabinet when not in use and return study-owned equipment to the central research team securely at the end of the study.

Recordings of appointments in which the trial is discussed will be held on an encrypted digital recorder (or alternative secure device/mechanism, including Sponsor/NHS-approved video-conferencing tools) and regularly transferred to the University of Bristol through approved secure data transfer facilities and/or encrypted memory card/flash drives that adhere to NHS Trust policies. If a video-conference platform is used to record discussions, only the audio file will be downloaded and retained for analysis. Interview data captured on audio-recorder will be transferred to a secure server hosted by the University of Bristol as soon as possible after each interview.

Recordings will be transcribed by University of Bristol employees or University approved transcription services. The transfer of recordings and transcripts will adhere to the secure transfer of recordings/transcripts procedure specified by the University. Transcripts will be labelled with a study I.D number, edited to ensure anonymity of respondents and stored securely adhering to the University’s data storage policies. Anonymised quotations and parts of voice modified recordings may be used for training, teaching, research and publication purposes for this and future studies. Anonymised transcripts may be made available by controlled access to other researchers who secure the necessary approvals for purposes not related to this study, subject to individual written informed consent from participants.

7.5 Safeguarding patients during QRI and qualitative research

We will ensure that participants are not subjected to undue distress during the QRI and qualitative component of the trial. To mitigate this, and the possibility that participants may disclose information to provoke concern about risk, the interviewer will be an experienced qualitative researcher who will adhere to the following:

Participants will be informed that the interview is strictly confidential, but should they disclose information to suggest that they or others are at significant risk of harm, the interviewer will discuss this with a clinical advisor and may need to disclose these details to the designated safeguarding authority. The interview will only continue if participants are happy to proceed and engage with the interview topic. If the researcher feels a participant becoming distressed, they will ask the participant if they wish to have a break or discontinue the interview and will offer support. A study-specific working guide will be put in place for such circumstances, for example a protocol for interview if there are indicators of concern. Participants will also be offered a leaflet with the contact details of support networks.

8 NESTED STUDY - EXPLORATION OF CARER BURDEN *[UK AND AUSTRALIA]*

As requested in the original NIHR HTA (UK) and NHMRC (Australia) funding application call, we aim to explore how the treatment of anxiety for adults with a diagnosis of Autism influences the burden to their carer(s).

8.1 Objective and outcome measures

To determine the effect of up to 52-weeks of treatment with sertraline versus placebo on carer burden.

Carers will be asked to complete a STRATA Carer Burden Questionnaire at Baseline, 16- and 52-weeks post-randomisation of the STRATA (main trial) participant. Carer burden will be measured using the Caregiver Burden Scale (42). Other outcomes/measures include the EQ-5D-5L (41) and Carer Experience Scale (CES) (43-45) and brief questions about the anxiety and autism of the main trial participant (i.e. the person for whom they are a carer).

Information about the carer will also be collected at Baseline, for example (as a minimum): name; contact details and preferences; basic demographic information (e.g. year of birth; gender; ethnicity; employment status; highest level of qualification; marital status); their caring role; whether they are a paid/unpaid carer; full-time/part-time carer; and relationship to the STRATA participant. Some of this information will also be collected at 16- and 52-weeks to identify any relevant changes.

8.2 Recruitment and consent

Figure 5 illustrates the flow of this sub-study, and what is involved.

For the purpose of this carer burden sub-study, it is up to the STRATA (main trial) participant to define who their carer is; where feasible, it would be one carer who knows them well and is likely to continue caring for them over the 52-week trial period.

As noted in Section 6.7.3, if a participant confirms that they have a carer, the researcher will provide the participant with a STRATA Carer Study Information Pack. The participant will be asked to forward this pack to their carer at the earliest opportunity, on behalf of the STRATA research team*.

> **A researcher will check with the participant, during an early safety check follow up call, if they have provided their carer with the pack; if not, they will be asked again to do so. To reduce burden to the participant, however, no further reminder will be given hereafter.*

The STRATA Carer Study Information Pack will consist of an approved Study Invitation Letter, PIL, Consent Form, Questionnaire, and pre-paid (freepost) envelope. Contact details of the local research team will be provided in the event of any queries. Where feasible, an online equivalent of the Carer Study Information Pack (or at least the provision of information and ‘what to do next’ if you are interested in taking part) will be available on the study website.

Alongside explicit consent to participate in the study, consent will be sought for future contact about other research, and sharing of their anonymised data for other ethically approved studies (as a minimum).

We envisage the completion and return of consent and Baseline questionnaire (at least) to be via posted paper copies given the initial method of information provision, however alternative remote methods of contact will be available, where feasible. For example, a MHRA (UK) and NHMRC and TGA (Australia) compliant eConsent (online) form, and data collection via secure online links (surveys) and/or video-conferencing/ telephone calls with the researcher. Further guidance for the researchers will be provided in study-specific training materials.

Three copies of the completed consent form are required;

1) a copy must be filed in the ISF together with a copy of the PIL in recruitment order*;

2) a copy should be given to the carer* (electronically, or via post with the next questionnaire); and

3) a copy should be given to the STRATA central research team.

**If a paper (wet ink) form is completed, the 'original' form should be filed in the ISF. If an eConsent form is completed, a completed copy is emailed to the participant once processed, and additional copies can be obtained via the eConsent (database) system.*

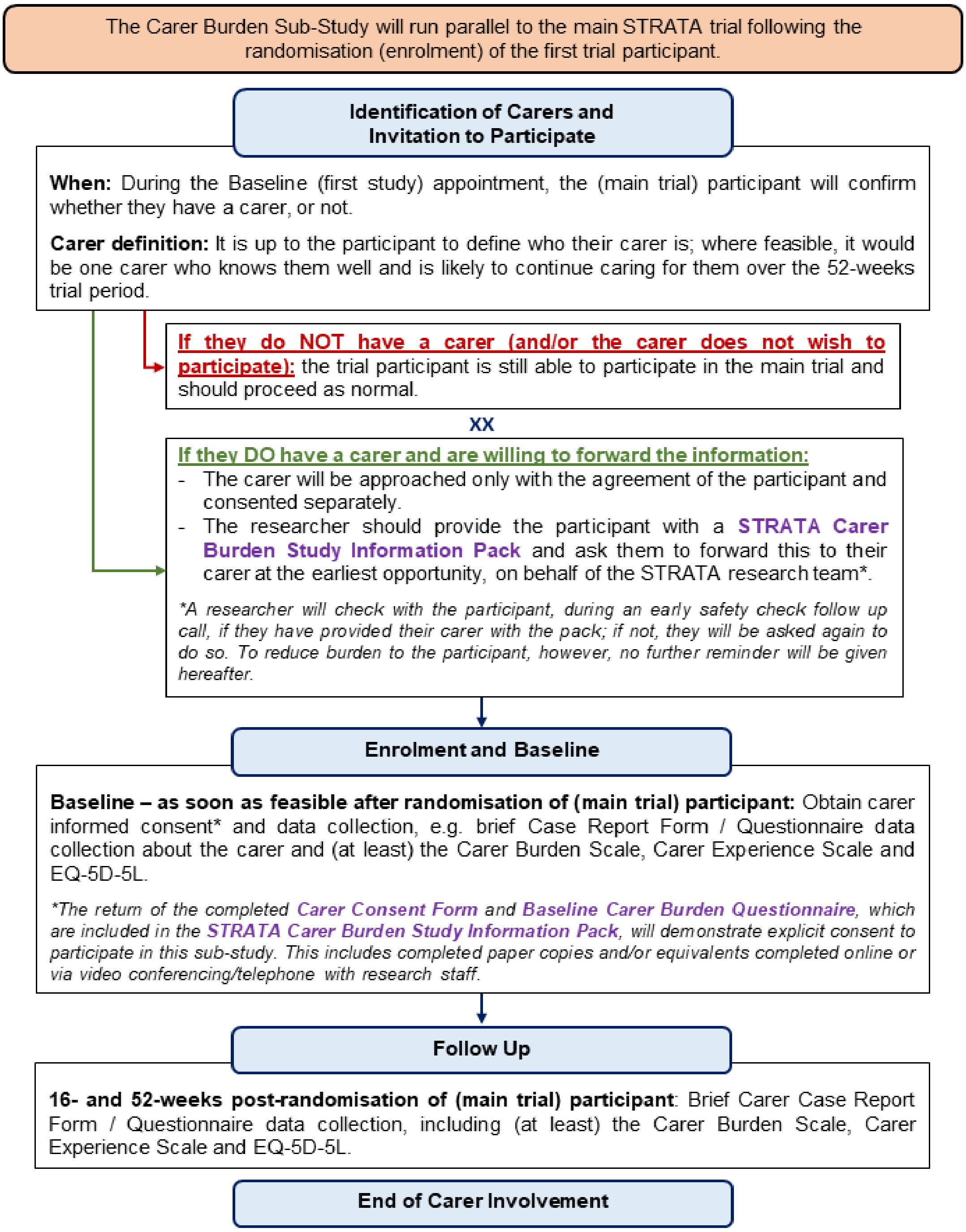

Figure 5 Carer Burden Sub-Study flowchart

<u>All carers who agree to take part</u> will be logged with the relevant local research team (UK / Australian) and allocated a study specific (Carer) I.D number. The relevant research team will send a

subsequent Carer Burden Questionnaire at 16- and 52-weeks post-randomisation (of the STRATA participant); see Table 3. Questionnaires will be available for carers to complete via various alternative methods (e.g. online, telephone/video conferencing, or post) to minimise loss to follow up. If the local research team does not receive a response from a carer within a reasonable time of sending the Carer Burden Questionnaire at 16- and 52-weeks (e.g. ~3 weeks), then they will try calling them, and/or resend another online link/pack with a reminder letter (or email equivalent). For each questionnaire, the research team will make up to three contact attempts (initial sending, plus two reminders). If no response is received after the third attempt, the relevant questionnaire will be marked as missing. We will, however, continue to send the next questionnaire as planned, unless the participant requests/ confirms that they no longer want to complete them; a similar model has been successfully used in multiple studies conducted by the BTC. Return of a completed 52-week questionnaire marks the end of the carer's direct involvement.

8.3 Information about the study

To thank them for their time and keep them informed, carers will be sent relevant newsletters telling them about the study, including progress and results once available. Further details about dissemination are outlined in Section 16.

8.4 Withdrawal criteria

Carers will remain in this sub-study unless they choose to withdraw, or if they are unable to continue for a clinical reason, as notified by the participant. The carer does *not* need to withdraw from this sub-study if the trial participant (i.e. the person for whom they are a carer) withdraws from the main trial. If a carer withdraws consent, data collected up to the point of withdrawal will be retained (confidentially) for analysis, as stated in the PIL. A study Carer Change of Permissions/ Withdrawal Form will be completed in all cases, and databases updated accordingly. Study specific procedures for a carer's change of permissions, or withdrawal, will be outlined in the relevant trial working guidelines, which appropriate members of the research team will be made aware of. The Carer Burden Sub-Study PIL will include information for carers. Carer post-study care does not apply for this element of the STRATA trial.

8.5 Adverse Events

Adverse events (AEs) are typically defined as *"any unfavourable and unintended signs, including abnormal laboratory results, symptoms or a disease associated with treatment/ procedures required by the protocol".* For the purpose of this (low risk) carer sub-study, we do not expect, nor will actively seek, any AEs related to research procedures, such as completing a questionnaire. If, however, we become aware of relevant information about a carer regarding an AE related to taking part the carer burden sub-study, we will ensure that it is recorded. In those situations, we would liaise with the Sponsor within a timely manner, noting their relevant safety reporting standard operating procedures (SOPs) and supporting documents.

8.6 Analysis of carer burden data

See Section 11 (Statistics and Health Economics Analysis) for details, including sample size.

8.7 Data Management

See Section 12 (Data Management).

9 INTERVENTION / INVESTIGATIONAL MEDICINAL PRODUCT (IMP)

9.1 General information

The active investigational medicinal products are over-encapsulated 25mg or 50mg Sertraline tablets with a back fill of microcrystalline cellulose powder. The placebo product is a matched capsule filled with microcrystalline cellulose powder. Sertraline is licensed for the treatment of depression as well as numerous anxiety disorders including panic disorder, with or without agoraphobia; OCD in adults and paediatric patients aged 6-17-years; social anxiety disorder and PTSD.

9.2 Assessment and management of risk

This trial is categorised as 'Type A' according to the Medicines and Healthcare products Regulatory Agency (MHRA; testing authorised medicinal products according to treatment regimens within the marketing authorisation) (UK), and the National Health and Medical Research Council (NHMRC; risk-based management and monitoring of clinical trials involving therapeutic goods) (Australia). Sertraline is marketed and used for the treatment of depression and anxiety. Its safety profile is well characterised in the general population. It is also commonly used for the treatment of depression and anxiety in the autistic population although no large RCTs of its effectiveness or characterisation of safety profile in this population have been carried out. Using sertraline for anxiety in people with a diagnosis of autism therefore means that we are focussing on a specific patient group in what is a well-established treatment regimen for depressive and anxiety symptoms. Up-titration to 100mg is routine in clinical practice and higher doses of up to 200mg are licenced and sometimes recommended for treatment of anxiety disorders.

It is not anticipated that application of placebo represents any risk above that of standard care.

9.3 Manufacture of IMP

Over-encapsulation of the active tablets will be done by Eramol Ltd (formerly RenaClinical), a UK Manufacturer and Importation Authorisation (MIA) IMP licence holder, as detailed below. The placebo product will be manufactured by Eramol.

9.4 Packaging, labelling and shipping of IMP

The IMP will be packaged and labelled by: Eramol , Unit 11, Gatwick Metro Centre, Horley, RH6 9GA (UK), or their other manufacturing site, Unit 9 North Downs Business Park, Limepit Lane, Sevenoaks, TN13 2TL (UK). The core label texts for all packaging will comply with the requirements of Annex 13 of the Rules Governing Medicinal Products in the European Union and the national laws in force in the UK. Eramol will perform Qualified Person (QP) release prior to shipping to the central hospital pharmacies in the UK and Australia. Shipping will be undertaken by an Eramol approved sub-contractor.

9.5 Storage of IMP at central pharmacy

The central hospital pharmacies (UK and Australia) will be required to nominate a research pharmacist and deputy who will be responsible for the IMP. Storage requirements will be detailed in a trial specific working instruction.

9.6 Prescribing procedure

The trial medication will be prescribed by the local "prescriber" (i.e. local PI, or their delegated representative). The local "prescriber" (local PI), or their representative, should complete the trial-

specific prescription form and forward to the relevant central trial pharmacy (UK or Australia) for onward dispensing to participants; see section 9.7, below. Further details about the prescribing procedure(s) will be provided in the study-specific working instructions.

9.7 Dispensing of IMP

The central UK (UHBW) and Australian (Fiona Stanley Hospital Pharmacy) pharmacies, as applicable, will be responsible for dispensing the IMP and will maintain their respective IMP dispensing logs. The trial pharmacist who fulfils the trial prescription and sends the medication to the participant will be unblinded.

UK: the trial pharmacy will post the medication directly to the participant's specified address via specialist registered postal service (default option). Alternatively, if participants prefer, delivery to their local pharmacy or GP (doctor) surgery will be explored.

Australia: As the study pharmacies will be directly informed of the randomisation outcome, patients in Australia will have the option of collecting the medication directly from the local hospital pharmacy (default option). If preferred, options for participants to collect from their GP or have it delivered to their specified address will be explored.

Participant's GPs (or equivalent healthcare professional in Australia) will be sent detailed information about sertraline and potential adverse effects via the notification letter that their patient is enrolled in the STRATA trial; the information about sertraline and potential adverse effects will also be available via the trial website. Participants will also receive similar information. We will use autism-accessible instructions to support the medication regime co-produced with help of our advisory group.

9.8 Return and destruction of IMP

Participants will be asked to safely dispose of unused trial medication by returning them to a local pharmacy for destruction. Individuals who are sent study medication but change their mind before starting the medication will be asked to return it for confirmation of untampering (e.g. pill count or untampered packaging, as applicable). In both cases, relevant instructions will be provided.

Any unused IMP that is returned directly to the trial pharmacies will be destroyed in line with the central pharmacy trial specific instructions on the disposal of IMP.

9.9 Dosing schedule / Administration and titration

Appendix 1 provides tabulated details of the STRATA dosing schedule and titration, as well as stopping instructions. To summarise, all patients will receive a daily dose of 25mg sertraline or placebo for 2 weeks followed by 2x25mg for 4 weeks. Following this initiation period, the medication will be dispensed in 50mg capsules and depending upon tolerability, the dose will be **flexibly** increased by 50mg every 4-weeks to reach the optimal dose. The dose will only be increased if the participant is tolerating it and agrees to try an increased dose, and the prescribing clinician is satisfied that it is appropriate to do so based on the participant's responses to the safety check questionnaire and discussion with the study RA, as described above. The dose may go up to a maximum of 200mg by week 14, although it is anticipated that for many patients the optimal dose may be lower than this amount (e.g. 50mg, 100mg or 150mg) and reached before this time. Participants will take this optimal dose for up to 52-weeks post-randomisation. The placebo regimen will be identical.

During the period of dose titration, participants will have four contacts with a member of their local research team (at 1 to 2-, 4-, 8- and 12-weeks post-randomisation) for brief data collection on adverse effects, mood, anxiety and suicidal ideation. A similar 'brief safety check' will also take place at 36-

weeks post randomisation. Participants will also be able to contact the study team at any point should they experience any adverse event related to the trial medication. Please refer to Section 9, Pharmacovigilance, for trial specific instructions regarding safety reporting. After 52-weeks post-randomisation (or if they discontinue medication sooner), participants will be asked to complete a downward titration of the study medication for a period of up to four weeks (which involves reduction of medication by 50mg per week) before being returned to standard care.

9.10 Assessment of adherence to treatment

Medication adherence (compliance) will be assessed using methods/measures outlined in Section 2, and presented in Table 1 and Table 3. We aim to minimise burden to participants, while meeting the needs of the MHRA, Sponsor and study. In brief, this will include questions adapted from the GENPOD (38) and PANDA (39) trials about adherence to medications (at 1 to 2-, 4-, 8-, 12-, 16-, 24-, 36- and 52-weeks post-randomisation).

STRATA is a pragmatic trial. While information about taking the study medication, including possible ways to aid adherence will be included in the STRATA Medication Instructions, we do not propose any additional measures to improve poor treatment adherence amongst participants as that may not reflect real life practice and act as an additional intervention.

In the case of persistent non-adherence to treatment, a pragmatic clinical decision will be made. For example, if a participant is on a higher dose of study medication (e.g. 150mg) and reports persistent non-adherence, it may be advisable to withdraw them from the trial treatment (but not from completing future study questionnaires). Such decisions will be made by the local PI in consultation with the CI on a case-by-case basis.

9.11 Common side-effects

A full list of possible side effects can be found in the supporting SmPC; the Reference Safety Information (RSI) for this trial is section 4.8 (undesirable effects) of the SmPC for Crescent Sertraline 50mg film-coated tablets, date of revision of the text 23rd September 2019.

The *most common* side effects of sertraline are:

- Nausea
- Dizziness
- Dry mouth
- Insomnia
- Drowsiness/fatigue
- Headache
- Diarrhoea
- Sexual dysfunction (in men and women)

Some people may also experience a worsening in anxiety and there may be worsening or emergence of suicidal ideation following initiation of sertraline treatment. These undesirable effects are dose dependent and are often transient in nature with continued treatment.

9.12 Titration for side-effects

In the event of a participant experiencing side effects the local research team will refer to the relevant trial specific instructions and consult with the PI where appropriate. This will determine whether down titration or ceasing the IMP is necessary. Examples for down titration or discontinuing the IMP are mentioned in Section 6.17. The same dosing schedule will be applied to participants in both groups with dummy dose titration being applied to the placebo group.

9.13 Discontinuation of trial medication

If the participant discontinues the medication because of unacceptable side effects or by choice, they will be advised to follow the downward dosing regime (see Appendix 1) before being returned to standard care treatment. They will be encouraged to remain enrolled in the trial (unless they explicitly withdraw; see section 6.17) and complete the further assessments as per protocol.

Once trial medication is discontinued, participants may not resume trial treatment but as this is a pragmatic trial, they may be prescribed any other medication including sertraline by their clinician.

9.14 Post-trial

Continuation of the treatment following the end of the downward titration (as described above) is the responsibility of the participant's usual clinician (secondary care specialist or GP), an arrangement which is described in the PIL. If a participant wants to continue taking medication post-trial, their usual clinician will be informed of their trial allocation.

9.15 Drug accountability

Table 4 Drug accountability for the STRATA trial

Activity	Responsibility
Supply of IMP (active and matched placebo)	Eramol
Provision and QP of IMP	Eramol
Package and labelling of IMP	Eramol
QP release IMP to trial pharmacy	Eramol
Distribution of IMP to central pharmacy	Eramol approved sub-contractor
Receive IMP and store appropriately	UHBW (UK) and Fiona Stanley Hospital Pharmacy (Australia)
Dispense IMP in line with prescription to participant	UHBW (UK) and Fiona Stanley Hospital Pharmacy (Australia)
Maintain dispensing log	UHBW (UK) and Fiona Stanley Hospital Pharmacy (Australia)
Report stock levels at site	UHBW (UK) and Fiona Stanley Hospital Pharmacy (Australia)
Return of unused trial medicines (where applicable)	UHBW (UK) and Fiona Stanley Hospital Pharmacy (Australia)
Destruction of unused trial medicines	UHBW (UK) and Fiona Stanley Hospital Pharmacy (Australia)
Unblinding	UHBW (UK) and Fiona Stanley Hospital Pharmacy (Australia)

9.16 Intervention and IMP COVID-19 considerations

Please see Appendix 2 for a summary of considerations and actions made by the central research team in relation to the trial and COVID-19. This includes examples regarding trial conduct and intervention/IMP considerations.

10 PHARMACOVIGILANCE

10.1 Operational definitions

Pharmacovigilance will be carried out in accordance with the requirements set out by Medicines for Human Use (Clinical Trials) Regulations 2004 and the Medicines for Human Use (Clinical Trials) (Amendment) (EU Exit) Regulations 2019 (UK), and Therapeutic Goods Administration, and the NHMRC Safety Monitoring and reporting in clinical trials involving therapeutic goods (Australia). This includes the terminology of adverse events and reactions and the assessment of seriousness, causality and expectedness of an event, in accordance with these regulations.

Term	Definition
**Adverse Event (AE)**	Any untoward medical occurrence in a participant to whom a medicinal product has been administered, including occurrences which are not necessarily caused by or related to that product.<br>*An adverse event can therefore be any unfavourable and unintended sign (including abnormal lab results), symptom or disease temporally associated with the use of the medicinal product/medical device/intervention, whether or not considered to be related to the medicinal product/medical device/intervention.*<br>Not all adverse events are adverse reactions but all adverse reactions are adverse events.
**Adverse Reaction (AR)**	An untoward and unintended response in a participant to an investigational medicinal product which is related to any dose administered to that participant.<br>The phrase "response to an investigational medicinal product" means that a causal relationship between a trial medication and an AE is at least a reasonable possibility, i.e. the relationship cannot be ruled out.<br>All cases judged by either the reporting medically qualified professional or the Sponsor as having a reasonable suspected causal relationship to the trial medication qualify as adverse reactions. It is important to note that this is entirely separate to the known side effects listed in the SmPC. It is specifically a temporal relationship between taking the drug, the half-life, and the time of the event or any valid alternative aetiology that would explain the event.
**Serious Adverse Event (SAE)**	A serious adverse event is any untoward medical occurrence that:<br>• results in death<br>• is life-threatening[A]<br>• requires inpatient hospitalisation or prolongation of existing hospitalisation[B]<br>• results in persistent or significant disability/incapacity<br>• consists of a congenital anomaly or birth defect<br>Other 'important medical events' may also be considered serious if they jeopardise the participant or require an intervention to prevent one of the above consequences.<br>*[A]The term "life-threatening" in the definition of "serious" refers to an event in which the participant was at risk of death at the time of the event; it does not refer to an event which hypothetically might have caused death if it were more severe.*<br>*[B]The definition of hospitalisation is an unplanned overnight stay. Note, however, that the patient must be formally admitted – waiting in outpatients or an Accident & Emergency Department (A&E) would not count as hospitalisation (even though this can sometimes be overnight). Prolongation of an existing hospitalisation qualifies as a SAE. Planned hospital stays would not be counted as SAEs, nor would stays in hospital for "social reasons" (e.g. respite care, the fact that there is no-one at home to care for the patient). Also, if patients had a day-case operation, this would not qualify as hospitalisation. However, if a planned operation was brought forward because of worsening symptoms, this would be considered as an SAE. Hospitalisations for the purpose of the intervention are an exception to SAE reporting unless complications occur.*
**Serious**	An adverse event that is both serious and, in the opinion of the reporting

**Adverse Reaction (SAR)**	Investigator, believed with reasonable probability to be due to one of the trial treatments, based on the information provided.
**Suspected Unexpected Serious Adverse Reaction (SUSAR)**	A serious adverse reaction, the nature and severity of which is not consistent with the information about the medicinal product in question set out in the reference safety information (RSI):<br>• in the case of a product with a marketing authorisation, this could be in the summary of product characteristics (SmPC) for that product, so long as it is being used within its licence. If it is being used off label an assessment of the SmPCs suitability will need to be undertaken.<br>• in the case of any other investigational medicinal product, in the investigator's brochure (IB) relating to the trial in question.<br>**The RSI for this trial is**: section 4.8 (undesirable effects) of the SmPC for Crescent Sertraline 50mg film-coated tablets, date of revision of the text 23rd September 2019.
**Suspected serious adverse reaction (SSAR)**	A suspected serious adverse reaction (SSAR), is any serious adverse reaction that is suspected (possibly or probably) to be related to the investigational medicinal product/medical device/intervention.

10.2 Classification of severity

**Mild event**:	An event that is easily tolerated by the participant, causing minimal discomfort and not interfering with everyday activities.
**Moderate event**	An event that is sufficiently discomforting to interfere with normal everyday activities.
**Severe event**:	An event that prevents normal everyday activities.

10.3 Classification of relatedness

**Not related**	Temporal relationship of the onset of the event, relative to administration of the intervention, is not reasonable or another cause can by itself explain the occurrence of the event.
**Unlikely to be related**	Temporal relationship of the onset of the event, relative to administration of the intervention, is unlikely and it is likely there is another cause which can by itself explain the occurrence of the event.
**Possibly related**	Temporal relationship of the onset of the event, relative to administration of the intervention, is reasonable but the event could have been due to another, equally likely cause.
**Probably related**	Temporal relationship of the onset of the event, relative to administration of the intervention, is reasonable and the event is more likely explained by the intervention than any other cause.
**Definitely related**	Temporal relationship of the onset of the event, relative to administration of the intervention, is reasonable and there is no other cause to explain the event, or a re-challenge (if feasible) is positive.

10.4 Classification of expectedness

**Expected**	Reaction previously identified and described in the Summary of medicinal Product Characteristics (SmPC).
**Unexpected**	Reaction not previously described in the Summary of medicinal Product Characteristics (SmPC).

NB: The RSI for this trial is: *section 4.8 (undesirable effects) of the SmPC for Crescent Sertraline 50mg film-coated tablets, date of revision of the text 23rd September 2019.*

10.5 Adverse events (AEs) classification flowchart

The PI of each participating site (or appropriate delegate, e.g. clinician, or CI if required) is responsible for assessing and categorising AEs. For each AE the seriousness, relatedness to IMP and expectedness will be determined (as per the definitions above) in order to appropriately classify and record and report (where applicable) the episode; see also Figure 6, below.

NB: *the expectedness of a SAR shall be determined according to the SmPC; hospitalisation for an elective procedure or for a pre-existing condition (prior to study entry) which has not worsened, does not constitute a SAE; and all SAEs will be followed until resolution.*

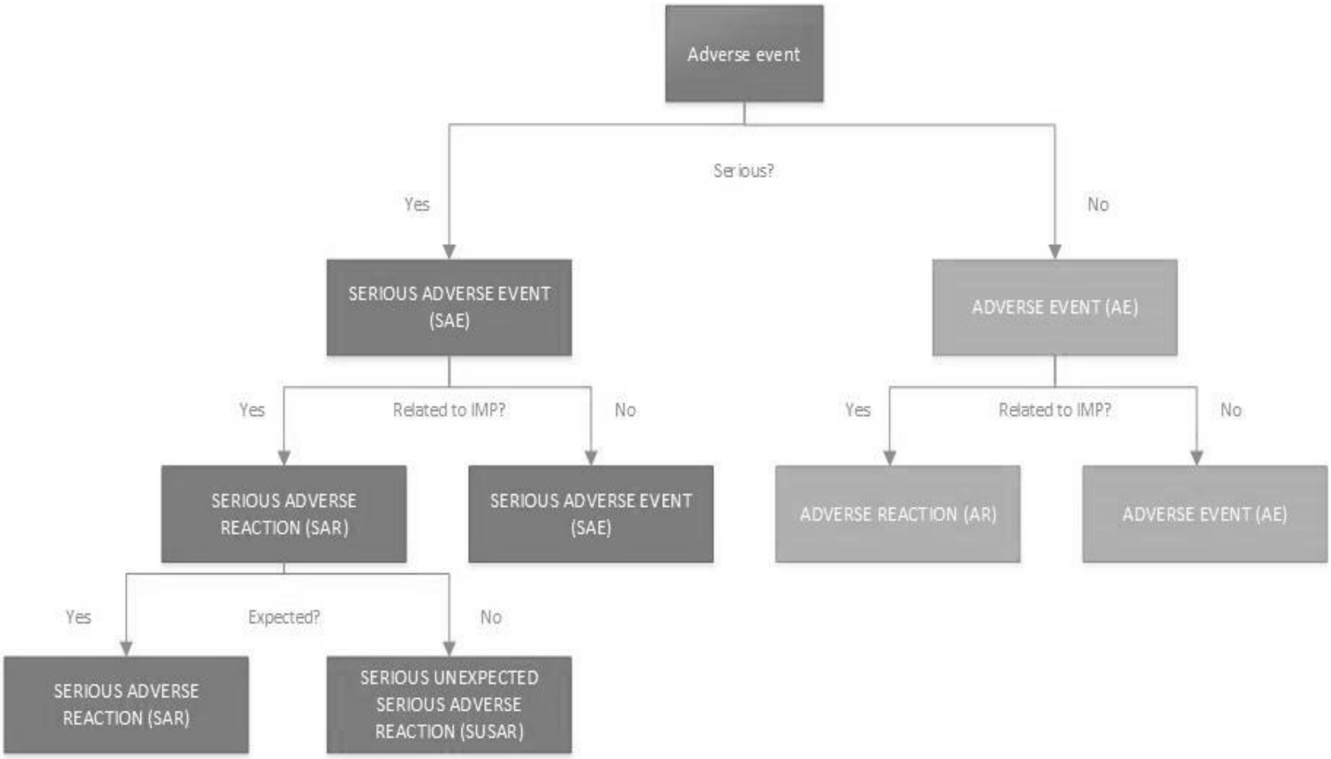

Figure 6 Classification of adverse events flowchart

10.6 Identification of AEs

AEs are expected to occur throughout the course of the trial. Local research teams are responsible for recording appropriate AEs for their participants during the trial. It is anticipated that most AEs will be detected via the brief (safety check) contacts and study questionnaires. The central and/or local research team will communicate with the local PI and sites if additional information is required, e.g. to ascertain the nature and severity of an AE. If a patient attends a routine (i.e. non-trial related appointment) and an AE is reported, the local research team will assess and log this according to trial recording and reporting procedures; see below.

10.7 Recording and reporting procedures

10.7.1 Non-serious AEs

Most non-serious AEs that are related to the IMP (adverse reaction; AR) will be expected reactions previously identified and described in the SmPC. New mental health diagnoses/symptoms that are unrelated to the IMP may also occur. These events will be collected by participant self-report using the Modified Toronto side effects scale and open-ended questions (including suicidality item) in the study questionnaires from the time a signed and dated informed consent form is obtained until completion of the last trial-related procedure for each participant. Events not captured by the questionnaires will be recorded in other study documentation (e.g. CRF). Where feasible, they should also be recorded in the participant's clinical notes by a suitable member of the local research team, however we acknowledge that this may not always be practical.

If an event is defined as 'serious' the local research team should proceed to follow recording and reporting procedures for SAEs, outlined below.

The recording framework for non-serious AEs is shown in Figure 7, below. A record of all recordable AEs (including SAEs) must be kept in the ISF.

The central research team (UK) will prepare regular summary reports of all recorded non-serious AEs for discussion at relevant oversight meetings.

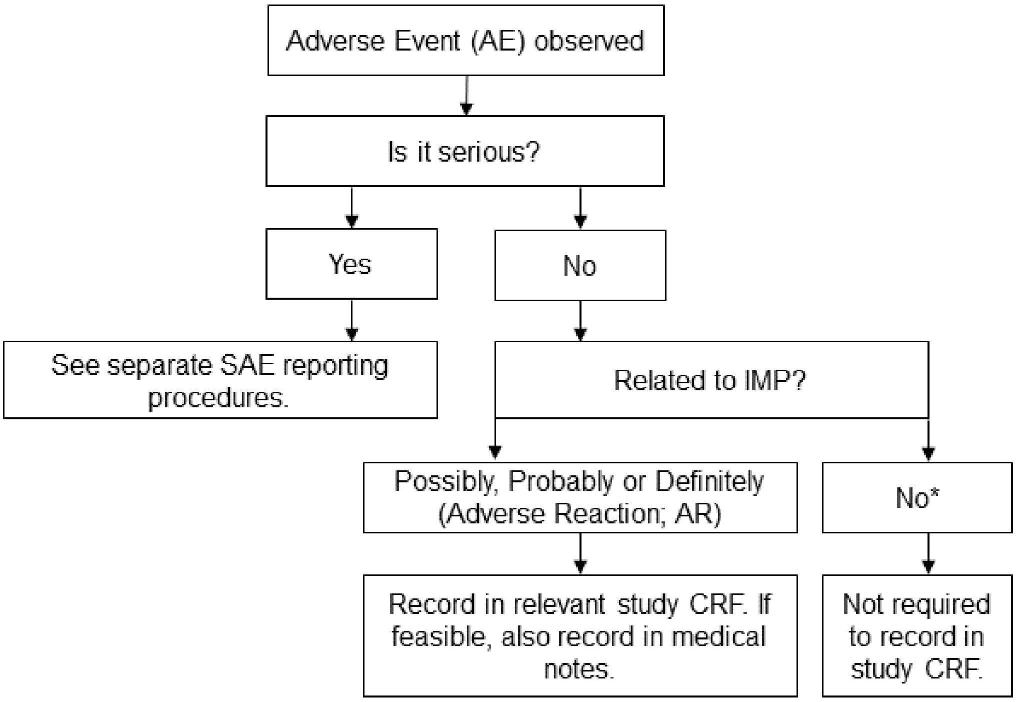

**with the exception of new mental health diagnoses/symptoms.*

Figure 7 Recording framework for non-serious Adverse Events (AEs).

10.7.2 Serious AEs (SAE/SAR/SUSARs)

Recording SAEs. Local research teams will record all SAEs (SAE/SAR/SUSARs) in the STRATA SAE Log, which should be retained in the ISF. Data capture will be via the study database, or paper records with subsequent data entry, where necessary. The central research team (UK) will review the SAE Log monthly for monitoring and reporting purposes and will prepare regular summary reports of all SAEs for discussion at relevant oversight meetings, including the DMC as per their written charter.

Reporting SAEs. The reporting framework for all SAEs is shown in Figure 8 , below.

Within 24-hours of becoming aware of a SAE, the local research team will notify (i) UHBW (on behalf of the Sponsor), (ii) the CI, and (iii) the central research team (UK). Where feasible, initial reporting will be in writing, but can be made orally. Oral reports will be followed up in writing within 24-hours of the initial report.

Written reports will use the trial SAE/SUSAR Initial Report Form; this includes SAEs spontaneously reported to the Investigator within 30-days after the participant has completed the intervention phase of the trial. UHBW, on behalf of the Sponsor, will evaluate any safety information that is spontaneously reported by the CI beyond the time frame specified in the protocol. Each SAE will be reported separately and not combined on one SAE form.

The local research team will provide information missing from the initial report within 5-working days of the initial report to the necessary bodies. Any change of condition or other follow up information relating to a previously reported SAE will be reported on a separate trial SAE/SUSAR Follow Up Report Form. All SAEs will be followed up until the event has resolved, or a final outcome has been reached.

Where possible, SAE recording and/or reporting will be via the study database. Where it is not possible to submit via the study database, a copy of the completed form(s) should be securely emailed, with high importance, to the following:

(i) UHBW (on behalf of the Sponsor): research@uhbristol.nhs.uk

(ii) The CI (Professor Dheeraj Rai): Dheeraj.Rai@nhs.net

(iii) the central research team (UK): awp.strata-rct-secure@nhs.net

The central research team will confirm email receipt with the local research team, as well as ensure receipt by UHBW for review. Where applicable, the local research team should update the study database as soon as feasible, thereafter.

> ***NB:*** *typical working hours of the central research team (UK): Monday to Friday, 09:00-17:00 (subject to change). In the event of University closure dates and/or being unavailable, an out of office automatic response will notify recipients of alternative contact details/arrangements.*

SUSARs will be further reported to the REC, DMC and MHRA within 7-days of the Sponsor being notified if fatal or life-threatening, or 15-days otherwise. **All SAEs** will be further reported to the DMC on a quarterly basis. UHBW will report any SUSARs to the MHRA on behalf of the Sponsor and the central research team (UK and Western Australia) will be responsible for all other reports to relevant regulatory authorities and/or trial oversight committees.

Australia: The Western Australian research team will follow the same procedures as described in this section, above. In addition, the Western Australian research team will report local SAEs (i.e. for Australian-recruited participants) to their Research Governance Office (RGO) within 72-hours, in line with the NHMRC requirements (Safety Monitoring and Reporting in Clinical Trials Involving Therapeutic Goods (Australia)).

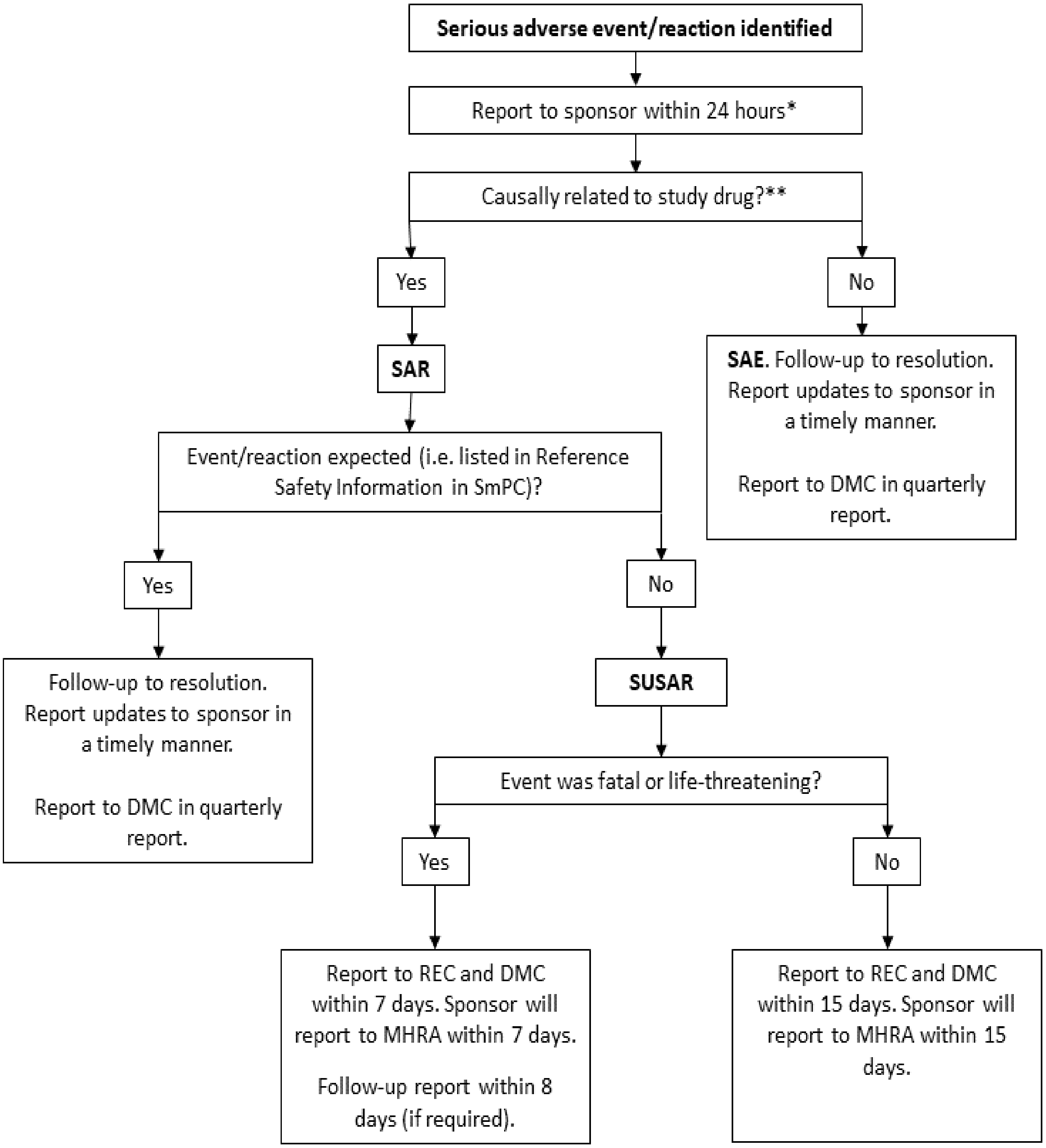

**refers to the initial notification to University Hospitals Bristol and Weston NHS Foundation Trust (UHBW) on behalf of the Sponsor.*

***causality should have been assessed prior to reporting to UHBW, however as part of their assessment on behalf of the Sponsor, UHBW will also review causality.*

Figure 8 Reporting framework for all Serious Adverse Events (SAEs).

10.8 Notification of deaths

All deaths occurring during the intervention phase of the trial or within 28-days after the last dose of trial medication will be reported immediately as soon as the central research team (UK) become aware.

10.9 Pregnancy

Should a study participant become pregnant whilst taking part in this trial, the pregnancy/ the participant will be followed up until term or termination and the Sponsor will be notified using the STRATA Pregnancy Notification Form. The BNF advice is that the benefits of the IMP should outweigh the risks if it were to be used in pregnancy (61). At baseline, we will exclude potential participants who are pregnant (as confirmed via GP safety check and patient self-reporting), are planning pregnancy during the trial period, or breastfeeding.

10.10 Overdose

There is a possibility of overdoses occurring in people with mental health problems. On the evidence available, sertraline has a wide margin of safety in overdose. Overdoses of sertraline alone of up to 13.5g have been reported. Deaths have been reported involving overdoses of sertraline, primarily in combination with other drugs and/or alcohol. Therefore, any over dosage should be medically treated aggressively. Information regarding an overdose is included within the supporting SmPC(s) and will be included in the participant's instructions about how to take the medication. If the research team are informed of a deliberate overdose, the participant will be advised to visit the emergency department (A&E) for medical care. There may also be incidents of accidental overdosage (i.e. patient reporting having taken two capsules instead of one prescribed). Such scenarios would be avoided by clear medication instructions, but if they occur, the treatment regimen will be explained again (and the participant's understanding checked) and further medical input (e.g. to see GP/ attend A&E) will be advised only if relevant.

Overdoses can be observed from the participant-completed questionnaire booklets, or via conversations directly with the participant. It is also possible that a participant's GP (or other healthcare professional) may notify the research team directly. The local centre/site should report any known overdoses to the central research team as soon as feasible, who will then notify the Sponsor within 24-hours of being made aware of the event. Such an event should be recorded on the deviation log and the appropriate study CRF.

If an S/AE is associated with the overdose (including "intentional overdoses") the CI/PI will complete the relevant report form(s) detailing the overdose information; see Section 10 (Pharmacovigilance) for reporting instructions.

10.11 Urgent safety measures (USMs)

In line with UHBW's Research Safety Reporting procedures, the Sponsor and investigator may take appropriate USMs to protect a research participant from an immediate hazard to their health and safety. This measure can be taken before seeking approval from the competent authorities (i.e. the MHRA in the UK) and ethics committees of all member states concerned.

The first action is to protect patient safety/health. Following that, the CI/Sponsor should discuss the USM by telephone as soon as it has been put in place with an MHRA safety scientist in the first

instance. This should be followed-up with written notification within 3-days* to the MHRA (UK) and local RGO (Australia), and relevant ethics committees. Notification should be in the form of a substantial amendment and describe the event, the measures taken and justification for the measures taken; further details can be found on the MHRA website (e.g. (62)).

All communication between the MHRA (UK), the RGO (Australia), the RECs, the CI/PI and the Sponsor should be documented and placed in the ISF and TMF.

**As noted on the NIHR Clinical Trials Toolkit (63), in the case of research during a pandemic period, 'Point 3 of the Medicines for Human Use (Miscellaneous Amendment) Regulations' has amended this timeline to "as soon as possible".*

10.12 Safety reporting period

The Sponsor Adverse Events Reporting Policy incorporates the requirements of the Medicine for Human Use (Clinical Trials) Regulations 2004 (UK) and the NHMRC Safety Monitoring and reporting in clinical trials involving therapeutic goods (Australia). UHBW, on behalf of the Sponsor, assumes responsibility for appropriate reporting of AEs to the regulatory authorities*. For each participant the end of safety reporting will be 7-days after the last capsule of the down titration period. Any AEs within this period will follow normal reporting procedure.

*<u>**NB:**</u> *UHBW will report any SUSARs to the MHRA on behalf of the Sponsor (UK only). The central research team (UoB and Western Australia, respectively) are responsible for all other reports to relevant regulatory authorities*.

10.13 Safety Update Reports

In addition to the expedited reporting required for SUSARs, the central research team (on behalf of the Sponsor) will submit an annual Development Safety Update Report (DSUR) to the MHRA (UK only), and relevant DSUR to REC, until the End of Trial. Trials authorised under the MHRA Notification Scheme as 'Type A', will utilise a short format DSUR. The report will be submitted within 60-days of the Clinical Trial Authorisation (CTA) of the trial each year until the trial is declared ended.

11 STATISTICS AND HEALTH ECONOMICS ANALYSIS

11.1 Sample size calculation

11.1.1 Main trial

The sample size calculations are based on the literature regarding the primary outcome (GAD-7) and experience in the NIHR-funded ADEPT study in adults with autism (3). A reduction of 2 to 3 points on the total GAD-7 score has been reported as a clinically important change (64) and based on this this trial is designed to detect a difference of 2.2 points on the GAD-7 between treatment arms at 16-weeks. The results from the ADEPT study suggest a standard deviation (SD) in GAD-7 scores of 5.7 (3) meaning the target difference equates to approximately 0.39 SD. Based on this, the study aims to recruit 306 participants, which, estimating 20% attrition at 16-weeks and a correlation of 0.37 between the baseline and 16-week GAD-7 scores (3) will yield 90% power (alpha=0.05) to estimate a mean difference of 2.2 points in GAD-7 scores between groups as randomised. By randomising 306 patients the study will have at least 80% power to detect the following differences in GAD-7 scores between treatment arms:

	**Difference in GAD-7 scores between treatment arms at 16-weeks**						
	**2.5**	**2.4**	**2.3**	**2.2**	**2.1**	**2.0**	**1.9**
Power	95.8%	94.3%	92.4%	90.1%	87.2%	83.9%	80%

11.1.2 Carer burden sub-study

No *a priori* sample size calculation was conducted for the sub-study of carers. This study will include all eligible and consenting carers of randomised STRATA participants. Should all 306 STRATA participants have a recruited carer participating in the study, the study will have 90% power (alpha-0.05) to detect a 0.4SD difference in the carer burden scale assuming 30% attrition. Should half of STRATA participants have a recruited carer participating in the study, the study will have 90% power (alpha=0.5) to detect a 0.6SD difference, assuming 30% attrition.

11.2 Statistical analysis

All analyses and reporting will be in line with CONSORT guidelines (48). Primary analyses will be based on the intention-to-treat (ITT) basis, analysing participants in the groups to which they were randomised. A full SAP will be developed by the Statisticians and central research team, and agreed by the TSC prior to undertaking analyses for the main trial.

11.2.1 Summary of baseline data

Descriptive statistics will be used to determine whether there are imbalances at baseline between treatment groups therefore informing any sensitivity analyses to be performed where appropriate additional adjustment will be performed. Continuous measures will be presented as means and SDs or medians, inter-quartile ranges, and ranges depending on their distribution. Categorical data will be presented as frequencies and proportions.

Baseline variables to be explored include those described in Section 6.7. Patient reported outcome scores based on standardised questionnaires will be calculated based on the developers' scoring manuals and missing erroneous items will be handled according to these manuals.

11.2.2 Primary outcome analysis

The primary analysis of effectiveness of the primary outcome will use linear regression to estimate an adjusted mean difference comparing GAD-7 score at 16-weeks post-randomisation between groups, adjusted for baseline values of the outcome, centre, sex, presence of intellectual disability and previous use of SSRIs (stratification/minimization variables).

Secondary analyses of the primary outcome will include a Complier Average Causal Effect (CACE) analysis (65) to investigate the efficacy of the intervention (based on treatment compliance status) for comparison with the ITT estimate of the offer of the intervention and a per protocol analysis which will account for those patients taking treatments other than that which they were allocated to. These will be conducted at 16- and 52-weeks.

11.2.3 Secondary outcome analysis

The effect of the intervention on the secondary outcomes collected at 16-, 24-, 36- and 52-weeks post-randomisation will also be examined using linear regression for continuous outcomes and logistic regression for binary outcomes adjusted for baseline values of the outcome being investigated and stratification/minimization variables.

As it is possible that adherence to treatments will decrease over the 52-week follow-up, we will describe this at each timepoint by arm as well as the use of additional or alternative medications or other treatments. A repeated measures analysis using GAD-7 outcome data collected at multiple follow up timepoints will be carried out to examine the effect of the intervention over 52-weeks.

11.2.4 Subgroup analysis

A number of pre-defined subgroup analyses will be carried out to assess the difference in treatment effect on the primary outcome according characteristics assessed at baseline. Characteristics of interest include whether the diagnosis of autism was made as an adult or child, the presence of mild intellectual disability, Attention Deficit Hyperactivity Disorder (ADHD) characteristics and severity of anxiety symptoms. Effect modification will be assessed by including an interaction term in the regression model and formal tests of interaction will be performed to test whether the treatment effect differs between these groups. As the study was not powered to detect such effects results will be interpreted with caution.

11.2.5 Adjusted analysis

All analyses will adjust for baseline values of the outcome, stratification and minimisation variables. Secondary analyses of the primary outcome will adjust for any prognostic variables showing a marked imbalance at baseline (ascertained using descriptive statistics).

11.2.6 Carer burden sub-study

Descriptive statistics will be used to describe the baseline characteristics of carers participating in the carer burden sub-study as well as the randomised participants they are caring for. These results will be used to determine whether there are imbalances at baseline between treatment groups and suggest whether appropriate additional adjustment should be performed. Continuous measures will be presented as means and SDs or medians, inter-quartile ranges, and ranges depending on their distribution. Categorical data will be presented as frequencies and proportions.

Scores based on standardised questionnaires will be calculated based on the developers' scoring manuals and missing erroneous items will be handled according to these manuals. As outlined in

section 11.5.2, the carers' EQ-5D-5L health states will be valued using the method recommended by National Institute for Health and Care Excellence (NICE) at the time of analysis; the current position statement recommends the use of the Van Hout crosswalk (66).

The effect of the intervention on the carer burden scale, carers experience scale and EQ-5D-5L collected at 16- and 52-weeks post-randomisation will be examined using linear regression adjusting for baseline values, variables used in the randomisation and any variables found to be imbalanced at baseline.

11.3 Analysis of safety endpoints

Descriptive analyses of safety endpoints will be presented at each timepoint according to treatment received. No formal comparisons will be made between groups.

11.4 Missing data

The sensitivity of the primary analysis to the impact of missing data will be investigated. The data will first be explored before a decision is made on what approach to utilise. These include exploring the amount of missingness, differences between arms, variables associated with/predictive of missingness and if reported, reasons for missingness. The approach taken to handling missing data will then depend on the assumptions about the nature of the missingness deemed to be appropriate. For example, if an assumption of Missing At Random is deemed appropriate then multiple imputation will be carried out and the primary analysis repeated using the imputed data.

11.5 Economic evaluation

11.5.1 Aim

The aim of the economic evaluation is to assess the cost-effectiveness of sertraline plus usual care compared with placebo plus usual care for the treatment of anxiety in autistic adults. Cost-effectiveness will be assessed from the perspective of the NHS and personal social services (PSS) in the UK and from a societal perspective, including productivity losses.

11.5.2 Outcomes

The primary outcome for the economic evaluation will be quality-adjusted life years derived from measurements recorded using the EQ-5D-5L health-related quality of life instrument (41) after 52-weeks of follow-up. Quality of life (via EQ-5D-5L) will be measured at baseline, 12-, 16-, 24- and 52-weeks of follow-up. Reported EQ-5D-5L health states will be valued using the method recommended by NICE at the time of analysis; the current position statement recommends the use of the Van Hout crosswalk (66). A secondary outcome will be the GAD-7 anxiety score at 52-weeks post-randomisation.

11.5.3 Cost measurement

Resources used by participants (other than sertraline) will be tracked by means of a concise bespoke patient-reported questionnaire (electronic or paper as per participant preference) administered to each group at 24- and 52-weeks post-randomisation. The resource-use questionnaire will cover hospital admissions (including length of stay), outpatient appointments, emergency department visits, primary care appointments, home visits, social care contacts and medications. In addition, participants will be asked to report time off work, if applicable. As it may be difficult for participants to accurately identify whether a contact was associated with their anxiety, information on healthcare resources used for any reason will be requested. The resource-use questionnaire (RUQ) has been developed with input from

our patient advisory group and will be tested for face validity. The RUQ will be piloted using data from the internal pilot and will be adapted for later participants if necessary.

Effectiveness and resource-use data will be taken from the UK only; valuations will be assigned to recorded resources using the most recently available standard UK sources at the time of analysis, such as the Unit Costs of Health and Social Care for primary and community care (67), the NHS reference costs for secondary care contacts (68) and the British National Formulary for prescribed medication costs (69).

11.5.4 Analysis

The analysis will be guided by a pre-specified health economics analysis plan (HEAP). The primary cost–utility analysis (CUA) will be conducted from the NHS and PSS perspective. The CUA will compare the difference in costs with the difference in QALYs between the groups after 52 weeks of follow-up; both incremental cost-effectiveness ratio and incremental net monetary benefit statistics will be calculated. A cost–consequences analysis (CCA) will also be presented from a societal perspective. The CCA will relate the differences in costs (including health and social care costs, and productivity loss) and a range of outcomes (QALYs, GAD-7, secondary outcomes from the effectiveness analysis, and carer outcomes) for each arm over 52 weeks of follow-up. As the period of follow-up is 52 weeks (one year) only, discounting of either costs or benefits is unnecessary.

Sensitivity analyses will be conducted to assess the effect of assumptions made in the analysis and uncertainty in estimates of unit costs. Uncertainty in cost-effectiveness statistics arising from patient variability will be assessed by constructing cost-effectiveness acceptability curves and by deriving confidence intervals for the net monetary benefit statistic.

12 DATA MANAGEMENT

12.1 Source data and documents

Source data for this trial will by default consist of electronic versions of preliminary screening and expression of interest forms, consent form(s), participant and carer completed questionnaires and other CRFs designed specifically for the study. However, where electronic data collection is not possible, equivalent paper documents will become the source data. Data obtained by paper will be entered onto the database as soon as practical by the centre/site research teams, and where applicable or required, by the central research team. Any paper documents containing identifiable information will be stored in a locked filing cabinet at the centre/site, which only members of the local research team have access to.

Local centre/site teams must keep a paper record of their participants (and carers involved in the nested sub-study) PIL and consent form(s) in their ISF for monitoring purposes, regardless of the method of data collection. Paper-completed CRFs should also be retained in their ISF. To enable remote-working, electronic records will suffice until a time when they can be printed and suitably filed.

12.2 Data handling

Data from all participants will be collected and retained in accordance with the UK Data Protection Act 2018 and UK General Data Protection Regulation (GDPR), which in turn comply with the Australian Privacy Principles (APP) set out in the Australian Privacy Act 1988. All trial patients (including carers involved in the nested sub-study) will be allocated a unique study I.D number during the screening process, which will remain assigned to them.

Participants from the UK will be asked to consent to their personal information and research data being stored by the University of Bristol, UK. Australian participants will be asked to consent to their personal data and research data being transferred to and stored by the University of Bristol, UK. In addition, the trial pharmacies in the UK and Australia will be securely provided with the personal details of UK and Australia-based participants, respectively; the trial pharmacies are responsible for providing participants will trial medication, and unblinding participants in an emergency. Furthermore, for randomisation purposes only, participant information (including personal details, e.g. initials, gender and date of birth) will be entered into the secure online randomisation system provided by Sealed Envelope™ (52). For the purpose of conducting the trial only, all data that are entered on to the Sealed Envelope™ system is done so via secure sockets layer (SSL) connections and stored on secure servers located in the UK and Ireland that comply with both UK and EU regulations on data privacy. User-access to the system will be managed by the central research team (UK), who will in turn generate password-protected user-accounts for authorised centre/site staff.

We will also seek participant consent to potentially collect information from information resources such as NHS Central registers, or other registries including those managed by NHS Digital (UK), and Australian equivalents. In such cases, identifiable information will be shared securely with the relevant registry (e.g. name, gender NHS number, postcode, date of birth and study I.D). Information about 'how we will use participant information' is provided to participants in the PIL, and appropriate consent will be sought.

Standardised outcome instruments will be used throughout the trial; the components and timing of follow-up measures are detailed in Section 2 (Aims and Objectives) and shown in Table 3. All participant data will be entered into and stored on password-protected Structured Query Language (SQL) databases maintained by the University of Bristol (UK). Secure access to the internet is required

for all appointments conducted remotely. For face-to-face appointments, if internet access is not available, the researcher may be able to use a study tablet that complies with sponsor information security policies, for secure offline data entry into the database. In this instance, data will be uploaded to the database once a secure internet connection is available. Alternatively, paper documents will be available, where feasible. Any data stored on laptops will be encrypted. Any information that is analysed or transferred outside the European Economic Area (EEA) will be anonymised.

Section 7.4 provides supporting details regarding the 'Data management, protection and patient confidentiality' in relation to the QRI and qualitative research data [applicable to UK only].

12.3 Database platform(s)

Full details will be outlined in the STRATA Data Management Plan. To summarise:

All administrative and clinical study data will be stored in separate REDCap instances. REDCap is a secure, web-based electronic data capture (EDC) system designed for the collection of research data. The system has been developed and supported by Vanderbilt University. BTC at the University of Bristol (UoB) has set up its own infrastructure so that all systems are hosted at and supported by UoB.

A Relational Database Management System will be used to provide integration services between administrative and clinical databases. These data will be stored here, to support the workflow of the study team. These data will not be made available for analysis. These data are stored in a SQL Server system maintained by the UoB.

The central research team (UK) will manage user-access rights to the database. This includes managing access to participant data according to the centre/site they are recruited from, and restricting access to any information that may identify the treatment received by the participant to staff who are not blinded to the allocation.

12.3.1 Administrative data

The administrative data will be kept in a secure database that is only accessible from within the UoB firewall. All users will require (at least honorary) contracts with UoB to access it.

12.3.2 Clinical data

The clinical data will be stored on a separate server to the administrative data. Anonymised clinical data is linked by a study participant I.D. Email addresses are collected as they are essential for the correct functioning of the online survey (questionnaire) feature. The 'Email Address' field is flagged as an identifier and not included in the export for the statistician, so the data set can be considered pseudonymised at export and does not need further processing.

12.4 Storage and access to data

The University of Bristol (UK) is data controller for the STRATA trial. Data will be held at the University of Bristol and will conform to the University of Bristol Data Security Policy and in Compliance with the GDPR as it applies in the UK, tailored by the Data Protection Act 2018.

Section 12.2 above includes details about data handling, including storage and access to data. In addition, for monitoring, audit and inspection purposes, monitors from the Sponsor (or delegate), persons responsible for audit, or representatives of the ethics committee or competent authority, will be granted access to source data/documents, that may include identifiable data, if requested (see Section 14.0).

The central research team (UoB-UK) will manage access rights to the data set. Prospective new users must demonstrate compliance with legal, data protection and ethical guidelines before any data are released.

12.5 Archiving and destruction of trial materials

An archiving plan will be developed for trial materials in accordance with the BTC SOPs. Data will be held in compliance with the Sponsor's standard procedures. All research data will be retained securely during the conduct of the trial. Data will be retained for *at least* 5-years after the end of the trial (15-year requirement for Australia), and at the end of the archiving period, will be destroyed by confidential means with the exception of a final dataset which will be made available for data-sharing purposes (see Section 12.6, below). Where electronic records are in use, University of Bristol (Sponsor, UK) policy will be followed. The approval of University of Bristol, as well as the trial CI and equivalent lead of the Western Australia centre, will be sought prior to destruction of the data.

Participating centres/sites will be responsible for ensuring that all study records held at their centre/site are archived appropriately when notified by the Sponsor/BTC (central research team/ UoB, UK).

12.6 Access to the final trial data set

Members of the TMG will develop a data sharing policy that is consistent with University of Bristol policy. We anticipate that anonymised trial data will be kept for future analysis and may be shared with other researchers, including those outside of the UK, EU and EEA, to enable international prospective meta-analyses. Furthermore, QRI recordings and associated data will also be securely retained for future analysis with participant consent (see QRI Data Management, Section 7.4)

The final trial data set will be stored as restricted data on the data.bris research data repository. Data will be made available to approved bona fide researchers only, after their host institution has signed a data access agreement. Details of how to request access are available at the University of Bristol's data repository website (70).

13 TRIAL MANAGEMENT

The CI will take overall responsibility for managing the various components of the trial, with the support of the Trial Manager(s), and will meet regularly (as required) with the leads for each component. The BTC, a UK Clinical Research Collaboration (UKCRC) registered trials unit, will support the delivery and conduct of the trial.

13.1 Trial management group (TMG)

The TMG will have responsibility for the day-to-day management of the trial and will report to the TSC. The TMG will meet on a regular basis with a core working group of staff having frequent progress meetings. They will link to the network of site research teams to facilitate continuous feedback and early troubleshooting of local site issues that arise. Meetings will be in person and/or by teleconference to maximise attendance.

13.2 Trial Steering committee (TSC)

The TSC will be established in conjunction with a TMG. Membership, responsibilities and reporting mechanisms of the TSC will be formalised in a TSC charter. The TSC will make recommendations/key decisions during the trial to the TMG and minutes will be sent to the funder.

The TSC will comprise of an independent chair, plus at least three others; see Table 5 below for nominations (correct at time of original submission)*. The independent members will cover expertise in (at least) statistics, trials and autism. Dheeraj Rai (CI) will also be a formal (not-voting) non-independent member of the TSC. Observers may also attend (including other members of the TMG or members of other professional bodies) at the invitation of the Chair. The TSC will meet for the first time prior to recruitment of the first participant and then at agreed intervals thereafter.

13.3 Data monitoring committee (DMC)

The DMC will meet once prior to recruitment of the first participant and convene prior to the TSC meeting to review the AE data and any other ethical aspects that arise and report to the TSC. Responsibilities and reporting mechanisms of the DMC will be formalised in a DMC charter.

The DMC will comprise of an independent chair, plus at least two others; see Table 5 below for nominations (correct at time of original submission)*. In addition, Dheeraj Rai (CI) and the Trial Manager will attend the open session only. The Senior Statistician will attend the open session only and Trial Statistician will attend both open and closed sessions.

13.4 Patient and public involvement (PPI)

An advisory group comprising adults with a diagnosis of autism has been set up to support the trial. Members of the advisory group will be involved throughout the study. This will involve autistic advisory group meetings, specific roles on the TMG, review of the protocol, participant information, consent and data collection forms and informing dissemination of the research findings to participants.

13.5 Sponsor and funding

This trial will be sponsored by University of Bristol (UoB) (UK) and The University of Western Australia (UWA) (Australia). The sponsor will be responsible for overall oversight of the trial.

The UK component of this study is funded by the National Institute for Health Research (NIHR) Health Technology Assessment Programme (Reference 127337). The views expressed are those of the

author(s) and not necessarily those of the NIHR or the Department of Health and Social Care. The Australian component of this study is funded by the NHMRC (reference APP1171206).

Table 5 TSC and DMC member composition (correct at time of time of amendment)*

Role	Name	Job Title	Institution	Expertise
**Trial Steering Committee (TSC)**				
Independent chair	Prof Nicholas Freemantle	Professor and Director	The Comprehensive Clinical Trials Unit, University College London (UCL)	Statistician
Independent member	Miss Gemma Shields	Lecturer in Healthcare Sciences	University of Manchester	Health economics and trials expertise
Independent member	Dr Sujeet Jaydeokar	Consultant Psychiatrist and Clinical Director	Cheshire and Wirral Partnership NHS Foundation Trust, University of Chester	Clinical expert
Independent member	Prof Ian Anderson	Professor Emeritus of Psychiatry	University of Manchester	Clinical, Pharmacological and trials expertise
Independent member	Mr Jorik Mol	Teacher of English as a Foreign Language	EF Education first	PPI contributor
Independent member	Dr Tom Berney	Developmental Psychiatrist (Retired)	Retired from clinical practice; current Chair of neurodevelopmental psychiatry SIG-Royal College of Psychiatrists	Autism expert
Non-Independent member	Prof Dheeraj Rai	Professor of Neurodevelopmental Psychiatry	University of Bristol, Avon and Wiltshire Partnership NHS Trust	STRATA Chief Investigator
**Data Monitoring Committee (DMC)**				
Independent chair	Dr Louise Marston	Associate Professor in Statistics,	University College London	Medical statistician
Independent member	Dr Ian Davidson	Consultant Psychiatrist	Cheshire and Wirral Partnership NHS Foundation Trust, NHS England, First Tier Tribunal Mental Health England	Clinical expert and RCPsych autism champion (until July 2020)
Independent member	Prof Christopher Dowrick	Emeritus Professor of Primary Medical Care, and GP	University of Liverpool and Aintree Park Group Practice	GP and expertise in SSRI trials

**if for any reason named members of the TSC and/or DMC are unable to continue as a member of the committee, then a suitable replacement will be sourced*

14 MONITORING, AUDIT AND INSPECTION

14.1 Monitoring

The trial will be monitored and audited in accordance with the Sponsor's policy, which is consistent with the UK Policy Framework for Health and Social Care Research and the Medicines for Human Use (Clinical Trials) Regulations (UK) and the National Health and Medical Research Council (NHMRC) (Australia). All trial related documents will be made available on request for monitoring and audit by the Sponsor, the relevant Research Ethics Committee (REC) and for inspection by MHRA and other licensing bodies.

The University of Bristol holds a Service Level Agreement (SLA) with UHBW. Under the Agreement UHBW undertakes to monitor and carry out pharmacovigilance for certain UoB sponsored studies. These activities should be carried out in accordance with the SLA, the identified risks, subsequent proposed monitoring and the trial's specific Monitoring Plan.

A Trial Monitoring Plan will be developed by the Sponsors and agreed by the TMG and CI based on the trial risk assessment which may include on-site monitoring. This will be dependent on a documented risk assessment of the trial.

The sponsor usually delegates some of the monitoring to the central research team. The following checks would be typical:

- that written informed consent has been properly documented
- that data collected are consistent with adherence to the trial protocol
- that CRFs are only being completed by authorised persons
- that S/AE recording and reporting procedures are being followed correctly
- that no key data are missing
- that data are valid
- review of recruitment rates, withdrawals and losses to follow up.

On a regular basis we will monitor the percentage of patients that meet the eligibility criteria and report the percentage of participants who consent. To assess the generalisability of the participants, the available characteristics of consenting participants and non-consenting will be compared. We will also report to the DMC if requested, preliminary data on event rates observed in the trial population: SAE rates and dropout rates.

14.2 Protocol compliance

There will be no prospective, planned deviations or waivers to the protocol. Any protocol breaches will be documented and reported to the Trial Manager, CI and Sponsor immediately (see Key Trial Contact for contact details). Information about protocol breaches will also be included in routine reports to the DMC and TSC. Protocol breaches identified by the central research team will be reported to the relevant local PI, site team, local NHS R&I and Sponsor as soon as possible. The Sponsor will determine the seriousness of the breach.

In the event of systematic protocol breaches, investigation and remedial action will be taken in liaison with the CI, DMC and the TMG.

14.3 Notification of Serious Breaches to GCP and/or the protocol

A “serious breach” is a breach which is likely to effect to a significant degree:

- the safety or physical or mental integrity of the subjects of the trial; or
- the scientific value of the trial

The Sponsor will be notified immediately of any breaches; they will determine the seriousness of the breach. The Sponsor (or authorised delegate) will report Serious Breaches to the MHRA (UK) and RECs within 7-days, and to RGO (Australia) within 72-hours of the Sponsor becoming aware of them.

15 ETHICAL AND REGULATORY CONSIDERATIONS

15.1 Governance and legislation

This trial will be conducted in accordance with:

- The Medicine for Human Use (Clinical Trial) Regulations
- Medicines and Healthcare Products Regulatory Agency (MHRA)
- International Conference on Harmonisation Good Clinical Practice (ICH-GCP) guidelines
- UK Policy Framework for Health and Social Care Research
- Data Protection Act 2018
- General Data Protection Regulation (GDPR)

Australia: The Western Australia component of the trial will also be conducted in accordance with:

- The Therapeutic Goods Act 1989
- Therapeutic Goods Regulations 1990
- PIC/S Guide to Good Manufacturing Practice for Medicinal Products
- National health and Medical Research Council (NHMRC)

Any amendments to the trial must be assessed and approved by the Sponsor prior to submission to the REC and MHRA.

Before any site can enrol participants into the trial, the CI or designee will obtain confirmation of capacity and capability (C&C) (or equivalent organisation approval) for each centre/site in-line with Health Research Authority (HRA) processes along with other documentation required for the Sponsor to grant sites a greenlight letter.

Where applicable, CI or designee will ask for confirmation from the sites' R&D departments that C&C is ongoing for relevant amendments.

This research trial will be run in accordance with ICH GCP. ICH GCP is an international ethical and scientific quality standard for designing, conducting, recording and reporting studies that involve the participation of human subjects. Compliance with this standard provides public assurance that the rights, safety and well-being of trial subjects are protected, consistent with the principles that originated in the Declaration of Helsinki and that the clinical trial data are credible.

15.2 Research Ethics Committee (REC) review and reports

Ethics review of the protocol for the trial and other trial related participant facing documents (e.g. PIL and consent forms) will be carried out by a UK and Australian RECs. Any amendments to these documents, after a favourable opinion from the REC/HRA has been given, will be submitted to the REC/HRA for approval prior to implementation.

All correspondence with the REC will be retained in the Trial Master File (TMF)/ISF. An annual progress report will be submitted to the REC within 30-days of the anniversary date on which the favourable opinion was given, and annually until the trial is declared ended. The CI will notify the REC of the end of the trial and if the trial is ended prematurely (including the reasons for the premature

termination). Within one year after the end of the trial, the CI will submit a final report with the results, including any publications/abstracts, to the REC.

ICH GCP training will be carried out by certain staff members depending on their delegated responsibilities within the trial, the level of training required will be determined according to the NIHR Delegation and Training Decision Aid. Informed consent to participate in the trial will be sought and obtained according to ICH GCP guidelines.

15.3 MHRA and TGA review and reports

UK: MHRA review of the protocol for the trial and other trial related documents relating to the IMP/placebo will be carried out by the MHRA. Clinical Trial Authorisation (CTA) will be obtained.

After the initial CTA has been approved, any amendments (which effect the safety (physical or mental integrity) of the participants, the scientific value of the study, the conduct or management of the study or the quality or safety of any IMP) will constitute a substantial amendment and a request to the MHRA for approval will be submitted.

All correspondence with the MHRA will be retained in the TMF/ISF.

In addition to the expedited reporting required for SUSARs, a DSUR will be submitted to the MHRA and NHS REC, once a year throughout the clinical trial or on request until the end of the trial is declared. The annual report should take into account all new available safety information received during the reporting period and assess the safety of subjects included in the study.

The sponsor will submit an end of trial summary results to EudraCT as per the commission's guidelines on posting and publication of result-related information within one year of the end of study declaration being submitted.

Australia: Equivalent to the UK's MHRA and CTA is the Therapeutic Goods Administration (TGA) and Clinical Trial Notification (CTN), respectively. The Australian Sponsor (UWA) will submit and obtain CTN.

After the initial CTN has been approved, any amendments (which effect the safety (physical or mental integrity) of the participants, the scientific value of the study, the conduct or management of the study or the quality or safety of any IMP) will constitute a substantial amendment and a request to the TGA for approval will be submitted.

All correspondence with the TGA will be retained in the TMF/ISF.

The TGA does not require expedited reporting for SUSARs, or annual safety reports; reporting is only required by the HREC, which the local research team are responsible for.

15.4 Peer review

The proposal for this trial has been peer-reviewed through the NIHR HTA (UK) and NHMRC (Australia) peer-review process, which includes independent expert and lay reviewers.

15.5 Poor quality data

The quality of the trial data will be monitored throughout the trial and data completeness will be reported to the TSC, and any cause for concern over data quality will be highlighted and an action plan put in place.

15.6 Financial and other competing interests for the chief investigator, PIs at each site and committee members for the overall trial management

The research team and all PIs must disclose any ownership interests that may be related to products, services, or interventions considered for use in the trial or that may be significantly affected by the trial. Competing interests will be reported in all publications and in the final report.

15.7 Indemnity

The necessary trial insurance is provided by the Sponsors.

16 DISSEMINATION POLICY

An engagement plan will be produced with the TMG, collaborators and the autistic advisory group.

The outputs of this work will include academic papers in leading peer reviewed journals alongside the full report published in the NIHR Journal's library. We will also present the findings of our work at relevant international conferences including INSAR, Autism Europe and the Royal College of Psychiatrists International congress and national conferences including the Autistica and National Autistic Society conferences and the British Association for Psychopharmacology conferences. With the help of Autistica, we will also organise a workshop on anxiety in autism where we will present the results of this study. The results of this work are likely to influence the updates of the NICE guidelines for autism in adults, the Maudsley Prescribing guidelines, the British Association for Psychopharmacology guidelines on autism and other relevant national and international guidelines.

We will closely work with our advisory group and Autistica and other autism charities (e.g. National Autistic Society, Ambitious about Autism in the UK; Autism Association Western Australia in Perth) who will help popularise our project and help us engage with stakeholders. We will aim to disseminate the findings widely to the autism community, health care providers, policymakers and the public through presentations, roadshows and blogs.

We have strong links with local, national and international networks of clinicians which we will use to disseminate our outputs directly through talks, educational events and clinical conferences. Furthermore, our team has extensive experience of media contributions, including local, national and international television, radio and press contributions, as well as public lectures and writing for the public (e.g. the Conversation).

We will work with PolicyBristol (71), a specialist team at the University of Bristol (UK) which aims to bring together diverse policy-relevant work from across the University to make it more accessible to research users from outside the academic community. PolicyBristol helps translate research findings into policy messages, and then disseminates these to the relevant target audiences in the form of policy briefings or reports. We will produce briefing notes to disseminate to key agencies and stakeholders, such as the MHRA, Royal Colleges of Psychiatrists, Clinical Commissioning Groups and the British Association for Psychopharmacology, and the Royal Australian and New Zealand College of Psychiatrists and the College of General Practitioners. We will also engage with the National Disability Insurance Scheme (NDIS) which provides funding and management to most people with Autism in Australia.

On completion of the trial, a final report will be prepared for the Funder (NHR HTA and NHMRC) and once approved made publicly available on their website.

A study Twitter account and website will be set up to keep interested participants, managers and policy makers up-to-date with trial progress and results.

17 SIGNATURE PAGE

The undersigned confirm that the following protocol has been agreed and accepted and that the Chief Investigator agrees to conduct the trial in compliance with the approved protocol and will adhere to the principles outlined in UK Policy Framework for Health and Social Care Research, the Sponsor's SOPs, and other regulatory requirements as amended.

I agree to ensure that the confidential information contained in this document will not be used for any other purpose other than the evaluation or conduct of the clinical investigation without the prior written consent of the Sponsor.

I also confirm that I will make the findings of the study publicly available through publication or other dissemination tools without any unnecessary delay and that an honest accurate and transparent account of the study will be given; and that any discrepancies from the study as planned in this protocol will be explained.

For and on behalf of the **Study Sponsor:**

Signature:

.... Date: ....../......./.............

Name (please print):

....

Chief Investigator:

Signature:

.... Date: ....../......./.............

Name (please print):

....

Senior Statistician:

Signature:

.... Date: ....../......./.............

Name (please print):

....

18 REFERENCES

1. Lai MC, Lombardo MV, Baron-Cohen S. Autism. Lancet. 2014;383(9920):896-910.
2. Buescher AV, Cidav Z, Knapp M, Mandell DS. Costs of autism spectrum disorders in the United Kingdom and the United States. JAMA Pediatr. 2014;168(8):721-8.
3. Russell A, Cooper K, Barton S, Ensum I, Gaunt D, Horwood J, et al. Protocol for a feasibility study and randomised pilot trial of a low-intensity psychological intervention for depression in adults with autism: the Autism Depression Trial (ADEPT). BMJ Open. 2017;7(12):e019545.
4. Lai MC, Baron-Cohen S. Identifying the lost generation of adults with autism spectrum conditions. Lancet Psychiatry. 2015;2(11):1013-27.
5. Selten JP, Lundberg M, Rai D, Magnusson C. Risks for nonaffective psychotic disorder and bipolar disorder in young people with autism spectrum disorder: a population-based study. JAMA Psychiatry. 2015;72(5):483-9.
6. Rai D, Heuvelman H, Dalman C, Culpin I, Lundberg M, Carpenter P, et al. Association Between Autism Spectrum Disorders With or Without Intellectual Disability and Depression in Young Adulthood. JAMA Netw Open. 2018;1(4):e181465.
7. Hirvikoski T, Mittendorfer-Rutz E, Boman M, Larsson H, Lichtenstein P, Bolte S. Premature mortality in autism spectrum disorder. Br J Psychiatry. 2016;208(3):232-8.
8. Schendel DE, Overgaard M, Christensen J, Hjort L, Jorgensen M, Vestergaard M, et al. Association of Psychiatric and Neurologic Comorbidity With Mortality Among Persons With Autism Spectrum Disorder in a Danish Population. JAMA Pediatr. 2016;170(3):243-50.
9. Buck TR, Viskochil J, Farley M, Coon H, McMahon WM, Morgan J, et al. Psychiatric comorbidity and medication use in adults with autism spectrum disorder. J Autism Dev Disord. 2014;44(12):3063-71.
10. Lever AG, Geurts HM. Psychiatric Co-occurring Symptoms and Disorders in Young, Middle-Aged, and Older Adults with Autism Spectrum Disorder. J Autism Dev Disord. 2016;46(6):1916-30.
11. Hollocks MJ, Lerh JW, Magiati I, Meiser-Stedman R, Brugha TS. Anxiety and depression in adults with autism spectrum disorder: a systematic review and meta-analysis. Psychol Med. 2018:1-14.
12. Kerns CM, Kendall PC, Berry L, Souders MC, Franklin ME, Schultz RT, et al. Traditional and atypical presentations of anxiety in youth with autism spectrum disorder. J Autism Dev Disord. 2014;44(11):2851-61.
13. Nimmo-Smith V, Heuvelman H, Dalman C, Lundberg M, Idring S, Carpenter P, et al. Anxiety Disorders in Adults with Autism Spectrum Disorder: A Population-Based Study. J Autism Dev Disord. 2020;50(1):308-18.
14. Ormel J, Koeter MW, van den Brink W, van de Willige G. Recognition, management, and course of anxiety and depression in general practice. Arch Gen Psychiatry. 1991;48(8):700-6.
15. Vermani M, Marcus M, Katzman MA. Rates of detection of mood and anxiety disorders in primary care: a descriptive, cross-sectional study. Prim Care Companion CNS Disord. 2011;13(2).
16. Baldwin DS, Anderson IM, Nutt DJ, Allgulander C, Bandelow B, den Boer JA, et al. Evidence-based pharmacological treatment of anxiety disorders, post-traumatic stress disorder and obsessive-compulsive disorder: a revision of the 2005 guidelines from the British Association for Psychopharmacology. J Psychopharmacol. 2014;28(5):403-39.
17. Taylor D, Paton C, Kapur S, South London and Maudsley NHS Trust. The Maudsley prescribing guidelines in psychiatry. 12th edition. ed. Chichester, West Sussex ; Hoboken, NJ: John Wiley & Sons Inc.; 2015. p. p.
18. Scott A, Davidson A, Palmer K. Antidepressant drugs in the treatment of anxiety disorders. Advances in Psychiatric Treatment. 2001;7(4):275-82.
19. Ballenger JC. Remission rates in patients with anxiety disorders treated with paroxetine. J Clin Psychiatry. 2004;65(12):1696-707.
20. Londborg PD, Wolkow R, Smith WT, DuBoff E, England D, Ferguson J, et al. Sertraline in the treatment of panic disorder. A multi-site, double-blind, placebo-controlled, fixed-dose investigation. Br J Psychiatry. 1998;173:54-60.
21. Muller CL, Anacker AMJ, Veenstra-VanderWeele J. The serotonin system in autism spectrum disorder: From biomarker to animal models. Neuroscience. 2016;321:24-41.
22. Gabriele S, Sacco R, Persico AM. Blood serotonin levels in autism spectrum disorder: a systematic review and meta-analysis. Eur Neuropsychopharmacol. 2014;24(6):919-29.

23. Buchsbaum MS, Hollander E, Haznedar MM, Tang C, Spiegel-Cohen J, Wei TC, et al. Effect of fluoxetine on regional cerebral metabolism in autistic spectrum disorders: a pilot study. Int J Neuropsychopharmacol. 2001;4(2):119-25.
24. Hollander E, Soorya L, Chaplin W, Anagnostou E, Taylor BP, Ferretti CJ, et al. A double-blind placebo-controlled trial of fluoxetine for repetitive behaviors and global severity in adult autism spectrum disorders. Am J Psychiatry. 2012;169(3):292-9.
25. McDougle CJ, Naylor ST, Cohen DJ, Volkmar FR, Heninger GR, Price LH. A double-blind, placebo-controlled study of fluvoxamine in adults with autistic disorder. Arch Gen Psychiatry. 1996;53(11):1001-8.
26. Howes OD, Rogdaki M, Findon JL, Wichers RH, Charman T, King BH, et al. Autism spectrum disorder: Consensus guidelines on assessment, treatment and research from the British Association for Psychopharmacology. J Psychopharmacol. 2018;32(1):3-29.
27. Austistica. Your Research Priotities 2016 [Available from: https://www.autistica.org.uk/our-research/our-research/your-research-priorities.
28. Spitzer RL, Kroenke K, Williams JB, Lowe B. A brief measure for assessing generalized anxiety disorder: the GAD-7. Arch Intern Med. 2006;166(10):1092-7.
29. NHS England. Adult Improving Access to Psychological Therapies programme [Available from: https://www.england.nhs.uk/mental-health/adults/iapt/.
30. Kroenke K, Spitzer RL, Williams JB, Monahan PO, Lowe B. Anxiety disorders in primary care: prevalence, impairment, comorbidity, and detection. Ann Intern Med. 2007;146(5):317-25.
31. Lewis G, Mulligan J, Wiles N, Cowen P, Craddock N, Ikeda M, et al. Polymorphism of the 5-HT transporter and response to antidepressants: randomised controlled trial. Br J Psychiatry. 2011;198(6):464-71.
32. Salaminios G, Duffy L, Ades A, Araya R, Button KS, Churchill R, et al. A randomised controlled trial assessing the severity and duration of depressive symptoms associated with a clinically significant response to sertraline versus placebo, in people presenting to primary care with depression (PANDA trial): study protocol for a randomised controlled trial. Trials. 2017;18(1):496.
33. Kroenke K, Spitzer RL. The PHQ-9: A new depression diagnostic and severity measure. Psychiatric Annals. 2002;32(9):509-15.
34. Connor KM, Davidson JR, Churchill LE, Sherwood A, Foa E, Weisler RH. Psychometric properties of the Social Phobia Inventory (SPIN). New self-rating scale. Br J Psychiatry. 2000;176:379-86.
35. Cadman T, Spain D, Johnston P, Russell A, Mataix-Cols D, Craig M, et al. Obsessive-Compulsive Disorder in Adults with High-Functioning Autism Spectrum Disorder: What Does Self-Report with the OCI-R Tell Us? Autism Res. 2015;8(5):477-85.
36. Spitzer RL, Kroenke K, Williams JBW, Group atPHQPCS. Validation and Utility of a Self-report Version of PRIME-MDThe PHQ Primary Care Study. JAMA. 1999;282(18):1737-44.
37. Barrett SL, Uljarevic M, Baker EK, Richdale AL, Jones CR, Leekam SR. The Adult Repetitive Behaviours Questionnaire-2 (RBQ-2A): A Self-Report Measure of Restricted and Repetitive Behaviours. J Autism Dev Disord. 2015;45(11):3680-92.
38. Wiles NJ, Mulligan J, Peters TJ, Cowen PJ, Mason V, Nutt D, et al. Severity of depression and response to antidepressants: GENPOD randomised controlled trial. British Journal of Psychiatry. 2012;200(2):130-6.
39. Lewis G, Duffy L, Ades A, Amos R, Araya R, Brabyn S, et al. The clinical effectiveness of sertraline in primary care and the role of depression severity and duration (PANDA): a pragmatic, double-blind, placebo-controlled randomised trial. The Lancet Psychiatry. 2019;6(11):903-14.
40. McConachie H, Mason D, Parr JR, Garland D, Wilson C, Rodgers J. Enhancing the Validity of a Quality of Life Measure for Autistic People. J Autism Dev Disord. 2018;48(5):1596-611.
41. EuroQol - a new facility for the measurement of health-related quality of life. Health Policy. 1990;16(3):199-208.
42. Macera CA, Eaker ED, Jannarone RJ, Davis DR, Stoskopf CH. A measure of perceived burden among caregivers. Eval Health Prof. 1993;16(2):205-11.
43. Al-Janabi H, Coast J, Flynn TN. What do people value when they provide unpaid care for an older person? A meta-ethnography with interview follow-up. Soc Sci Med. 2008;67(1):111-21.
44. Al-Janabi H, Flynn TN, Coast J. Estimation of a preference-based carer experience scale. Med Decis Making. 2011;31(3):458-68.
45. Goranitis I, Coast J, Al-Janabi H. An investigation into the construct validity of the Carer Experience Scale (CES). Qual Life Res. 2014;23(6):1743-52.

46. Avery KN, Williamson PR, Gamble C, O'Connell Francischetto E, Metcalfe C, Davidson P, et al. Informing efficient randomised controlled trials: exploration of challenges in developing progression criteria for internal pilot studies. BMJ Open. 2017;7(2):e013537.
47. Wilson C, Rooshenas L, Paramasivan S, Elliott D, Jepson M, Strong S, et al. Development of a framework to improve the process of recruitment to randomised controlled trials (RCTs): the SEAR (Screened, Eligible, Approached, Randomised) framework. Trials. 2018;19(1):50.
48. Schulz KF, Altman DG, Moher D, Group C. CONSORT 2010 Statement: Updated Guidelines for Reporting Parallel Group Randomized Trials. Annals of Internal Medicine. 2010;152(11):726-32.
49. Adult Autism Spectrum Cohort - UK (AASC-UK). Adult Autism Spectrum Cohort - UK [Available from: https://research.ncl.ac.uk/adultautismspectrum/.
50. Austistica. Discover network for researchers [Available from: https://www.autistica.org.uk/our-research/discover-network.
51. Telethon Kids Institute. Western Australian Register for Autism Spectrum Disorders 2020 [Available from: https://autism.telethonkids.org.au/autismregister/.
52. Sealed Envelope Ltd. Randomisation and online databases for clinical trials 2001-2020 [Available from: https://www.sealedenvelope.com/.
53. Robinson KA, Dennison CR, Wayman DM, Pronovost PJ, Needham DM. Systematic review identifies number of strategies important for retaining study participants. Journal of clinical epidemiology. 2007;60(8):757-65.
54. Edwards PJ, Roberts I, Clarke MJ, Diguiseppi C, Wentz R, Kwan I, et al. Methods to increase response to postal and electronic questionnaires. Cochrane Database Syst Rev. 2009(3):MR000008.
55. Robertson A, Stanfield A, Watt J, Barry F, Day M, Cormack M, et al. The experience and impact of anxiety in autistic adults: A thematic analysis. Research in Autism Spectrum Disorders. 2018;46(2):8-18.
56. McNamara R, Randell E, Gillespie D, Wood F, Felce D, Romeo R, et al. A pilot randomised controlled trial of community-led ANtipsychotic Drug REduction for Adults with Learning Disabilities. Health Technol Assess. 2017;21(47):1-92.
57. Donovan JL, Rooshenas L, Jepson M, Elliott D, Wade J, Avery K, et al. Optimising recruitment and informed consent in randomised controlled trials: the development and implementation of the Quintet Recruitment Intervention (QRI). Trials. 2016;17(1):283.
58. Donovan JL, Lane JA, Peters TJ, Brindle L, Salter E, Gillatt D, et al. Development of a complex intervention improved randomization and informed consent in a randomized controlled trial. J Clin Epidemiol. 2009;62(1):29-36.
59. Donovan JL, Paramasivan S, de Salis I, Toerien M. Clear obstacles and hidden challenges: understanding recruiter perspectives in six pragmatic randomised controlled trials. Trials. 2014;15:5.
60. Rooshenas L, Lj S, Jm B, Ca R, Km T, S H, et al. The Quintet Recruitment Intervention supported five randomized trials to recruit to target: a mixed-methods evaluation. J Clin Epidemiol. 2018.
61. British National Formulary (National Institute for Health and Care Excellence; NICE). Sertraline 2020 [Available from: https://bnf.nice.org.uk/drug/sertraline.html.
62. MHRA. Clinical trials for medicines: manage your authorisation, report safety issues 2019 [Available from: https://www.gov.uk/guidance/clinical-trials-for-medicines-manage-your-authorisation-report-safety-issues#l11
63. NIHR Clinical Trials Toolkit. Urgent Safety Measures 2020 [Available from: http://www.ct-toolkit.ac.uk/routemap/urgent-safety-measures/.
64. Kroenke K, Wu J, Yu Z, Bair MJ, Kean J, Stump T, et al. Patient Health Questionnaire Anxiety and Depression Scale: Initial Validation in Three Clinical Trials. Psychosom Med. 2016;78(6):716-27.
65. Dunn G, Maracy M, Tomenson B. Estimating treatment effects from randomized clinical trials with noncompliance and loss to follow-up: the role of instrumental variable methods. Stat Methods Med Res. 2005;14(4):369-95.
66. NICE. National Institute for Health and Care Excellence (NICE) Position statement on the EQ-5D-5L value set for England (updated October 2019) 2019 [Available from: https://www.nice.org.uk/about/what-we-do/our-programmes/nice-guidance/technology-appraisal-guidance/eq-5d-5l.
67. Unit Costs of Health and Social Care 2017 [Internet]. Personal Social Services Research Unit, University of Kent. 2017.
68. NHS Reference Costs 2018 [Available from: https://improvement.nhs.uk/resources/reference-costs/.

69. British Medical Association., Joint Formulary Committee., Pharmaceutical Society of Great Britain. British national formulary. London: British Medical Association.
70. University of Bristol. The data.bris Research Data Repository [Available from: https://data.bris.ac.uk/data/.
71. PolicyBristol. PolicyBristol [Available from: http://www.bristol.ac.uk/policybristol/.
72. McDermott MM, Newman AB. Preserving Clinical Trial Integrity During the Coronavirus Pandemic. JAMA. 2020;323(21):2135-6.

APPENDIX 1 STRATA Dose titration and stopping instructions

Table 6 STRATA Dose titration

STRATA DOSE TITRATION				
	**Dose of sertraline**	**Take for**	**Safety check / follow up**	**Dispensing**
**Week 0**	25mg	2-weeks	-	Send 1 starter pack 70 caps of 25mg each
**Week 2**	All patients instructed to increase dose to 50mg (2*25)	4-weeks	2-week check (been on 25mg for 2-weeks, if undesirable symptoms could continue only 25mg)	-
**Week 4**	-	-	4-week check (been on 50mg for 2 weeks, discuss if wish to increase or continue 50mg or reduce/stop)	Send 1 pack 70 caps of 50mg each
**Week 6**	Increase dose to 100mg (2*50) if agreed at week 4 check	4-weeks	-	-
**Week 8**	-	-	8-week check (if relevant, been on 100mg for 2-weeks, discuss if wish to increase to 150mg, stay on same dose, or reduce to 50mg)	Send 1 pack 70 caps of 50mg each
**Week 10**	For those who agree, increase dose to 150mg (3*50)<br><br>Else continue 100mg for 52 weeks or reduce to 50mg as appropriate	4-weeks	-	-
**Week 12**	-	-	12-week check (if participant has been on 150mg for 2-weeks, discuss if wish to increase to 200mg, stay on same dose, or reduce to 150mg). Same for other dosage as relevant	Dispense according to optimal dose as per prescription
**Week 14**	Those who have increased dose will now be on max dose of 200mg. Others will already be on the optimal dose ahead of this and should continue	-	-	-
**Week 16**	50mg (2*25) to 200mg (4*50)	-	Primary outcome	-

[A]A brief safety check will also take place at 36-weeks post-randomisation to inform dose titration (e.g. continue same dose, increase or decrease; see Section 6.8).

STRATA STOPPING INSTRUCTIONS

Applicable from week 52 post-randomisation, or at time when participant withdraws from treatment if earlier.

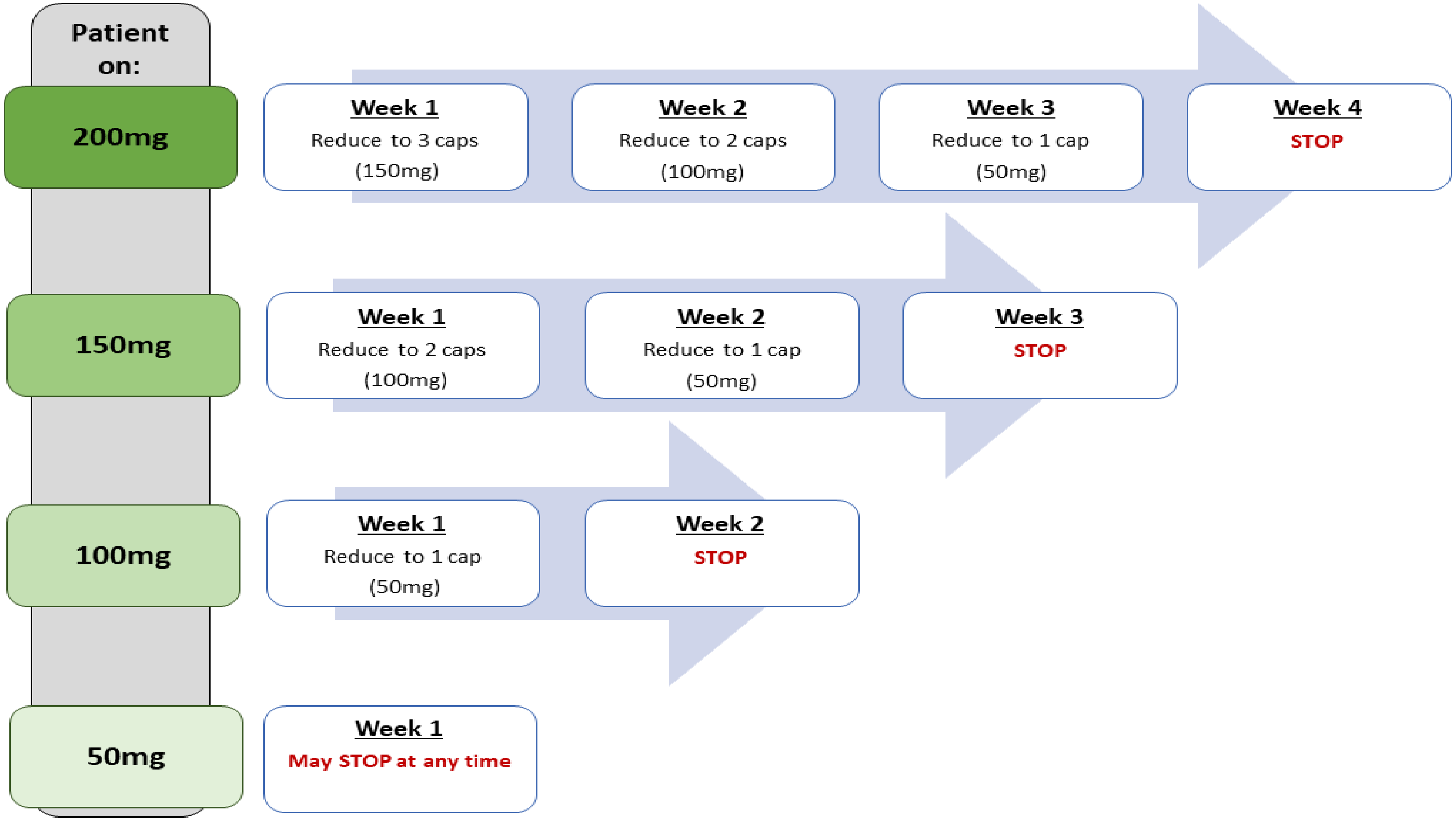

Figure 9 STRATA Stopping instructions

APPENDIX 2 COVID-19 Considerations

Research, including randomised controlled trials (RCTs), provides essential high-quality evidence that guides medical advancements around the world, influencing the quality of health and lives of all. Due to the coronavirus pandemic (COVID-19), research in the UK, Australia and globally has had to respond to the various COVID-19 mitigation efforts (e.g. societal lockdowns, social distancing and shielding measures) and unprecedented demands on healthcare professionals and resources. This has resulted in many research projects that are not related to the pandemic, being paused or in some cases, halted altogether. It is imperative that we consider how research can continue during, and beyond, COVID-19, or equivalent scenarios. Here, we summarise key considerations and actions taken to enable the continuation of STRATA, in both the UK and Australia, which are based on (rapidly evolving) guidance from relevant research bodies (e.g. NIHR, HRA, MHRA, NHMRC, TGA), supporting literature (e.g. (72)) and preliminary interview data.

Trial conduct considerations

Importantly, at the time the pandemic struck in early 2020, members of the central research team had been working with our qualitative researchers to carry out a qualitative study (Autistic people and RCTs - the APRiCoT study). APRiCoT set out to understand the views of autistic adults (recruited via the charity Autistica and social media and interviewed using remote methods) in relation to randomisation and processes involved in RCTs to help protocol development and embedding measures to optimise recruitment to STRATA. Following the pandemic, the team received an ethics amendment to extend the scope of the APRiCoT study to understand the impact of COVID-19 and social distancing measures on future research participation, discussing specific examples of STRATA as an RCT. Preliminary observations (not to be cited until peer reviewed) from the data collected so far (42 detailed 1:1 interviews for the APRiCoT study, which includes 26 with COVID-19 themes) are that research into treatment for anxiety is important in this population, particularly in the aftermath of the pandemic where many peoples' daily routines have been disrupted. Additionally, participants have strongly suggested making participation in research more accessible (regardless of the pandemic) by increasing the emphasis on remote pathways for research delivery (such as consent and data collection). As a result, the team have embedded some of these processes (adaptations) in STRATA, and will incorporate key information within relevant participant materials/documents to provide assurance to them regarding their health and safety (this includes when they are considering taking part, as well as throughout participation).

Adapting consent and data collection: We anticipate that the majority of Baseline appointments (and other study points of contact and assessments) will be conducted via Sponsor-approved video-conferencing (i.e. to reduce risk of potential exposure to COVID-19 and/or due to a requirement for remote contact and data collection by the potential participant). Alternative methods of communication (e.g. other remote methods and/or face-to-face visits) will be considered and facilitated, where feasible. Online eConsent and questionnaires will be available, in addition to more traditional methods such as paper copies or completed over the telephone. In addition, given the increased pragmatic nature of STRATA, we anticipate the need for flexibility surrounding the timings of some points of contact and assessment (e.g. the brief safety checks), so have provided acceptable timings for these (e.g. +/- 1-week). From the researcher perspective, direct data entry into the secure online database minimises data protection concerns. The study also proposes collecting back up 'best contact' alternatives (e.g. the contact details of others including carer/ other family member, where applicable) in case contact with the participant is lost.

Furthermore, when face-to-face appointments are “permitted”, we recognise they bring about higher risk and may not (always) be feasible. To mitigate such risks, STRATA offers flexibility around assessment and communication methods as noted above (and throughout the protocol). If face-to-face appointments are requested, research staff should assess feasibility and follow the organisation guidance at the time regarding face-to-face contact, including, for example, use of personal protective equipment (PPE) and cleaning procedures. Research staff would be encouraged to help participants avoid situations where risk of exposure may be greater (e.g. public transport) and remind participants that they have a choice of contact and data collection methods to suit their needs and preferences.

Intervention / IMP considerations

McDermott and Newman (2020), amongst several suggestions, encouraged trials to consider whether the intervention may interact with COVID-19 infection, whether trial medication should stop if a patient contracts (symptoms of) COVID-19, and whether there are increased supply issues during the pandemic that could impact the trial? (72). Within STRATA, to our knowledge, no interaction between the study medication (intervention) and the COVID-19 infection is anticipated, therefore treatment should continue as prescribed if a participant contracts the infection (unless a treating healthcare professional advises otherwise for a clinical reason), and Eramol, who are responsible for the supply of the study Investigational Medicinal Product (IMP), do not foresee any supply issues. In addition, we propose that study medication is posted directly to a participant’s home, helping those who may be “shielding”, for example. Collectively, it is feasible to proceed with the intervention as proposed.

Urgent Safety Measures (USMs)

USMs typically need to be followed-up with written notification within 3-days to the Medicines and Healthcare Products Regulatory Agency (MHRA), and relevant ethics committee. Research guidance notes that in the case of research during a pandemic period, *‘Point 3 of the Medicines for Human Use (Miscellaneous Amendment) Regulations’* has amended this timeline to “as soon as possible” (63); the research team have included this information in the protocol to aid staff members if/where necessary.

APPENDIX 3 Amendments to Protocol

Amendment No.	Protocol Version No. (UK)	Protocol Version No. (AUS)[2]	Submission date (UK)	Approval date (UK)	Details of Changes made
SA01[3]	2.0	2.0	08 July 2021	09 July 2021	Minor changes and clarifications to the protocol.
NSA02	No changes to protocol.				
SA03	3.0	3.0	04 November 2021	17 November 2021	Minor alternations to the protocol, including GP Safety Check, pharmacovigilance, and secondary outcomes
NSA04	No changes to protocol.				
SA05	4.0	4.0	22 December 2021	26 January 2022	Updates to the protocol regarding non-medical clinical prescribers and GP safety check process.
NSA06, NSA07, SA08, NSA09	No changes to protocol.				
NSA10	5.0	5.0	06 December 2022	06 December 2022	Minor change to the protocol to correct an administrative error regarding the Expression of Interest (EOI) process.
SA11	6.0	6.0	15 December 2022	24 February 2023, Reissue 14 April 2023 due to typographical error	Amendments to protocol to reflect updated recruitment and study timeline dates agreed with funder, additional manufacturing address and name change of IMP supplier and other small changes. Clarification of exclusion criteria regarding prior

[2] SA refers to Substantial Amendment, NSA refers to Non-Substantial Amendment. This is followed by the number of the amendment itself.
[3] All corresponding changes to the protocol were submitted and approved by both HREC and RGO in Australia after UK approval had been received.

					antidepressant use and psychosis/bipolar diagnoses.
SA12 (AWP only)	No changes to protocol.				
NSA13	7.0	7.0 (yet to be submitted)	13-Sep-2023	Pending	Amendments to protocol to update Data Monitoring & Ethics Committee members (change of two members), clarification of a secondary objective, small change to subgroup analysis, and minor administrative changes.
