# Supplementary material for: Sertraline for anxiety in adults with a diagnosis of autism (STRATA): study protocol for a pragmatic, multicentre, double-blind, placebo-controlled randomised controlled trial
Source: Trials. 2024 Jan 11;25:37. doi: 10.1186/s13063-023-07847-3 (PMC10782796; doi:10.1186/s13063-023-07847-3)
Supplement: Supplementary file 2 — Additional file 2. Participant consent form. [file 13063_2023_7847_MOESM2_ESM.docx]

**PARTICIPANT CONSENT FORM**

| **Chief Investigator:** | Dr Dheeraj Rai | | | | | **Principal Investigator:** | | | | | [INSERT LOCAL PI NAME] | | | | | | | | | | | | | | | | | | | | | | | |  |
| --- | --- | --- | --- | --- | --- | --- | --- | --- | --- | --- | --- | --- | --- | --- | --- | --- | --- | --- | --- | --- | --- | --- | --- | --- | --- | --- | --- | --- | --- | --- | --- | --- | --- | --- | --- |
|  |  | | | | |  | | | | |  | | | | | | | | | | | | | | | | | | | | | | | |  |
| **Participant I.D number:** | |  |  | | | |  |  |  |  | | | |  | | | |  | | | | | | **Please INITIAL each box below** | | | | | | | | | | | |
|  | | | | | | | | | | | | | | | | |  | | | | | | | | | | | | | | | | | |  |
| 1. **I confirm** that I have read the Participant Information Leaflet (version XX, XXXXX2020) for the above study. I have had the opportunity to consider the information, ask questions, and have had these answered satisfactorily. | | | | | | | | | | | | | | | | | | | | | | | | | |  | |  | | | | |  | |  |
|  |  |  |  |  |  |  |  |  |  |  |  |  |  |  |  |  |  |  |  |  |  |  |  |  |  |  |  | *Initials* | | | | |  |  |  |
|  |  |  |  |  |  |  |  |  |  |  |  |  |  |  |  |  |  |  |  |  |  |  |  |  |  |  |  |  | | | | |  |  |  |
| 1. **I understand** that my participation in this study is voluntary and that I am free to withdraw at any time without giving a reason and without my medical care or legal rights being affected. | | | | | | | | | | | | | | | | | | | | | | | | | |  | |  | | | | |  | |  |
|  |  |  |  |  |  |  |  |  |  |  |  |  |  |  |  |  |  |  |  |  |  |  |  |  |  |  |  | *Initials* | | | | |  |  |  |
|  |  |  |  |  |  |  |  |  |  |  |  |  |  |  |  |  |  |  |  |  |  |  |  |  |  |  |  |  | | | | |  |  |  |
| 1. **I understand** that relevant sections of my medical notes and data collected during the study may be looked at by individuals from the research team, University Hospitals Bristol and Weston NHS Foundation Trust (UHBW) or the regulatory authorities, where it is relevant to my taking part in this research. I give permission for these individuals to have access to the relevant parts of my medical records. | | | | | | | | | | | | | | | | | | | | | | | | | |  | |  | | | | |  | |  |
|  |  |  |  |  |  |  |  |  |  |  |  |  |  |  |  |  |  |  |  |  |  |  |  |  |  |  |  | *Initials* | | | | |  |  |  |
|  |  |  |  |  |  |  |  |  |  |  |  |  |  |  |  |  |  |  |  |  |  |  |  |  |  |  |  |  | | | | |  |  |  |
| 1. **I agree** to my General Practitioner (GP/GP Practice) being informed of my participation in the study, and to them being involved in the study, including any necessary exchange of information about me between my GP and the research team. | | | | | | | | | | | | | | | | | | | | | | | | | |  | |  | | | | |  | |  |
|  |  |  |  |  |  |  |  |  |  |  |  |  |  |  |  |  |  |  |  |  |  |  |  |  |  |  |  | *Initials* | | | | |  |  |  |
|  |  |  |  |  |  |  |  |  |  |  |  |  |  |  |  |  |  |  |  |  |  |  |  |  |  |  |  |  | | | | |  |  |  |
| 1. **I understand** that the information collected about me may be used anonymously to support other research in the future and may be shared anonymously with other researchers. | | | | | | | | | | | | | | | | | | | | | | | | | |  | |  | | | | |  | |  |
|  |  |  |  |  |  |  |  |  |  |  |  |  |  |  |  |  |  |  |  |  |  |  |  |  |  |  |  | *Initials* | | | | |  |  |  |
|  |  |  |  |  |  |  |  |  |  |  |  |  |  |  |  |  |  |  |  |  |  |  |  |  |  |  |  |  | | | | |  |  |  |
| 1. **I agree** that information about me will be shared with Sealed Envelope^TM^, the company who provide the software that helps to enable the process of randomisation (e.g. study identification number, initials, site, GAD-7 score, gender, date of birth, presence of intellectual disability, and medication use for anxiety or depression), and University Hospitals Bristol and Weston NHS Foundation Trust (UHBW), the pharmacy providing you with the study medication (e.g. study identification number, name, NHS number, date of birth, postal address). | | | | | | | | | | | | | | | | | | | | | | | | | |  | |  | | | | |  | |  |
|  |  |  |  |  |  |  |  |  |  |  |  |  |  |  |  |  |  |  |  |  |  |  |  |  |  |  |  | *Initials* | | | | |  |  |  |
|  |  |  |  |  |  |  |  |  |  |  |  |  |  |  |  |  |  |  |  |  |  |  |  |  |  |  |  |  | | | | |  |  |  |
| 1. **I agree** to information from NHS Digital/Information Services Division Scotland (ISD)/Patient Episode Database for Wales (PEDW) and mortality data being used by the study team and am aware that my full name, gender, NHS number, postcode, date of birth and study number will be shared with them so that they can send the information required, as described in the Participant Information Leaflet, back to the University of Bristol. | | | | | | | | | | | | | | | | | | | | | | | | | |  | |  | | | | |  | |  |
|  |  |  |  |  |  |  |  |  |  |  |  |  |  |  |  |  |  |  |  |  |  |  |  |  |  |  |  | *Initials* | | | | |  |  |  |
|  |  |  |  |  |  |  |  |  |  |  |  |  |  |  |  |  |  |  |  |  |  |  |  |  |  |  |  |  | | | | |  |  |  |
| 1. **I understand** that my personal and research data will be kept for at least 5 years after the end of the study and held confidentially and securely by the research team, as indicated in the Participant Information Leaflet. | | | | | | | | | | | | | | | | | | | | | | | | | |  | |  | | | | |  | |  |
|  |  |  |  |  |  |  |  |  |  |  |  |  |  |  |  |  |  |  |  |  |  |  |  |  |  |  |  | *Initials* | | | | |  |  |  |
|  |  |  |  |  |  |  |  |  |  |  |  |  |  |  |  |  |  |  |  |  |  |  |  |  |  |  |  |  | | | | |  |  |  |
| 1. **I agree** to a member of the research team contacting me face-to-face, by telephone or post (or other agreed methods of contact including video/teleconference) about matters connected with the STRATA study by research and NHS staff. | | | | | | | | | | | | | | | | | | | | | | | | | |  | |  | | | | |  | |  |
|  |  |  |  |  |  |  |  |  |  |  |  |  |  |  |  |  |  |  |  |  |  |  |  |  |  |  |  | *Initials* | | | | |  |  |  |
|  |  |  |  |  |  |  |  |  |  |  |  |  |  |  |  |  |  |  |  |  |  |  |  |  |  |  |  |  | | | | |  |  |  |
| 1. **I consent (agree) to take part in the above study.** | | | | | | | | | | | | | | | | | | | | | | | | | |  | |  | | | | |  | |  |
|  |  |  |  |  |  |  |  |  |  |  |  |  |  |  |  |  |  |  |  |  |  |  |  |  |  |  |  | *Initials* | | | | |  |  |  |
|  |  |  |  |  |  |  |  |  |  |  |  |  |  |  |  |  |  |  |  |  |  |  |  |  |  |  |  |  | | | | |  |  |  |
| 1. **I am willing** to be contacted by a member of the research team about taking part in other future research. *Please tick either Yes* ***OR*** *No.* | | | | | | | | | | | | | | | | | | |  | | |  | | | | |  | | | |  | | | |  |
|  |  |  |  |  |  |  |  |  |  |  |  |  |  |  |  |  |  |  | **YES** | | |  | | | | | **NO** | | | |  | | | |  |
|  |  |  |  |  |  |  |  |  |  |  |  |  |  |  |  |  |  |  |  | | |  | | | | |  | | | |  | | | |  |
| 1. **I give consent** to be contacted by a member of the research team with a view to being interviewed about my reasons for accepting or declining study participation and, if relevant, experiences of taking part. I understand that my contact details will be passed to the researcher if required. *Please tick either Yes* ***OR*** *No.* | | | | | | | | | | | | | | | | | | | **YES** | | |  | | | | | **NO** | | | |  | | | |  |
|  |  |  |  |  |  |  |  |  |  |  |  |  |  |  |  |  |  |  |  |  |  |  | | | | |  |  |  |  |  | | | |  |
|  |  |  |  |  |  |  |  |  |  |  |  |  |  |  |  |  |  |  |  |  |  |  | | | | |  |  |  |  |  | | | |  |
| **You can still take part in this study if you answer ‘No’ to questions 11 and 12.** *Continue over page.* | | | | | | | | | | | | | | | | | | | | | | | | | | | | | | | | | | |  |
|  | | | |  |  | | | | | | |  |  | |  | **-** | | | |  |  | | **-** | | **2** | | | | **0** |  | |  | |  |  |
| **Your name in block capitals *(Participant)*** | | | |  | **Your Signature** | | | | | | |  | **Today’s Date (dd/mm/yyyy)** | | | | | | | | | | | | | | | | | | | | |  |  |
|  | | | |  |  | | | | | | |  |  | | | | | | | | | | | | | | | | | | | | |  |  |
|  | | | |  |  | | | | | | |  |  | |  | **-** | | | |  |  | | **-** | | **2** | | | | **0** |  | |  | |  |  |
| **Researcher name in block capitals**  ***(Person taking consent)*** | | | |  | **Researcher Signature** | | | | | | |  | **Today’s Date (dd/mm/yyyy)** | | | | | | | | | | | | | | | | | | | | |  |  |

**INFORMATION FOR RESEARCHER: When completed:** 1 copy for Investigator Site File together with a copy of the Participant Information Leaflet in recruitment order; 1 copy for medical notes with a supporting record of the discussion and a copy of the Participant Information Leaflet; 1 copy for participant; and 1 copy for the central research team (University of Bristol). See Protocol for full details.


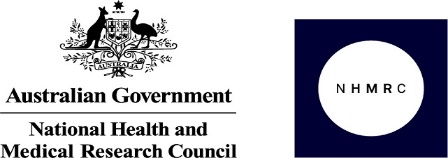

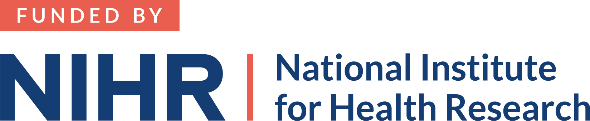


This study is funded by the National Institute for Health Research (NIHR) HTA Programme (Ref: 127337).The views expressed are those of the author(s) and not necessarily those of the NIHR or the Department of Health and Social Care.

The authors and University of Western Australia acknowledge funding from the National Health and Medical Research Council (Project Grant 1171206). The contents of the published material/website are solely the responsibility of the authors and do not reflect the views of NHMRC.
